# Synergistic Phenomena between Iron-Doped ZnO Nanoparticles and Shock Waves Exploited against Pancreatic Cancer Cells

**DOI:** 10.1021/acsanm.2c04211

**Published:** 2022-11-02

**Authors:** Marco Carofiglio, Marzia Conte, Luisa Racca, Valentina Cauda

**Affiliations:** Department of Applied Science and Technology, Politecnico di Torino, C.so Duca degli Abruzzi 24, 10129Turin, Italy

**Keywords:** zinc oxide, acoustic pressure wave, nanoparticle theranostics, stimuli-responsive therapy, pancreatic ductal adenocarcinoma

## Abstract

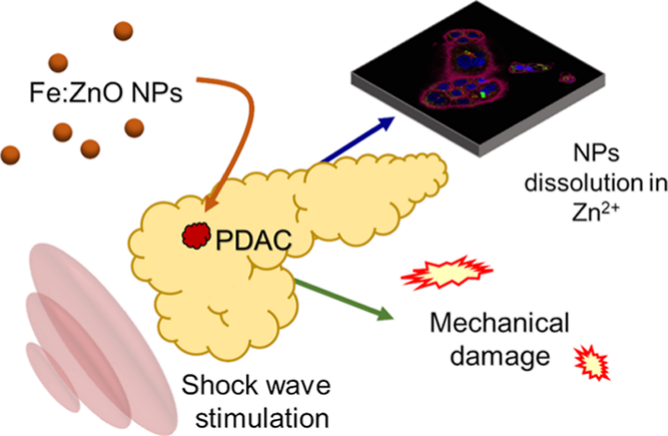

We propose the use of iron-doped zinc oxide nanoparticles (Fe:ZnO NPs) showing theranostic capabilities and being synergistically active against pancreatic ductal adenocarcinoma once combined with mechanical pressure waves, such as shock waves. Fe:ZnO NPs are synthesized by employing oleic acid as a capping agent and are functionalized with amino-propyl groups. We first report their superior characteristics with respect to undoped ZnO NPs in terms of magnetic properties, colloidal stability, cytocompatibility, and internalization into BxPC-3 pancreatic cancer cells *in vitro*. These Fe:ZnO NPs are also cytocompatible toward normal pancreatic cells. We then perform a synergistic cell treatment with both shock waves and Fe:ZnO NPs once internalized into cells. We also evaluate the contribution to the synergistic activity of the NPs located in the extracellular space. Results show that both NPs and shock waves, when administered separately, are safe to cells, while their combination provokes an enhanced cell death after 24 h. Various mechanisms are then considered, such as dissolution of NPs, production of free radicals, and cell membrane disruption or permeation. It is understood so far that iron-doped ZnO NPs can degrade intracellularly into zinc cations, while the use of shock waves produce cell membrane permeabilization and possible rupture. In contrast, the production of reactive oxygen species is here ruled out. The provoked cell death can be recognized in both apoptotic and necrotic events. The proposed work is thus a first proof-of-concept study enabling promising future applications to deep-seated tumors such as pancreatic cancer, which is still an unmet clinical need with a tremendous death rate.

## 1 Introduction

Theranostic nanosystems are acquiring more and more relevance in nanomedicine.^1^ The potential advantages offered by the administration of a site-specific therapy while constantly monitoring the patient conditions are actually enormous in the context of cancer, as for all other diseases which are very difficult to treat.^2,3^ Several prototypes of smart nanosized particles able to perform both these tasks can be found in the literature.^4−6^ One of the major trends includes the use of nanosystems incorporating imaging moieties and carrying anti-tumoral drugs,^7^ such as polymeric nanoparticles (NPs),^8−11^ liposomes,^12−14^ or mesoporous silica NPs.^15,16^ However, a main drawback of these devices is the inability to precisely control their release location, leading to an unwanted leakage of their cargo before correctly reaching the tumor environment.

New approaches feature NPs that can be activated to perform the therapy only once they have reached the site of interest. Recent advancements are going toward smart nanomaterials able to react to different physical and chemical stimulations. Examples include pH-sensitive materials capable of releasing anti-tumoral drugs only in the acidic tumor environment^17,18^ or magnetic NPs, such as the iron oxide ones,^19,20^ which can be remotely directed and excited to induce hyperthermia once inside the cancer tissues.^21−23^ Other classes of stimuli-responsive nanomaterials are NPs aimed at photothermal therapy^24−26^ (PTT) and photodynamic therapy^27^ (PDT). The underlying concept is to excite such NPs with a light source, in order to induce toxic phenomena in the nearby tissues. PTT consists in the excitation of optically responsive materials, that is, metal NPs, with an electromagnetic radiation, typically in the infrared region of the light spectrum. Once the light is absorbed, the excited material converts this energy into heat, which can ablate the tumor tissue and eventually cause cell death. Due to the electronic quantum confinement given by the nanoscale, gold NPs result extremely suitable for this purpose, as widely demonstrated by the large amount of related works in the literature.^28−31^ On the other hand, most of the PDT approaches rely on the generation of reactive oxygen species (ROS) upon light irradiation. ROS production is expected to induce toxicity on the diseased cells.^32^

Regardless of the energy sources used or the mechanism involved, the advantage of these remotely controlled NPs is their ability to activate a toxic phenomenon, provoking cell death exclusively on cancer cells and only once the therapeutic moiety has effectively reached its target.^33^

The use of remotely controlled NP approaches can face some drawbacks too. Indeed, the visible light possesses a reduced tissue penetration, while UV radiations may damage healthy tissues with side effects potentially overwhelming the benefits of the therapy.^34^ An alternate magnetic field, if too much intense, can provoke damages to normal tissues and create undesired eddy currents.^35^

Tissue penetration becomes a very important parameter to consider when treating deep-seated tumors, such as pancreas. Pancreatic cancer, and in particular the most common form of this disease, namely, pancreatic ductal adenocarcinoma (PDAC),^36^ is very difficult to treat due to the characteristics of its development, which provide it with resistance toward standard cancer treatments.^37,38^ Therapeutic approaches such as PDT could successfully address the difficult task of reducing tumor growth. However, the location of the pancreas inside the human body does not allow an effective stimulation with light. Different remote stimulations have been proposed as possible alternatives. An example is represented by the stimulation with periodic pressure waves, such as ultrasound (US) or shock waves (SWs), the latter being sharp discontinuities, that is, compressive and tensile waves, involving a sudden and strong change in pressure and density in a medium.^39,40^ Indeed, US represents a valuable alternative to light because pressure waves can penetrate deeper in the organic tissues interposed between the stimulation source and the target, while preserving a good level of safety on normal tissues when low-irradiation powers are employed. On the other hand, higher powers can be exploited for tumor thermoablation, as with focused US treatments.^41^ Several molecules have been proposed as enhancers of this phenomenon and are referred to as sonosensitizers, that is, systems able to induce hyperthermia or ROS generation only upon US stimulation so as to localize the therapy performed on the system.^42^ In this perspective, NPs such as gold,^43−45^ titanium oxide,^46,47^ and zinc oxide^48−51^ have been suggested as sonosensitizing agents and have shown a great potential in this field. The superiority of NPs with respect to organic sonosensitizer molecules appears clear and is at present extensively debated in the literature.^52^ NPs can be better dispersed in biological water-based media than organic molecules, and they can be biocompatible. NPs can accumulate in the tumor mass thanks to the enhanced permeation and retention effect or, even better, thanks to the use of functional biomolecules at their surface for the active targeting of cancer cells.^53^ Furthermore, NPs can be designed to act as theranostic tools and to be fully biodegradable at the end of their mission in the biological environment.

In this work, we propose the use of biocompatible, colloidal, biostable, and biodegradable NPs that display both imaging and therapeutic functionalities. We prove their effectiveness as sonosensitizing agents since they reduce pancreatic cancer viability *in vitro* when coupled with a remote mechanical stimulation, that is, SWs. More specifically, the subject of this work is represented by iron-doped zinc oxide NPs (Fe:ZnO NPs). They have been proved to possess good biocompatibility, magnetic properties, and therefore magnetic resonance imaging (MRI) potentialities in previous studies.^54^ The target of this first proof-of-concept test is a PDAC cell line (BxPC-3). Actually, PDAC can only be reached with a deep stimulation and displays innate and acquired drug resistance. It therefore urgently requires more effective therapeutic approaches, as the one here proposed. Fe:ZnO NPs are thus tested first in terms of their cytocompatibility, cellular uptake, and NPs dissolution in cell culture media in comparison to undoped ZnO NPs. Control tests with normal pancreatic cells were also carried out. The obtained results aim to increase the understanding of the Fe:ZnO NPs’ fate inside the cell, when their administration is not coupled with a remote physical stimulation, and to evaluate their final and safe biodegradation.

The coupling of the NPs with a mechanical stimulation, that is, pressure SWs, is exploited to induce cell death only on demand to achieve a remotely controlled and safe therapy. Therefore, BxPC-3 viability is assessed with the coupled treatment, and then the cell fate is observed to determine the cell death mechanism and other possible aspects that could be improved in view of a future clinical translation.

## 2 Materials and Methods

### 2.1 Synthesis of ZnO and Fe:ZnO NPs

Iron-doped ZnO NPs (Fe:ZnO NPs) were synthesized with a wet chemical method previously developed in other works.^54^ In particular, zinc acetate dihydrate (526 mg, 2.37 mmol, ACS Reagent, Sigma-Aldrich) and ferric nitrate nonahydrate (58 mg, 143 μM, HiMedia) were weighed to obtain a molar ratio of 0.06 between Fe and Zn ions. The salts were dissolved in 40 mL of ethanol (99%, Sigma-Aldrich) in a round-bottom flask under moderate stirring. Additionally, 1 mL of bidistilled water (from a Direct Q3 system, Millipore, Burlington) and 140 μL of oleic acid (≥99%, Sigma-Aldrich) were included in the solution to favor hydrolysis of the precursors and NP stability, respectively. The flask was heated up to 70 °C in a silicon bath under refluxing condition to limit solvent evaporation.

To favor the hydrolysis of the zinc precursor, according to the mechanism previously reported,^55^ 1.044 g of tetramethylammonium hydroxide (TMAH, 98.5%, Sigma-Aldrich) was dissolved in an ethanol/water solution (10 mL:1.052 mL). Then, TMAH was rapidly added to the zinc and iron precursors’ solution to provide the hydroxide required for the precursors’ hydrolysis. After 10 min, the formation of Fe:ZnO NPs was completed, and 40 mL of ice-cooled ethanol was added in the flask to stop the reaction. The whole dispersion was collected and centrifuged for 10 min at 8000*g*. The supernatant was discarded, and the NP pellet was redispersed in 40 mL of ethanol. This washing procedure was repeated three times to obtain the final batch of Fe:ZnO NPs.

**Figure 1:**
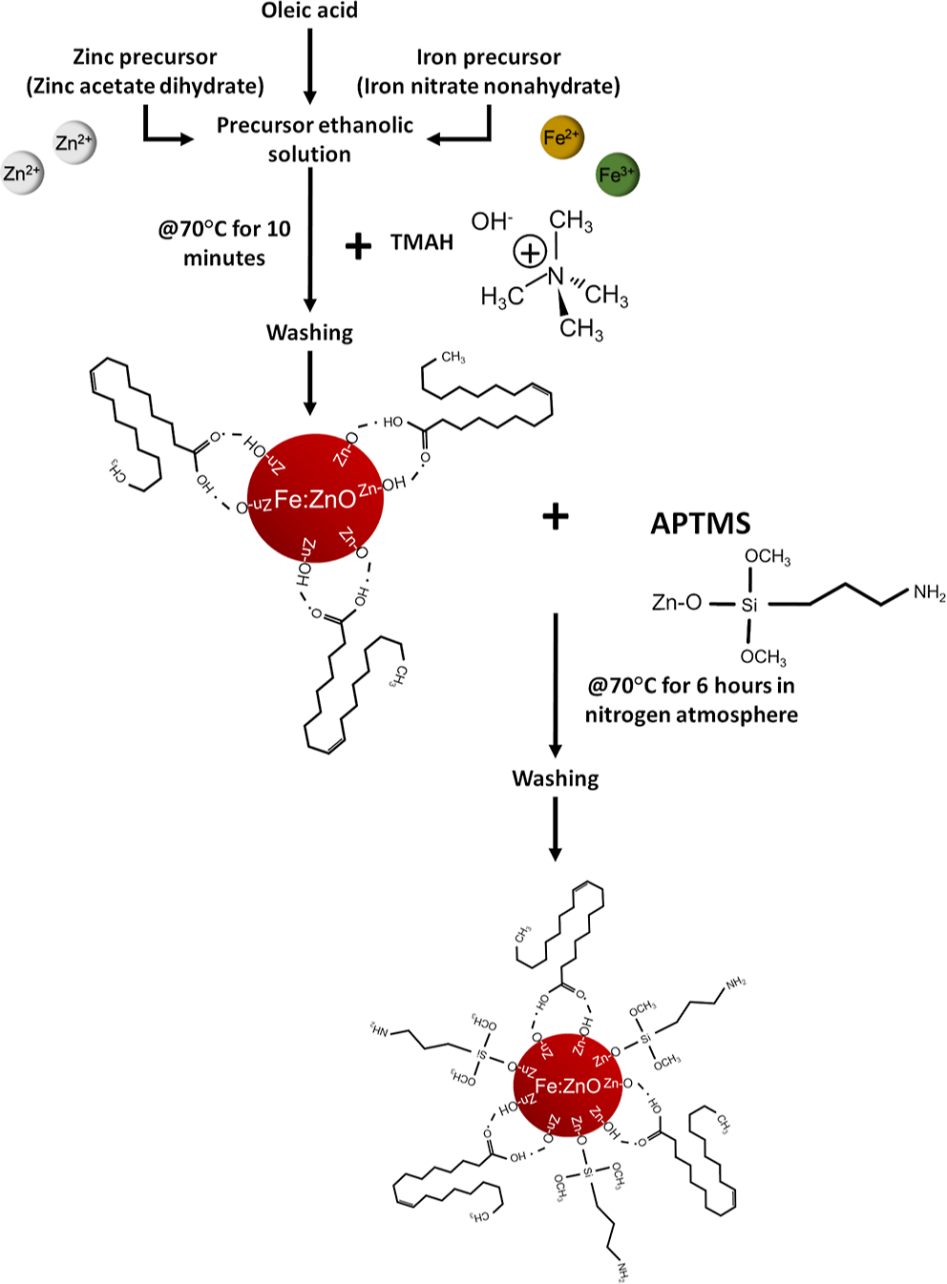
Fe:ZnO NP synthesis procedure scheme.

A scheme of the synthetic and functionalization procedures is reported in Figure 1.

The same procedure, without the inclusion of the iron precursor, was followed to synthetize undoped NPs, which were used as control.

### 2.2 Amino-propyl Functionalization

Undoped and Fe:ZnO NPs were also provided with an amino-propyl functionalization (Figure 1), as reported in other works.^55,56^ The functionalization was performed on 40 mg of NPs (either undoped or iron-doped) dispersed in 16 mL of ethanol to obtain a concentration of 2.5 mg/mL and placed in a 25 mL round-bottom flask. The solution was heated up to 70 °C under nitrogen refluxing condition. After 10 min of moderate stirring, 8.6 μL of 3-aminopropyltrimethoxysilane (APTMS, Sigma-Aldrich, corresponding to the 10% mol with respect to the ZnO amount) was added to the solution. The reaction was held for 6 h. The NP dispersion was finally collected and centrifuged at 14 000*g* for 20 min. The supernatant was discarded to remove the unreacted APTMS. Resuspension was performed with fresh ethanol, and the washing steps were repeated twice to obtain the batch that could be directly used in *in vitro* tests.

### 2.3 Physical and Chemical Characterization

#### 2.3.1 Dynamic Light Scattering Measurements

The prepared NPs were characterized from the physical and chemical standpoints. More in detail, dynamic light scattering (DLS) measurements of the hydrodynamic size were performed with a Zetasizer Nano ZS90 (Malvern Panalytical, Malvern, UK) both in water and in RPMI 1640 (cell culture medium, ATCC) supplemented with 10% vol of fetal bovine serum (FBS, ATCC) and 100 μg/mL streptomycin and 100 units/mL penicillin (P/S, Sigma-Aldrich). For the measurement, 100 μg of NPs was centrifuged for 10 min and redispersed in 1 mL of the medium of choice (either water or cell culture medium) by the help of a sonication bath (in detail, 10 min of sonication at 40 kHz with a Branson 3800 CPXH, Branson Ultrasonics Corporation). All the measurements were performed in triplicate. A similar sample preparation procedure was adopted to measure the NPs’ *Z*-potential in water, with the same instrument employed for DLS measurements.

#### 2.3.2 X-ray Diffraction Measurements

The crystallinity of the NPs was investigated by means of X-ray diffraction analysis. Undoped and iron-doped NPs suspensions for a total amount of 1 mg of NPs were dropped onto a monocrystalline silicon substrate and allowed to dry. The resulting film was analyzed by means of a Panalytical X’Pert diffractometer working in the Bragg–Brentano mode (Cu Kα source, λ = 0.154 nm, 30 mA, and 40 kV). The crystallite size was evaluated exploiting the software Origin (OriginLab).

#### 2.3.3 Electron Microscopy

Electron microscopy was used to determine the morphology of the synthesized NPs. For ZnO and Fe:ZnO, NPs were deposited onto a silicon substrate, allowed to dry, and analyzed with field emission scanning electron microscopy (FESEM, SUPRA 40, Zeiss), also equipped with an energy-dispersive X-ray spectroscopy (EDS) detector (x-act 10 mm^2^ silicon drift detector, Oxford Instruments) used for the elemental analysis and the determination of the amount of dopant element included in the nanocrystals.

Transmission electron microscopy (TEM) was also employed to characterize both ZnO and Fe:ZnO NPs. The samples were prepared by dispersing the NPs in water at a concentration of 25 μg/mL. Then, 10 μL of the solution was deposited onto a Lacey carbon support film (300 mesh, Cu, Ted Pella Inc.) and allowed to dry. The measurements were held with a Talos F200X G2 S(TEM) from Thermo Scientific at an operating voltage of 200 kV.

#### 2.3.4 ROS Generation under SW Stimulation Analysis

Fe:ZnO NPs were investigated in terms of ROS generation under remote mechanical stimulations using electron paramagnetic resonance (EPR) spectroscopy coupled with the spin-trapping technique. More in detail, 1.5 μg of Fe:ZnO NPs was withdrawn from the ethanolic stock solution and dispersed in 90 μL of water. The dispersion was placed in a 96-well plate for cell culture (TC-treated, Corning). Then, 10 μL of a water solution of a spin trap (5,5-dimethyl-l-pyrroline-*N*-oxide, DMPO, Sigma-Aldrich) at a concentration of 100 mM was added to the solution. The final concentration of Fe:ZnO NPs was 15 μg/mL, while the DMPO final concentration for each sample was 10 mM. The Fe:ZnO NP-containing well was stimulated from the bottom with a high-energy focalized SW device PW^2^ (R. Wolf, ELvation Medical) at an energy flux density of 0.04 mJ/mm^2^ (12.5 MPa as the maximum pressure peak) and a number of shots equal to 500 with a frequency of 4 shots/s. The well and the transducer were acoustically coupled with a gel (Stosswellen Gel, ELvation Medical GmbH). After the stimulation, a small volume of the NP dispersion was withdrawn by means of a quartz capillary and analyzed with an EMXNano X-band spectrometer (Bruker, center field 3426 G, 10 scans, 60 s sweep time). The spectra were processed with Bruker Xenon software (Bruker).

#### 2.3.5 ZnO and Fe:ZnO Dissolution in Cell Culture Medium

The dissolution of Fe:ZnO NPs into zinc cations in cell culture medium at 37 °C was also evaluated. Fe:ZnO NPs at a concentration of 1 mg/mL were dispersed in cell culture medium. Then, 70 μL of this dispersion was placed in the cap of a 1.5 mL centrifuge tube, opportunely separated by a dialysis membrane (SnakeSkin dialysis tubing 3.5k MWCO, 16 mm dry I.D. by Thermo Fisher Scientific) from the rest of the tube to avoid NP leakages. Then, 630 μL of cell culture medium was added in the tube, in contact with the NP solution through the dialysis membrane so as to bring the NP concentration in the overall solution to 100 μg/mL and to allow only ion exchange. The tube was placed upside down in an orbital shaker at 37 °C for different incubation time steps (up to 72 h). At the end of each incubation time, the solution contained in the main tube (*i.e.*, without NPs) was withdrawn and stored at 4 °C up to further analysis. The amount of zinc atoms (derived from dissolved zinc cations) was evaluated through graphite furnace atomic absorption spectroscopy following the EPA method 289.1.

### 2.4 Cell Culture, Viability, and Internalization Assays

#### 2.4.1 Fe:ZnO and ZnO Cytotoxicity

Fe:ZnO and ZnO NPs were tested in terms of cytotoxicity on a PDAC cell line, that is, BxPC-3 (ATCC CRL-1687) and on the healthy counterpart (HPDE-H6c7, human pancreatic duct epithelial cells, Kerafast, Inc., Boston, MA). For daily cell culture, cells were cultured at 37 °C and 5% of CO_2_ in RPMI 1640 medium (ATCC) supplemented with 10% vol of previously heat-inactivated FBS (ATCC), 100 μg/mL of streptomycin, and 100 units/mL of penicillin (Sigma-Aldrich).

Cytotoxicity tests were performed in 96-well plates (TC-treated, Corning) with the WST-1 proliferation assay (Roche). More in detail, 2500 cells dispersed in 100 μL of cell culture medium were seeded in each well and allowed to adhere on the bottom of the well. After 24 h of incubation, Fe:ZnO and ZnO NPs were directly taken from the ethanolic stock solution (2 and 1 mg/mL for Fe:ZnO and ZnO NPs, respectively) and dispersed at different concentrations in fresh culture medium (10, 15, 20, and 25 μg/mL of NPs). The NP dispersions were then administered to cells by simple cell culture medium substitution.

The cytotoxicity was evaluated at different times of incubation (24, 48, and 72 h). In particular, 10 μL of WST-1 reagent was added to each well 2 h before the expiration of the incubation time step in question. A plate reader (Multiskan GO microplate spectrophotometer, Thermo Fisher Scientific) was exploited for the measurements. More in detail, the absorbance of the samples was evaluated at 450 nm (*A*_450_), while the one at 620 nm (*A*_620_) was used as a reference. For all the samples, the absorbance value obtained by the cell culture medium with no cultured cells (BK_450_–BK_620_) was subtracted from the actual measure; the resulting value was then divided by the absorbance of the control cells (CT_450_–CT_620_), in order to obtain a percentage value, as reported in other works.^46,50^ More explicitly, the formula leading to the cell viability percentage evaluation (*C*_%_) is
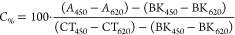


#### 2.4.2 Fe:ZnO and ZnO Internalization in the BxPC-3 Cell Line

The internalization of Fe:ZnO and ZnO NPs in BxPC-3 cells was evaluated through flow cytofluorimetry. To do so, 3 × 10^5^ cells were seeded in a 24-well plate (TC treated, Thermo Fisher) with 500 μL of cell culture medium. After 24 h from cell plating, the medium was replaced with cell culture medium containing NPs (10 μg/mL for pure ZnO NPs and 10 and 15 μg/mL for Fe:ZnO NPs). The NPs had been previously labeled with ATTO647-NHS ester, as described in a previous work,^57^ and their uptake by BxPC-3 cell line was evaluated after 5 and 24 h. The aim was to determine the timing at which the majority of BxPC-3 was able to internalize the administered NPs, to maximize the effects of further treatments. At the end of the incubation time, the cells were washed twice with phosphate-buffered saline (PBS) to remove non-internalized NPs and then detached through trypsinization. Once collected, the cells were centrifuged (130*g* for 5 min) and resuspended in 300 μL of PBS. The cell suspension was analyzed through a Guava easyCyte 6-2L flow cytometer (Merck Millipore), following the same procedure already described elsewhere.^50^ The analysis of the results was performed with InCyte software (Merck Millipore).

All biological tests were performed at least in triplicates, and ANOVA tests were performed with the software Origin (OriginLab).

The internalization of Fe:ZnO NPs inside BxPC-3 cells was also analyzed through spinning-disk confocal fluorescence microscopy (Ti2 Nikon equipped with a crest large FOV laser and a 60× PlanAPO objective, NA = 1.40) to locate the position of the NPs inside the cell. To do so, 1 × 10^4^ cells were seeded into eight-well chamber slides (Nunc Lab-Tek II CC2 Chamber Slide system, Thermo Fisher Scientific) with 250 μL of complete cell culture medium. ATTO647-NHS-labeled Fe:ZnO NPs were then administered to cells (15 μg/mL) 24 h from seeding. After 24 h , the cells were fixed by replacing the cell culture medium with 150 μL of Image-IT fixative solution (Thermo Fisher). After 10 min at room temperature, the cells were washed twice with PBS solution, and their membranes were stained by incubating them with 250 μL of PBS containing wheat germ agglutinin conjugated with an Alexa Fluor 488 dye (WGA-488, Thermo Fisher) at a concentration of 2.5 μg/mL for 10 min in normal cell culture conditions. After this time, the cells were washed twice with PBS, and then Hoechst (Thermo Fisher), at a concentration of 0.3 μg/mL in PBS, was administered to the cells for nuclei staining. After 5 min at 37 °C, the cells were washed twice with PBS, and live cell imaging (LCI, Molecular Probes) solution was finally added. The samples were immediately analyzed following staining.

### 2.5 SW Treatment

The behavior of BxPC-3 cells subjected to mechanical stimulation coupled with the administration of the safe dose of Fe:ZnO NPs was analyzed. The cells were seeded in a 96-well plate adopting the same procedure previously described, following a specific plate layout designed to avoid cross-mechanical stimulation, that is, leaving at least one well without cells between two nearby samples.

The cells were treated with the highest safe dose of Fe:ZnO NPs (15 μg/mL) and, after 24 h from NP administration, they were mechanically stimulated with the PW^2^ SWs generator. The same power conditions and stimulation times exploited during ROS generation analysis were here employed (0.04 mJ/mm^2^, 500 shots, 4 shots/s). In particular, three main stimulation typologies were set. In the first one, cells were only treated with the SWs, in the absence of NPs, to assess the toxicity of the SW treatment itself. The second typology consisted in the stimulation of cells that were first treated with Fe:ZnO NPs and then deprived of non-internalized NPs by means of a double washing with PBS and its replacement with freshly prepared cell culture medium just before the SW treatment (Fe:ZnO Int). Finally, the third typology considered the stimulation of cells that were treated with Fe:ZnO and mechanically stimulated after 24 h from the NP administration without any washing steps (Fe:ZnO Int + Ext). This last sample aimed at the exploitation of both intracellular and extracellular NPs for cell killing.

The BxPC-3 cell viability was assessed with the WST-1 assay 24 h after the mechanical stimulation and compared to that of untreated cells, set as 100% of viability.

### 2.6 NP Dissolution Test and Cell Death Mechanism Assays

#### 2.6.1 Free Zinc Cation Detection in Cells without Mechanical Stimulation

An evaluation of the free zinc cations, due to intracellularly dissolved NPs after their internalization and in the absence of any mechanical treatment, was initially performed. The cells were plated in chamber slides, treated with Fe:ZnO NPs, and fixed (see the details above). The intracellular free zinc cations were labeled through the FluoZin-3 AM (Thermo Fisher) probe: the cells were washed twice with PBS and a 1 μM solution of FluoZin-3 AM fluorescent dye in PBS was administered. After 30 min of incubation at 37 °C, the excess of dye was removed by two washing steps with PBS. Then, cell lysosomes were labeled by substituting the PBS with a 1 μM dye solution (LysoTracker Red DND-99, Thermo Fisher) in PBS and incubating the cells for 30 min at room temperature. After two further washing steps, the cell membranes and cell nuclei were stained with WGA-647 (Thermo Fisher) and Hoechst, respectively, following the same procedure reported above. The images were collected with a spinning disk confocal microscope, keeping the exposure times and laser powers constant among the different samples. They were then post-processed in the same way to allow a direct comparison between the samples in terms of free zinc intensity fluorescence too.

#### 2.6.2 Effect of SW Mechanical Stimulation on BxPC-3 Cell Fate

To unravel the mechanism of cell death under SW stimulation, the cells were plated in clear 96-well plates following the same protocol exploited for cytotoxicity assays. The cells were then treated with Fe:ZnO NPs and SWs. Free zinc cations were labeled again with FluoZin3-AM, while the cell membrane integrity was assessed with propidium iodine (PI, Thermo Fisher) using 100 μL of 1 μM PI solution in cell culture medium for 5 min at 37 °C. Then, the cells were washed three times with PBS. PBS was finally substituted with LCI solution, and cells were imaged with a wide-field inverted fluorescence microscope (Eclipse TiE from Nikon) equipped with a 40× objective (NA = 0.60).

#### 2.6.3 Kinetics of BxPC-3 Cell Necrosis and Apoptosis

The kinetics of both cell necrosis and apoptosis was evaluated through the RealTime-Glo Annexin V apoptosis and necrosis assay (Promega) coupled with the microplate reader GloMax (Promega). More in detail, BxPC-3 cells were plated in a black 96-well plate with a clear bottom (Corning) and treated with the same protocol exploited for SW treatments in the previous tests. Just before the SW treatment, 100 μL of the 2× detection reagent freshly prepared in RPMI 1640 according to the manufacturer indications was added to all the samples. A first measurement was performed before SW treatments. Then, the measurements were repeated immediately after the SW treatment and after 30 min, 1 h, 2 h, 4 h, 6 h, and 24 h from the treatment.

Both the fluorescence and the luminescence signals retrieved from the measurements were analyzed, as recommended by the manufacturer. In particular, the obtained data were processed as fold induction of the signal obtained from the cells without any NPs and those subject to SW treatment, according to the formula: sample signal/control signal.

## 3 Results and Discussion

### 3.1 Physical and Chemical Characterization

**Figure 2:**
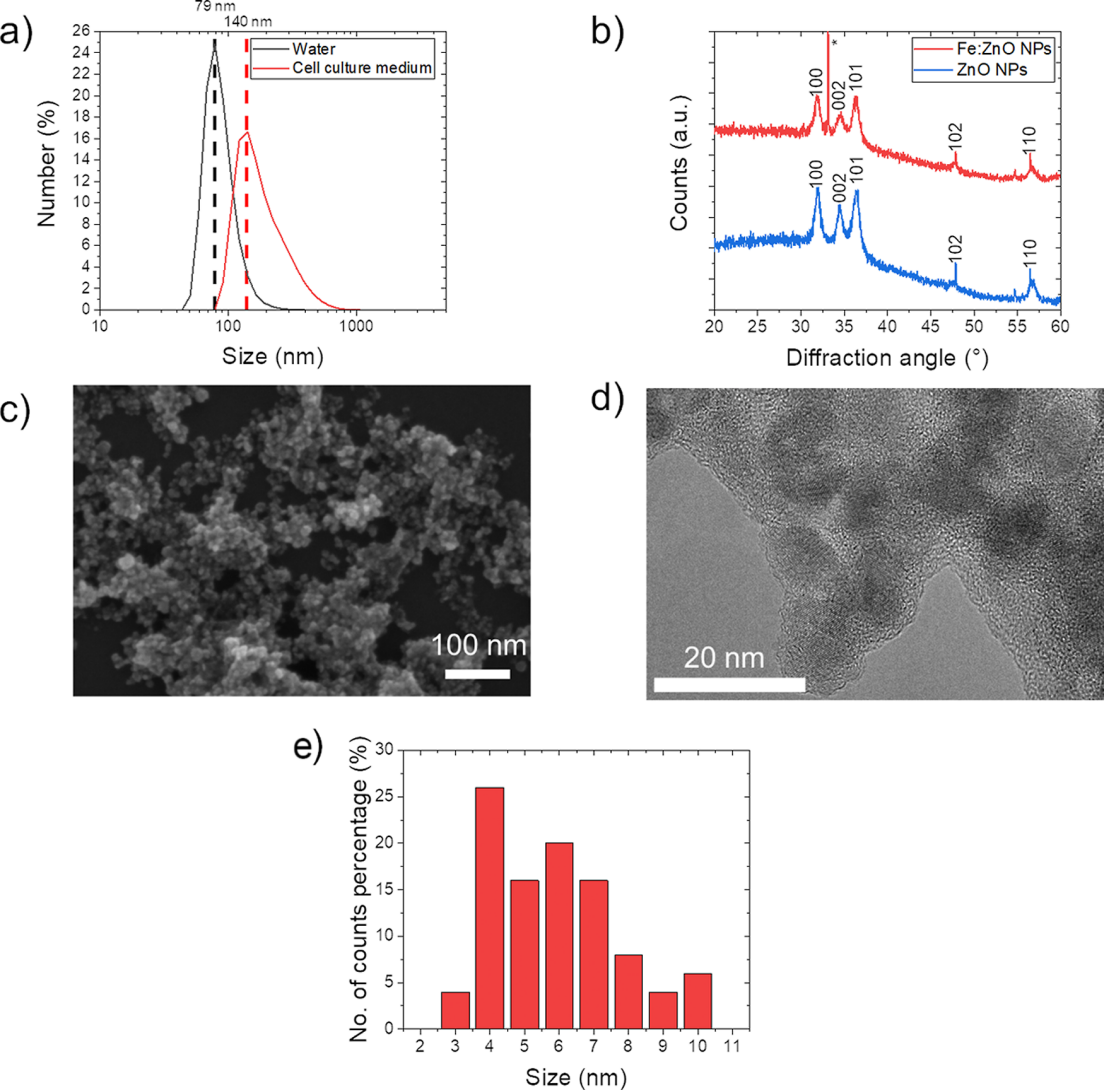
Fe:ZnO NP characterization: (a) hydrodynamic diameter distribution in bidistilled water and cell culture medium obtained by DLS measurements; (b) XRD pattern (* refers to peaks belonging to the silicon substrate); (c) representative FESEM image, (d) representative TEM image, and (e) histogram of TEM size of Fe:ZnO NPs.

Fe:ZnO NPs were characterized in terms of crystalline structure, morphology, and hydrodynamic behavior in both water and cell culture medium (Figure 2) and compared to their undoped counterpart ZnO NPs (Figure S1 in the Supporting Information). Fe:ZnO NPs present an average hydrodynamic diameter weighted on the scattered light intensity of 136.6 nm with a polydispersity index (PDI) of 0.138, indicating a monodisperse population.^55^ A higher diameter is observed when NPs are immersed in the cell culture medium. Indeed, the hydrodynamic diameter observed with the DLS is 249.6 nm. The reason of this behavior may be attributed to a larger degree of aggregation of the NPs in a more complex medium than pure water, caused by the interaction of the Fe:ZnO NPs with proteins, amino acids, and biomolecules in general.^56^ The PDI in cell culture medium is higher than that obtained in water (PDI of 0.264 and 0.110, respectively), indicating a higher polydispersity, which is however still acceptable to guarantee a homogeneous NP solution to be administered to the target cells.

A similar trend is observed for the undoped ZnO NPs, which present a hydrodynamic diameter of 116.2 nm in water and 386.6 nm in cell culture medium, with a PDI of 0.148 and 0.394, respectively (Figure S1 of the Supporting Information). When measured in water, the size of undoped ZnO is lower than that of the iron-doped NPs. As previously stated, instead, the NP behavior in the cell culture medium may be influenced by the interaction with various biomolecules and therefore results in a lower stability. In particular, undoped NPs possess both a higher PDI and hydrodynamic diameter with respect to the Fe:ZnO ones. This difference in stability is further corroborated by the *Z*-potential measured for the two typologies of NPs: 25.9 ± 0.9 mV for Fe:ZnO NPs and 22.7 ± 0.9 mV for ZnO NPs. The higher and positive *Z*-potential presented by the doped NPs is very likely increasing the Coulombic repulsion among the NPs, enhancing in turn their colloidal stability in solution.

The crystalline structure of Fe:ZnO and pure ZnO NPs was investigated through XRD analyses. In both cases, the XRD patterns (Figure 2b for Fe:ZnO and Figure S1b for ZnO NPs) present the typical diffraction peaks ascribable to the wurtzite crystal structure of ZnO (JCPDS-ICDD, card no. 89-1397). Either way, the peaks are quite broadened, having a crystallite dimension of 10 and 11 nm, estimated for the 100 plane by means of the Debye–Scherrer formula,^58^ for Fe:ZnO and ZnO NPs, respectively. The results also show no major differences in the crystalline structure caused by the introduction of defect sites due to iron doping. Thus, the Fe:ZnO pattern confirms the absence of any peaks related to secondary phases of possible other iron compounds.

The crystallite dimensions calculated through the XRD analysis are in agreement with the electron microscopy images of Figure 2c,d. The morphology assumed by Fe:ZnO NPs is spherical, with a diameter ranging from 4 to 10 nm calculated by FESEM and TEM (as depicted by the histogram in Figure 2e). This size range corresponds to the crystallite dimension obtained from XRD and allows the assumption of monocrystalline NPs. This is also confirmed by the TEM image (Figure 2d), in which some of the nanocrystals can be recognized. The discrepancies between the diameter found by means of electron microscopy and DLS measurements should be attributed to a certain level of aggregation that the NPs may display in the liquid media; moreover, the two instruments measure different parameters, namely, the actual and the hydrodynamic diameter of the NPs. In this case as well, from the morphological point of view, no visible difference between doped and undoped NPs can be appreciated.

To confirm the correct inclusion of iron in the ZnO NPs, EDS was performed. The results, reported in Table S1 of the Supporting Information, show that the atomic ratio between iron and zinc atoms inside ZnO crystals is 0.0507. This result is fairly in agreement with the precursor ratio exploited during the synthesis (0.06) and confirms the inclusion of the doping elements inside the crystalline structure, in accordance with both the results obtained by XRD analyses and a previous study concerning iron-doped NPs, synthetized by some of our group.^54^ In the work in question, it was also demonstrated that iron assumes both the Fe^2+^ and Fe^3+^ states, with a slight preponderance of Fe^3+^ ions for the NPs doped with 6 at. % of iron with respect to zinc.

Moreover, the inclusion of iron inside the Fe:ZnO NP crystal gathers novel magnetic responsiveness with respect to the pure ZnO NP counterparts (as reported in Figure S2 of the Supporting Information) in terms of maximum magnetization, with consequent potentialities in MRI. This result is in agreement to what was found in the literature, where this magnetic behavior is attributed to the introduction of magnetic atoms (Fe) in the ZnO crystal.^54,59−61^

### 3.2 Cytotoxicity of Pure and Iron-Doped ZnO NPs

**Figure 3:**
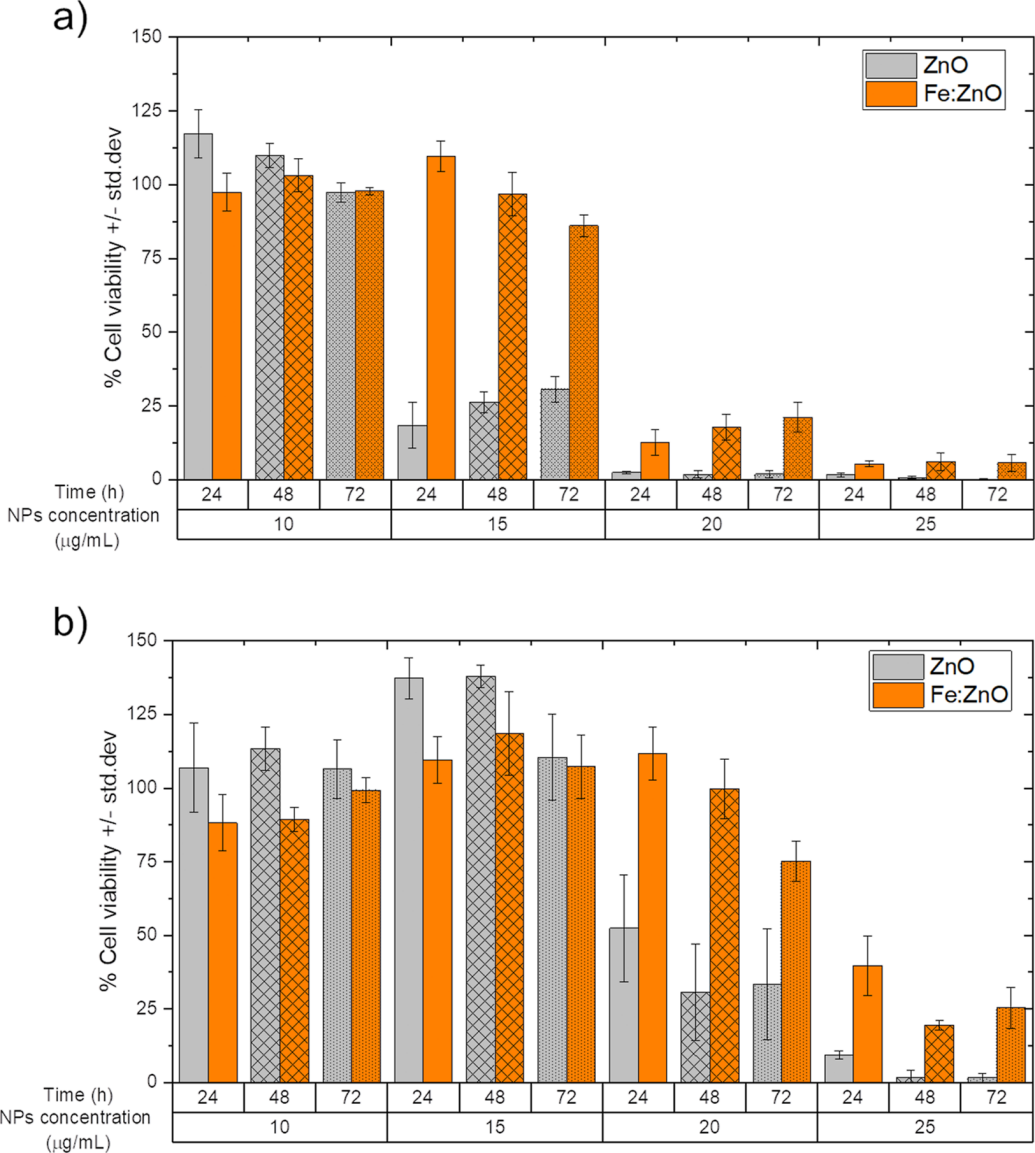
ZnO and Fe:ZnO NP cytotoxicity on (a) BxPC-3 and (b) HPDE-H6c7 cells at different incubation times from NP administration calculated from the results of the WST-1 assay. The value reported by the bars represents the mean ± std. dev. percentage of *n* ≥ 3 measurements with respect to control cells. The statistical analysis results are reported in the Supporting Information.

Fe:ZnO and ZnO NPs were administered to BxPC-3 cells in order to assess their cytotoxicity and understand which dose can be exploited for further treatments with acoustic stimulation. As a general trend, both NP types show a dose-dependent cytotoxicity which does not vary sensibly depending on the incubation time, as shown in Figure 3a. In particular, the undoped ZnO NPs are safe up to 10 μg/mL for all the considered incubation times in the present study. In contrast, the toxicity abruptly increases already at 15 μg/mL, with cell viability close to 20% compared to the control samples. Moreover, a slight viability increase in time can be observed, with values always below 40%.

A similar trend is observed for Fe:ZnO NPs; however, the highest safe dose is extended up to 15 μg/mL for the time steps considered in the present study. The dose-dependent toxicity of ZnO is already well known in the related literature^57,62,63^ and has been mainly attributed to two mechanisms: ZnO dissolution into Zn^2+^ ions, able to disrupt the cell homeostasis leading to cell death, and the oxidative stress generated by the NPs due to ROS production. Iron doping has been demonstrated to reduce the dissolution of ZnO NPs, providing them with a higher stability in aqueous media.^64,65^ This translates into a higher safety of the NPs, which has also been evidenced in this work when Fe:ZnO NPs are compared to their undoped counterparts. A lower toxicity also opens the possibility of administering higher doses of NPs that are therefore more likely to induce toxic phenomena under external stimulation (*i.e.*, SWs).

In this particular case, the highest safe dose, defined as the amount of NPs that can be administered without significantly damaging cells prior external stimulation, is increased by 50% thanks to the iron doping (*i.e.*, from 10 to 15 μg/mL). Furthermore, the iron-doped ZnO NPs show magnetic properties, not present in the undoped counterpart, as reported in previous works.^59,60,66^ This is also true for the herein-analyzed Fe:ZnO NPs, whose maximum magnetization is higher than the one measured for pure ZnO NPs (Figure S2). Such magnetic property is exploitable to employ them as contrast agents under MRI, yet leading to an intrinsically theranostic NP. For all these reasons, only iron-doped NPs will be investigated henceforth when the mechanical stimulation is considered.

Despite a proper targeting mechanism should be implemented on the NP surface, cytotoxicity tests on non-cancerous cells demonstrated an intrinsic selectivity of the NPs toward cancer cell killing. Figure 3b reports the results of the cytotoxicity experiments held on normal cells (HPDE-H6c7 cells). As it is clearly visible, with respect to what was found for cancerous cells, a higher dose of both ZnO and Fe:ZnO NPs can be administered to normal cells without affecting normal cell viability. Moreover, Fe:ZnO NPs are confirmed to be less toxic than ZnO NPs for normal cells too, further justifying their use during future treatments.

### 3.3 Fe:ZnO NP Internalization

ZnO and Fe:ZnO NPs were also investigated from the internalization standpoint. The ability of the BxPC-3 cell line to internalize the administered NPs was analyzed with cytofluorimetry and fluorescence microscopy.

**Figure 4:**
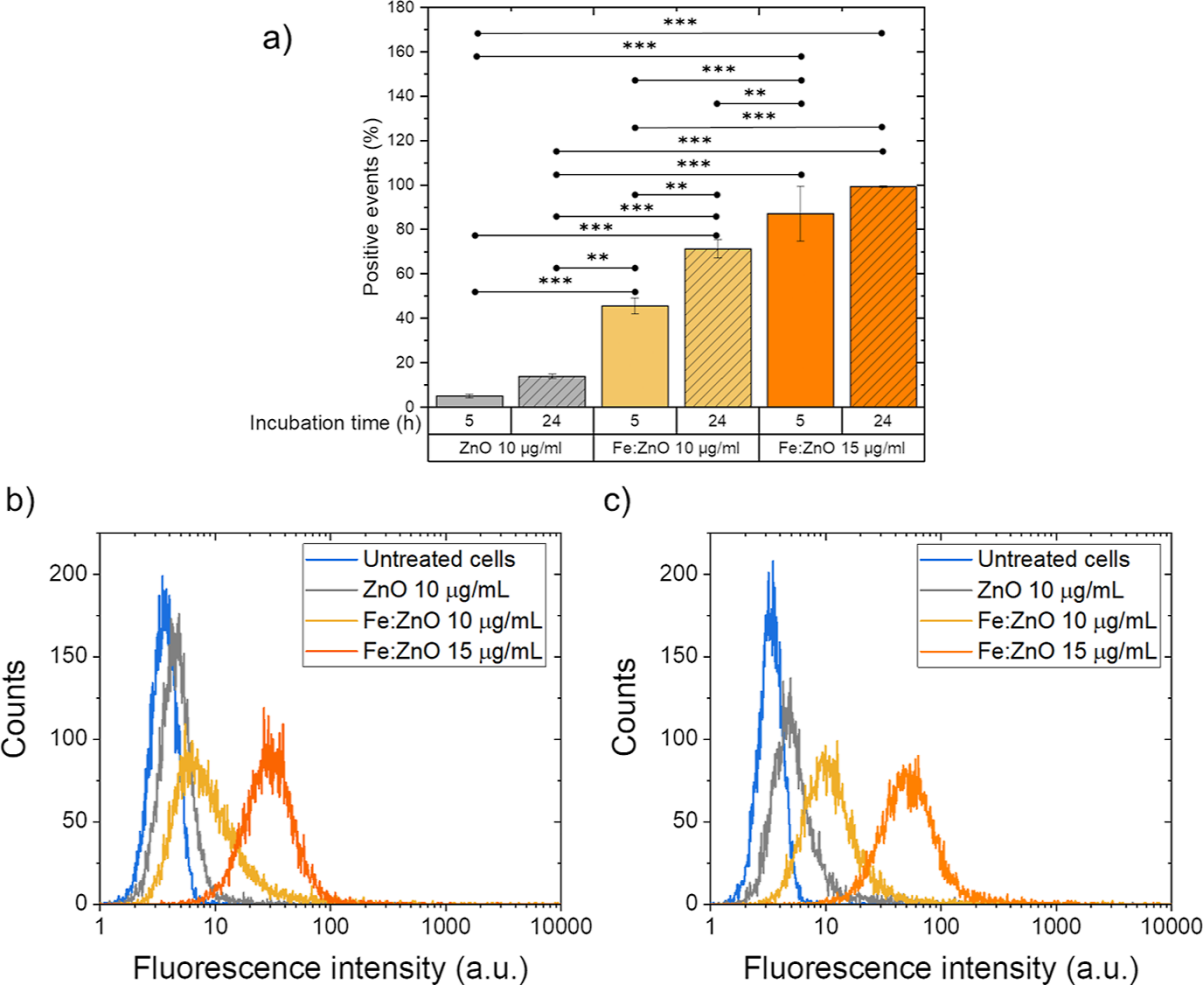
(a) BxPC-3 cells measured as positive events due to the internalization or immobilization at the outer cell membrane of ZnO and Fe:ZnO NPs. Data are reported at different incubation times from NP administration calculated through cytofluorimetric assays. The values reported by the bars represent the mean ± std. dev. percentage of *n* = 3 measurements with respect to control cells. The comparisons between the different treatments were performed using two-way ANOVA. ***p < 0.001, **p < 0.01, and *p < 0.05. Representative histograms of the fluorescence intensity of cells measured through the cytofluorimetric assays exploited to evaluate the NP internalization at (b) 5 and (c) 24 h from NP administration.

In particular, cytofluorimetric assays (Figure 4) indicate that a higher percentage of cells (positive events) is able to internalize (or adsorb at their surface) the Fe:ZnO NPs when compared to pure ZnO NPs. This behavior was already reported in similar conditions by our group.^54^ It has also been reported in the literature that a high positive *Z*-potential can favor NP internalization into cells, which typically possess a negatively charged cell membrane.^67−69^ Here, the higher *Z*-potential of iron-doped NPs, together with the larger exploitable dose due to their increased safety, may explain the superior internalization in cells with respect to the undoped ZnO.

From the results of the cytofluorimetric analysis, it can be observed that the percentage of positive events, namely, cells with an increased fluorescence, improves with time and dose. In particular, the percentage of cells with a higher fluorescence signal than the control cells approaches 100% after 24 h from 15 μg/mL Fe:ZnO NP administration, indicating that all the cells exhibit NPs on their cell membrane or inside of them. This result justifies and supports the use of these NP dose and incubation time for further mechanical stimulation with SWs in cells.

**Figure 5:**
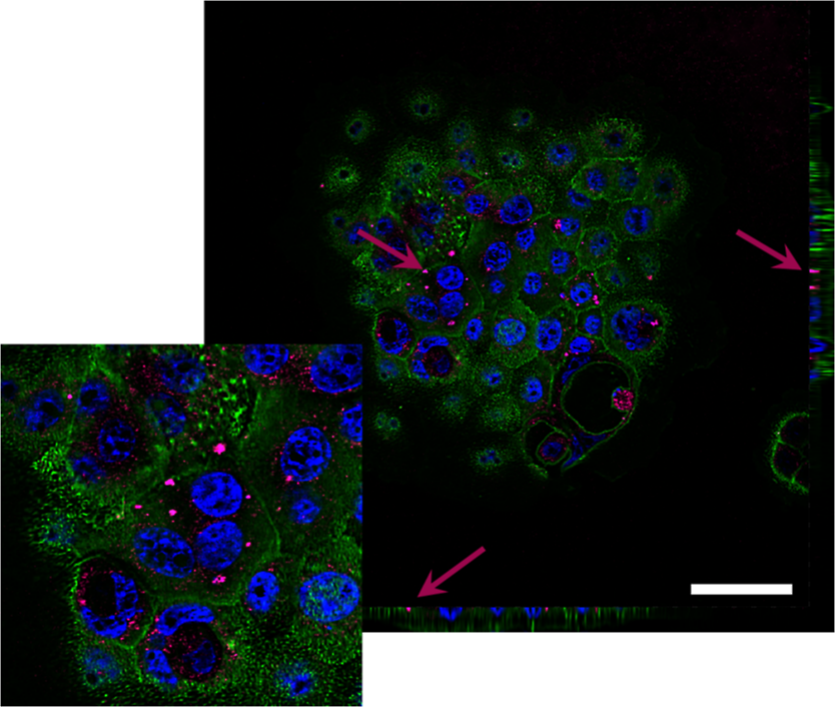
Representative image of Fe:ZnO NPs internalized in BxPC-3 pancreatic cancer cells after 24 h of incubation from NP administration. Cell membranes (green), nuclei (blue), and Fe:ZnO NPs (purple) are evidenced in the image. The scale bar is 50 μm.

Spinning disk confocal fluorescence microscopy was exploited to localize Fe:ZnO NPs inside the cells. Figure 5 reports a representative image of BxPC-3 cells incubated with 15 μg/mL Fe:ZnO NPs. Despite the nanosized nature of the NPs, which dramatically reduces the possibility to clearly resolve them, it is still possible to locate the presence of some aggregates inside the cells, thus obtaining information about the NP intracellular location. In particular, the majority of the NPs can be spotted inside the cell membrane but outside the cell nucleus, suggesting that the internalization process allows the permeation of the NPs only inside the cytoplasm.

### 3.4 Effects of SWs Coupled with Fe:ZnO NPs on Pancreatic Cancer Cells

The final aim of the proposed NPs is to provide a tool that can be externally activated by means of a remote mechanical stimulation to induce cell death in a diseased tissue. Pancreatic cancer cells were exploited as a tumor model for this purpose. Indeed, the highest safe dose of Fe:ZnO NPs was administered to cells and, after 24 h of incubation, the cells were stimulated with SW in two different conditions: in the presence of NPs internalized in the cells and in the extracellular space, that is, in cell culture medium (Int + Ext NPs) or with internalized NPs alone (Int NPs).

**Figure 6:**
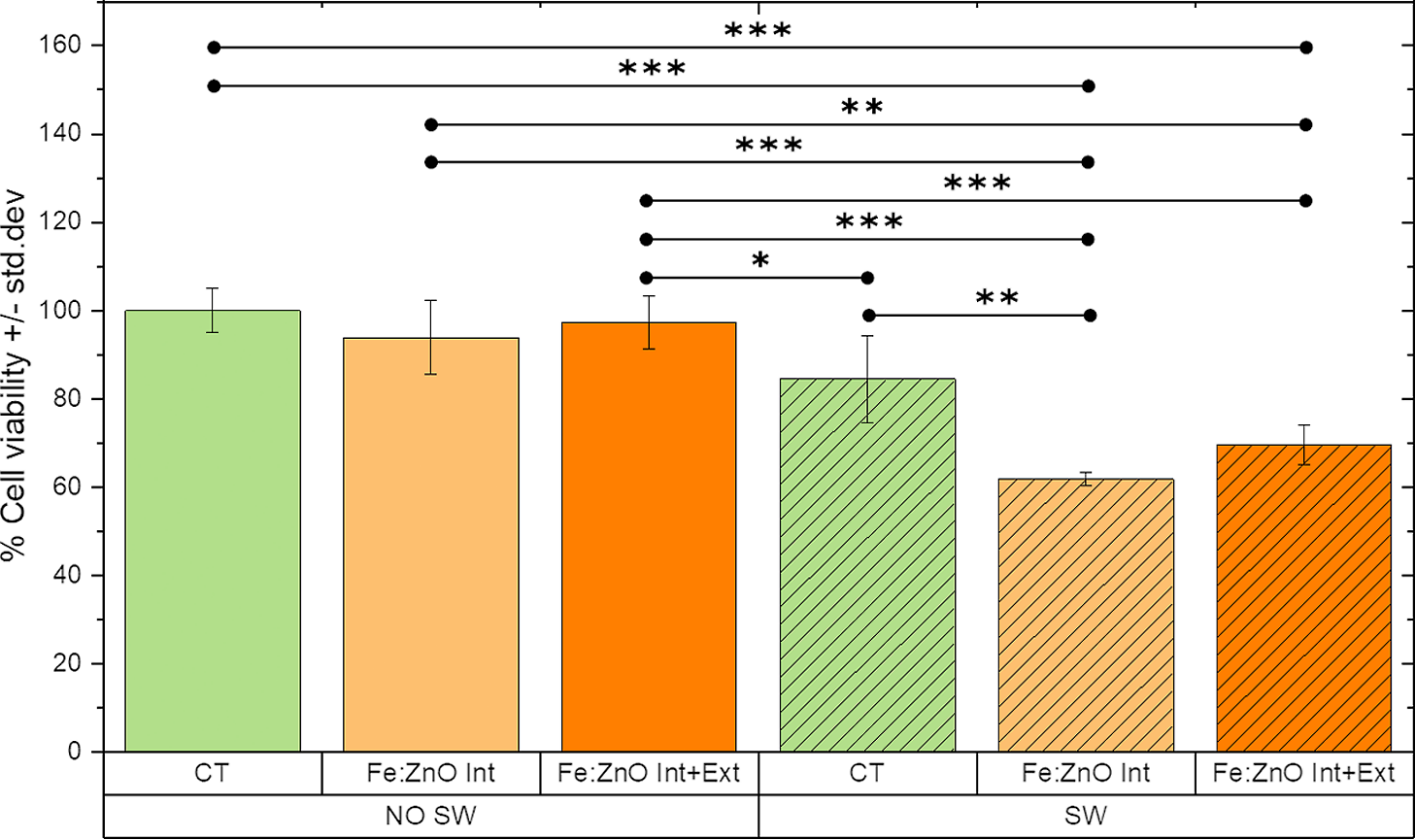
Cell viability percentages, with respect to untreated BxPC-3 cells, in the presence of Fe:ZnO NP and SW stimulation. The graph shows the difference between SW-treated (SW in the graph) and untreated (NO SW in the graph) cells and the difference among control cells (CT), cells treated with only internalized NPs (Fe:ZnO Int in the graph), and with NPs internalized in cells and in the extracellular space (Fe:ZnO Int + Ext in the graph). The values reported by the bars represent the mean ± std. dev. percentage of *n* ≥ 3 measurements with respect to control cells. The comparisons between the different treatments were performed using three-way ANOVA. ***p < 0.001, **p < 0.01, and *p < 0.05.

The cell viability was evaluated after 24 h from NP incubation and mechanical stimulation (Figure 6). As expected from the cytotoxicity tests, Fe:ZnO NPs are safe when no SWs are co-administered. Furthermore, there is no difference in viability between the cells where only internalized NPs are considered (Fe:ZnO Int) and those where both internalized and non-internalized NPs were retained (Fe:ZnO Int + Ext). Also, the mechanical stimulation with SW does not significantly affect the cell viability per se, that is, when NPs are not administered to cells.

Remarkably, a statistically higher toxicity with respect to the control samples is achieved when the two treatments (*i.e.*, NP and SW administration) are combined together. In particular, the viability of cells treated with Fe:ZnO Int and SW is 61.8 ± 1.5% with respect to the untreated cells; furthermore, a statistically relevant difference between this synergistic treatment and either the single NP administration or the solely SW stimulation is evidenced. When the cells are treated with SW and Fe:ZnO Int + Ext, the viability is 69.6 ± 4.5% with respect to the control. The difference among the two types of NP treatments is not statistically significant in terms of cell viability. This result suggests that most of the toxic effects could be attributed to the internalized NPs rather than to the extracellular ones. However, to determine the actual mechanism involved in cell death, further studies are required, as reported below.

### 3.5 Toxicity Mechanisms

There are typically three toxicity mechanisms that could be involved in cell death due to the presence of both ZnO NPs and SWs: (i) ROS generation and consequent oxidative stress, (ii) ZnO dissolution in Zn ions, and (iii) mechanical cell membrane disruption due to the mechanical wave and the presence of NPs. Here, we have investigated all of them to unravel the mechanism of toxicity due to the combination of NPs and mechanical pressure waves, in the present case SW.

A first analysis was performed to assess the extent of ROS generation contributing to this phenomenon. US stimulation has already been reported to induce a high ROS generation due to gas bubble cavitation trapped on the ZnO surface.^49^ However, to our knowledge, the effect of repeated SWs has never been analyzed in these terms. EPR spectroscopy was thus exploited: Fe:ZnO NPs were mechanically stimulated in water, and the amount of generated ROS was analyzed. The results of this measurements (Figure S3 in the Supporting Information) clearly indicate the absence of any signal related to the existence of hydroxyl and alkyl radicals, suggesting that none of these types of ROS are generated due to the SW stimulation, either alone or in combination with NPs. A possible explanation for this phenomenon might be that the excitation frequency of SW is not suitable to establish any cavitation phenomena. An alternative explanation can also be that the DMPO trap is not sensitive to the specific radicals formed in response to this stimulation. Therefore, ROS generation, if potentially present, may not be provoked by the combination of SW and NPs, and thus, it is excluded from being the main responsible for the observed cell death.

A second analysis was performed to evaluate the dissolution of the Fe:ZnO NPs inside cell culture medium, first in the absence of SW and then with their mechanical stimulation in the presence of living cells.

Preliminarily, the Zn ions dissolved in RPMI 1640 were measured at different times of incubation at 37 °C, in the absence of cells. The results (Figure S4 in the Supporting Information) suggest that the NP dissolution is almost immediate and approximately corresponding to 60%, with no relevant differences between the various incubation times. It is therefore assumed that an equilibrium is probably reached very soon in these conditions.

**Figure 7:**
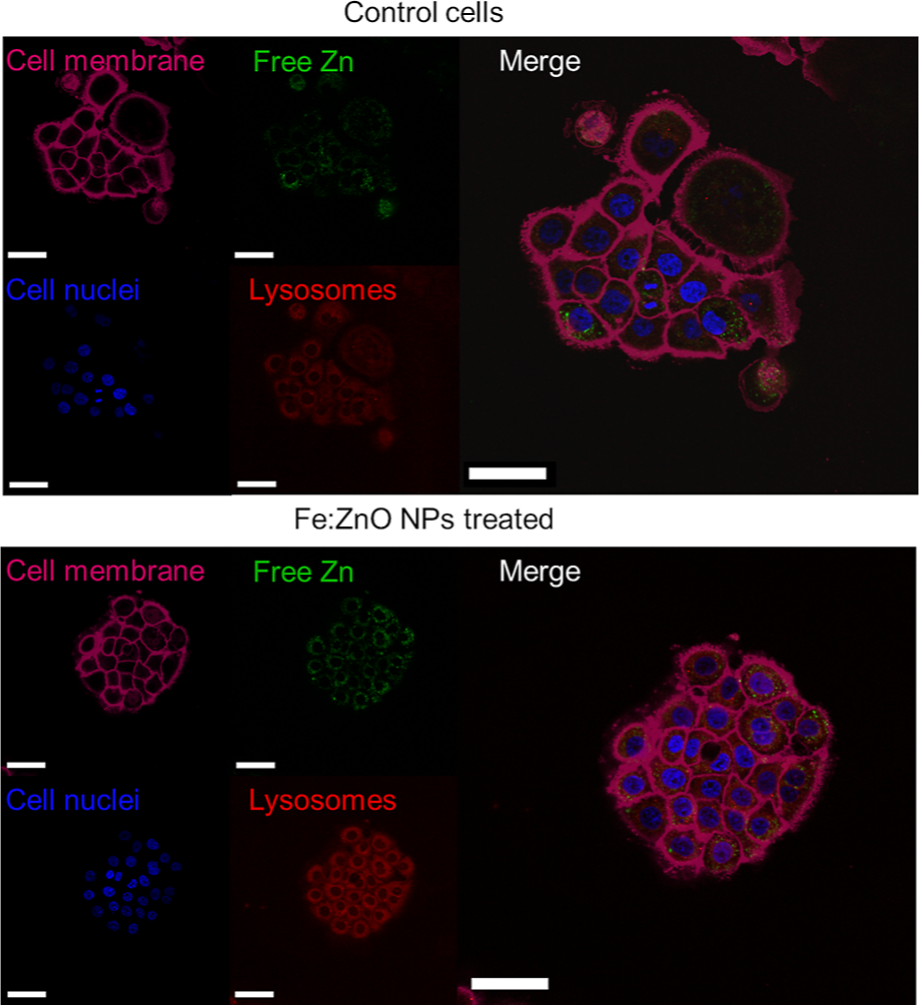
Representative fluorescence microscopy images of BxPC-3 cells treated with Fe:ZnO NPs and the control ones. In the images, membranes (purple), nuclei (blue), lysosomes (red), and free zinc inside the cells (green) are referred. The scale bar is 50 μm.

Fe:ZnO NP dissolution and the intracellular permeation of the resulting Zn^2+^ ions were also evaluated through fluorescence microscopy assays. In particular, a fluorescent dye was used to label free zinc inside the cells and another fluorescent dye was exploited to label lysosomes and to determine the location of the retained zinc inside the cell. Figure 7 shows a representative image of a control group of BxPC-3 cells without any NP treatment, in comparison with Fe:ZnO NP-treated cells. As it can be noticed, the control group already presents a fluorescence signal in the green channel, indicating that Zn^2+^ ions are physiologically present inside the cells. This is expected from previous literature evidence, being Zn^2+^ ions important in a series of physiological signaling pathways,^70−72^ and therefore, this fluorescence signal should be considered as a basal level.

When cells are treated with Fe:ZnO NPs, the obtained fluorescence signal corresponding to zinc ions is brighter than the control one. It is clear that Zn^2+^ ions are released by the NPs and permeate the cells, probably affecting the intracellular homeostasis equilibrium and, in the case of an excessive dose, eventually inducing cell death. Another aspect emerged from this analysis is the increase in the intensity of the signal related to the lysosomes. Indeed, when cells are treated with NPs, the lysosomes are present in a larger number than in the control cells. If the NPs are trapped in an acid environment as the one distinctive of lysosomes,^73−75^ they might dissolve because of the well-known instability of ZnO in a low pH solution.^56,76^ This phenomenon can explain the partial co-localization of the lysosome tracker with the zinc-related signal. After NP dissolution, it is very likely that Zn^2+^ ions diffuse throughout the cell, altering the cell homeostasis and to some extent explaining the partial absence of colocalization between the Zn^2+^-related signal and the lysosomes’ one.

**Figure 8:**
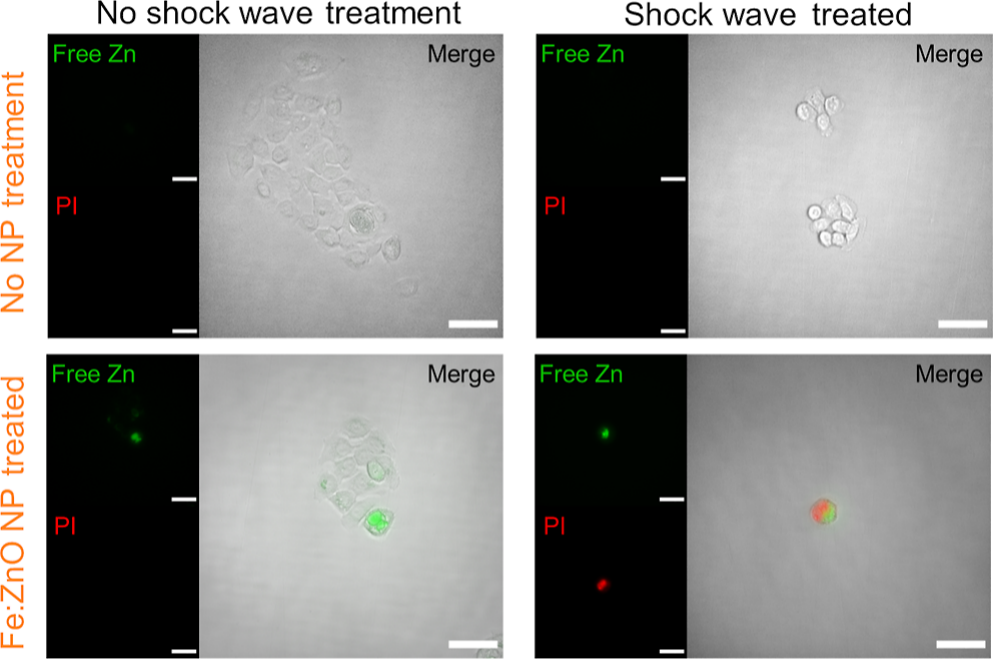
Representative microscopy images of BxPC-3 cells treated with SWs and Fe:ZnO NPs. The images highlight the phenomena occurring after the treatment with Fe:ZnO NPs and the SWs in terms of free zinc present in the cells (green) and membrane integrity (red, PI). Only when both the treatments are applied to cells, the two signals are clearly visible. The scale bar is 50 μm.

Further information about the cells’ fate after the synergistic treatments can also be obtained by staining cells with both the zinc ion probe and PI under fluorescence microscopy (Figure 8). PI is a cell impermeant dye, able to emit fluorescence when bound to the DNA. Therefore, it is detectable only when the cell membrane is compromised, allowing the dye to permeate it.

As expected, when no treatments are administered to cells, the signals related to both Zn^2+^ ions and cell membrane disruption are completely absent. Cells are well adherent to the substrate and viable. The treatment with Fe:ZnO NPs induces an increase in free Zn ions inside the cells, as already noticed in the previous microscopy analyses, but no changes in viability and in cell morphology are observed in this case. The PI signal is completely absent, indicating that the cell membrane is intact and that cells are not severely suffering for the treatment.

When only the SW are administered to the cells, PI and Zn ion probe signals are also absent. However, the morphology of cells seems slightly rounder than the one observed in the control samples. Anyway, the absence of PI signal indicates that the cell membrane is still intact, and therefore, cells could be still considered viable.

When both treatments are applied to the cells, the situation dramatically changes. First, it becomes very difficult to find a sufficient number of cells in the field of view. This is due to the pronounced cell death, which causes their removal during the washing steps performed during the staining procedure. The few remaining cells present both the PI and Zn ion-related signals. It is therefore possible to suppose that in this case, cells are suffering for the treatment. Probably, the Zn ions released by the Fe:ZnO NPs weaken the cell, which in turn becomes more susceptible to the mechanical stimulation. The final result is cell membrane disruption and the resulting cell detachment, which turns to cell death shortly afterward.

**Figure 9:**
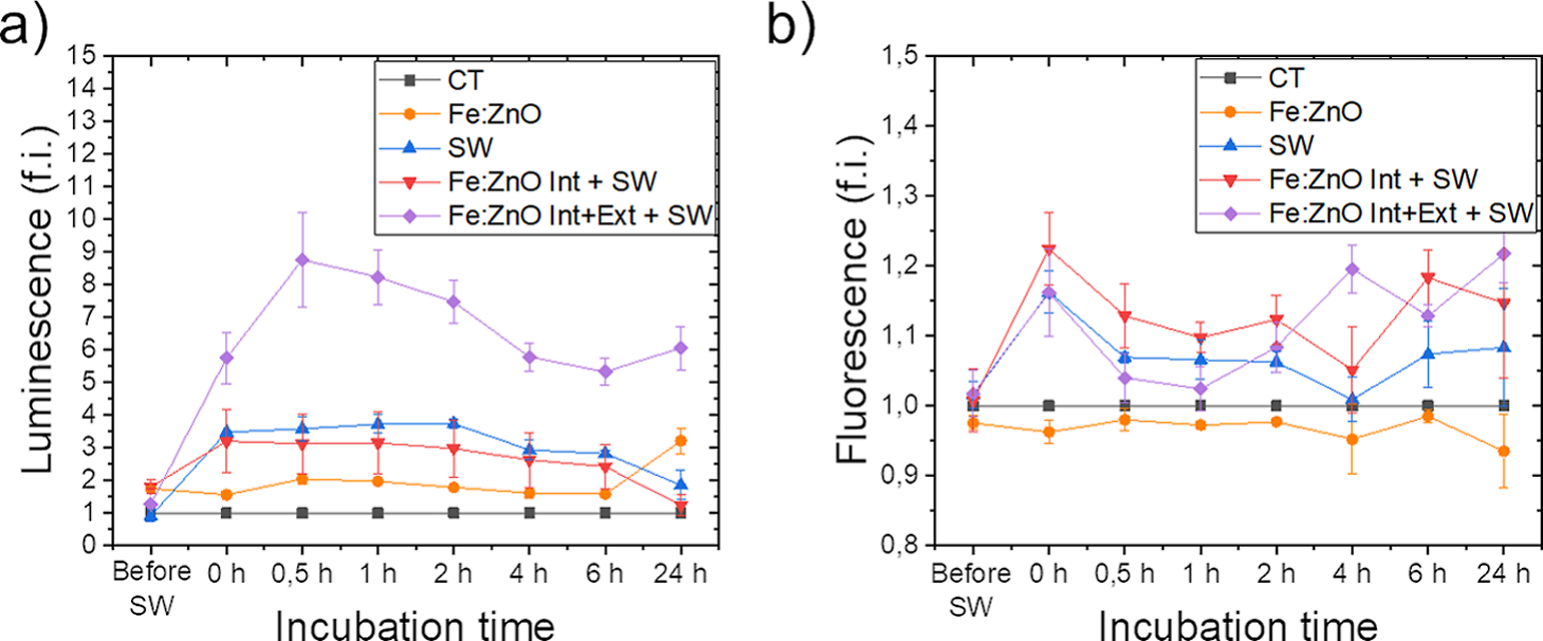
Cell death kinetics after SW treatment obtained through the apoptosis–necrosis assay. In panel (a), the luminescence is reported as fold induction (f.i.) of the control sample, while panel (b) reports the fluorescence as fold induction of the respective control. Cells belonging to the control sample (CT) were treated neither with Fe:ZnO NPs nor with SW. BxPC-3 cells were analyzed when incubated with Fe:ZnO NPs (Fe:ZnO sample) without any further treatment, without NPs but treated with SW (SW), treated with SW after a washing aimed at removing the non-internalized NPs that were administered 24 h before (Fe:ZnO Int + SW) and treated with SW after 24 h from Fe:ZnO NP administration.

A qualitative kinetic analysis of cell death was carried out throughout 24 h of incubation starting from the SW stimulation, evaluating two signals, that is, luminescence and fluorescence. The results are reported in Figure 9. The luminescence signal is related to the exposure of phosphatidylserine outside the cell membrane, which is reported in the literature to indicate apoptosis.^77,78^ Conversely, the fluoresce signal is associated with the secondary necrosis, being it based on a cell-impermeant profluorescent DNA dye, which can emit a fluorescence only when the cell membrane is broken.^50^ This assay allows us to determine the cell death mechanism by the detection of one, both, or none of the above-mentioned signals. In the case under analysis in Figure 9, the signals are all referred to the untreated cells’ one. As shown, the luminescence signals of cells treated with only Fe:ZnO NPs (orange curves in Figure 9) are slightly higher than the control one. This indicates that cells are not particularly suffering for the NP administration, being the signal almost constant all along the measurement time. However, the fluorescence signal is lower than the control for all the considered time steps, excluding the occurrence of necrosis during the incubation time. It is therefore fair to suppose that the cell death mechanism in response to the iron-doped ZnO NP administration is apoptosis. When cells are treated with SWs alone (blue curves in Figure 9), the situation is slightly different. Indeed, as soon as the SW treatment is completed, both fluorescence and luminescence signals increase with respect to the basal one. This means that there are cells that are going toward alternative forms of cell death, for example, necrosis, immediately during the treatment. Anyway, despite the luminescence signal remains constant during the 24 h of analysis, the fluorescence signal initially decreases and raises again only in the last hours of incubation. The reason of this behavior may be attributed to the occurrence of both apoptosis and necrosis at the beginning. Then, the early apoptotic cells undergo late apoptosis, with the consequent secondary increase of the fluorescence signal.

A similar behavior is found for the cells treated with both SW and internalized Fe:ZnO NPs (red curve), which display the increase of both luminescence and fluorescence signals with respect to the basal one. In particular, a slightly enhanced fluorescence signal is reported, qualitatively indicating a higher level of secondary necrosis.

More relevant differences can be found when cells are treated with both SW and Fe:ZnO NPs (both the extracellular and intracellular ones), as shown by the violet curves. Indeed, as in the case of SW alone, there is an increase of the luminescence signal just after the SW treatment. However, the increase is notably higher and continues over the first 30 min. The signal decreases over time but still indicates a considerable level of apoptotic events. The fluorescence signal is visible from the very beginning and does not underlie differences with respect to the cells treated with SW alone or with SW in the presence of internalized NPs.

In summary, these analyses allow us to hypothesize that a combination of both apoptosis and necrosis mechanisms is occurring in the first 24 h of NP incubation after the SW treatment.

Combining all the collected data, it can be assumed that cells die within the first 24 h due to the synergistic combination of SW and Fe:ZnO NPs. The most important outlined damages could be attributed to cell membrane rupture and ZnO dissolution into cytotoxic Zn cations, whose presence seems to lead to a conspicuous level of cell death.

## 4 Conclusions

In the present paper, we report on the use of biocompatible and biodegradable iron-doped ZnO NPs having bioimaging capabilities as magnetic NPs and a pronounced cytotoxic effect when combined with mechanical pressure waves, that is, SWs. The iron-doped NPs show superior features than the undoped ones, having better cytocompatibility and higher internalization rate than the pristine ZnO NPs. They were both tested in an *in vitro* model of PDAC and on the healthy counterpart. These features allow their use in a synergistic treatment with SW for an innovative stimuli-responsive therapy. From the cytotoxicity results and the related cell death mechanism investigations, the intracellular release of Zn^2+^ ions and the permeabilization of cell membrane are both considered responsible to induce cell death. In contrast, no ROS generation is observed. Ad hoc cell death analyses further allow us to point out the pronounced apoptotic and necrotic mechanisms of BxPC-3 cell death, once these cells are exposed to the combination of Fe:ZnO NPs and SWs for 24 h.

The obtained proof-of-concept results pave the way for a deep future study toward clinical translation. The advantages of the proposed synergistic activity rely on the use of potentially theranostic NPs having magnetic properties and of an on-demand therapeutic activity once activated by SWs. In addition, SW allows easy treatment of deep-seated tumor masses, such as pancreatic cancer, opening new perspectives in its therapeutic approach while monitoring the state of the disease. The NPs here presented still lack of a proper targeting system toward cancer cells despite a good selectivity in killing cancer cells rather than normal cells. Further improvements are therefore envisioned to render the proposed NPs even more biomimetic with an organic coating and able to actively target the diseased tissue and its microenvironment, with the final aim of overcoming the high interstitial pressure of this specific tumor mass.

## Supporting Information Available

Chemical characterization of ZnO NPs, magnetic properties of Fe:ZnO and ZnO NPs, generation of ROS in water after SW stimulation, and evaluation of the dissolution of Fe:ZnO NPs in cell culture medium (PDF)
