# Supplementary material for: Synergistic Phenomena between Iron-Doped ZnO Nanoparticles and Shock Waves Exploited against Pancreatic Cancer Cells
Source: ACS Appl Nano Mater. 2022 Nov 2;5(11):17212–25. doi: 10.1021/acsanm.2c04211 (PMC9953328; doi:10.1021/acsanm.2c04211)
Supplement: Supplementary file 1 — an2c04211_si_001.pdf [file an2c04211_si_001.pdf]

Supporting information

Synergistic ~~P~~phenomena between ~~iron~~Iron-~~doped~~ Doped ZnO ~~nanoparticles~~ Nanoparticles and ~~shock~~ Shock ~~waves~~ Waves ~~exploited~~ Exploited against ~~pancreatic~~ Pancreatic ~~cancer~~ Cancer ~~cells~~Cells

Marco Carofiglio, Marzia Conte, Luisa Racca, Valentina Cauda**

Corresponding authors* e-mails: luisa.racca@polito.it; valentina.cauda@polito.it

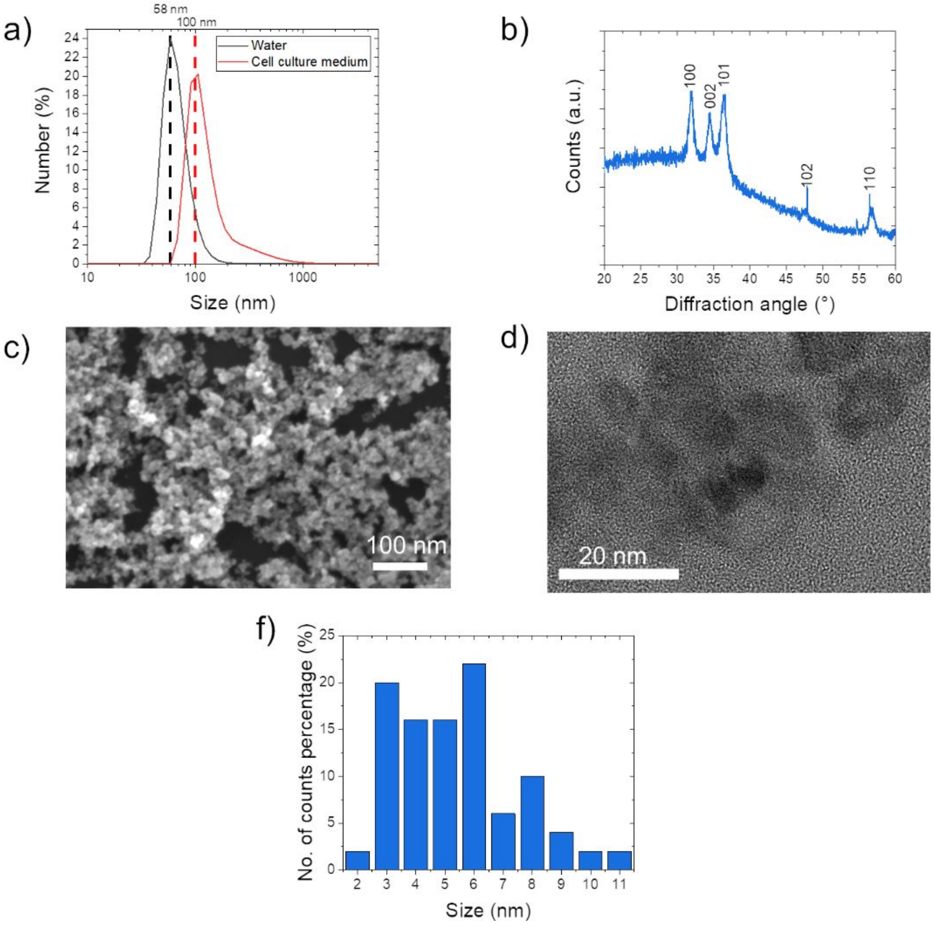

Figure S1. Characterization of undoped ZnO NPs: a) hydrodynamic diameter distribution in bidistilled water and cell culture medium obtained by DLS measurements; b) XRD pattern; c) representative FESEM image d) representative TEM image and f) histogram of TEM size of ZnO NPs.

Table S1. EDS measurements for ZnO and Fe:ZnO NPs

	Zn (atomic %)	Fe (atomic %)	O (atomic %)
ZnO NPs	18.65	-	81.35
Fe:ZnO NPs	23.68	1.20	75.12

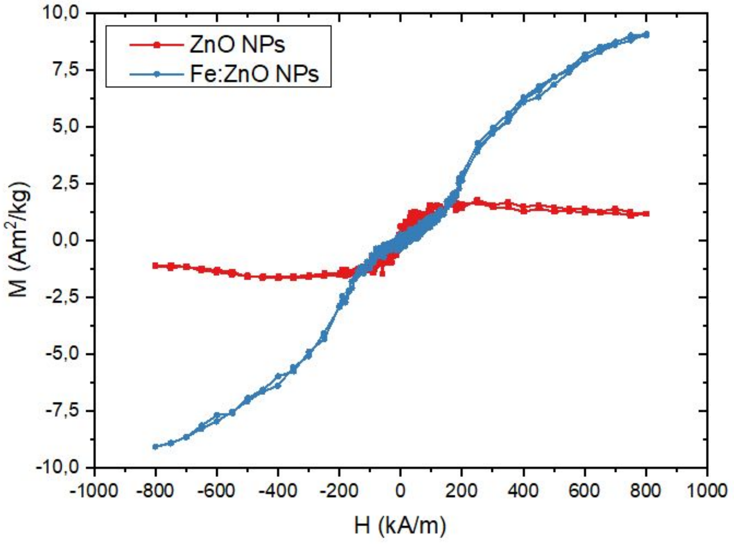

Figure S2. DC magnetization measurements of ZnO and Fe:ZnO NPs. The graph shows the magnetization of 1 mg of NPs embedded in Durcupan ACM resin (Sigma-Aldrich) measured by

means of a DC magnetometer (Lake Shore 7225, Lake Shore Cryotronics) at room temperature and in quasistatic conditions.

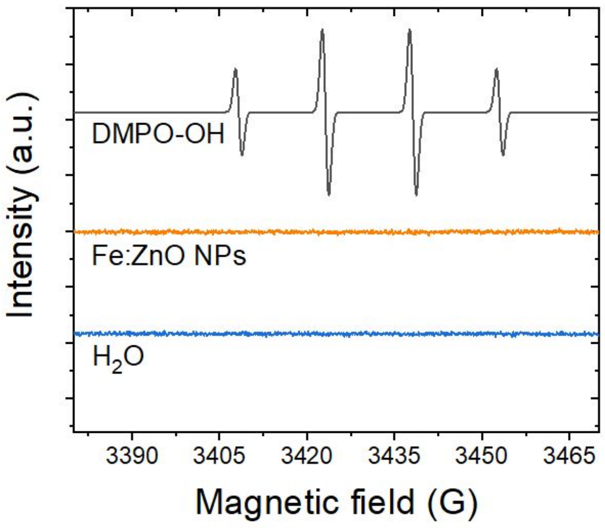

Figure S3. Electron paramagnetic resonance (EPR) spectroscopy analysis of Fe:ZnO NPs dispersed in water and stimulated with SW. The typical spin-adduct of DMPO-OH (an example is reported on the top of the graph) has ~~not~~ been detected nneither in pure water nor in presence of Fe:ZnO NPs (15 µg/mL) after the SW stimulation.

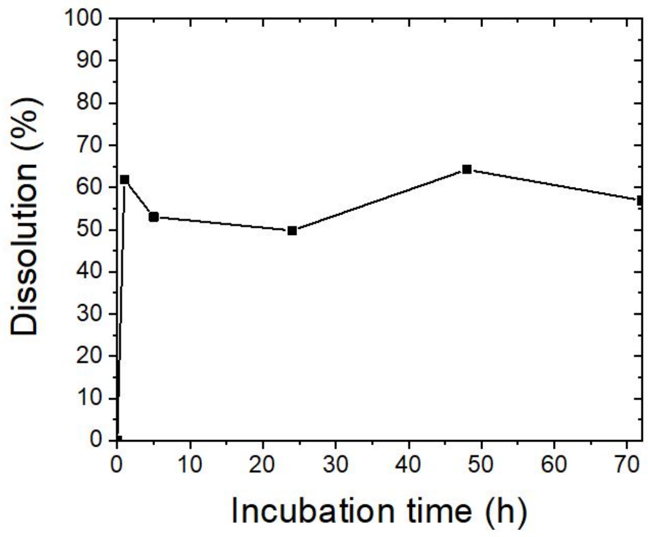

Figure S4. Fe:ZnO NPs dissolution in cell culture medium at 37°C. A partial dissolution occurs in the first moments of NPs dispersion in cell culture medium and then stabilizes in the first hours of incubation.

Statistical analysis for ZnO and Fe:ZnO cytotoxicity on BxPC-3 cell line. Bonferroni t-test (Three Ways ANOVA)								
**NPs**								
	MeanDiff	SEM	t Value	Prob	Alpha	Sig	LCL	UCL
Fe:ZnO ZnO	20,95919	0,99162	21,1364	2,30E-28	0,05	1	18,97274	22,94563
**Dose**								
	MeanDiff	SEM	t Value	Prob	Alpha	Sig	LCL	UCL
10 µg/mL 15 µg/mL	42,53285	1,40236	30,32956	1,15E-35	0,05	1	38,69709	46,3686
10 µg/mL 20 µg/mL	94,24194	1,40236	67,20257	1,87E-54	0,05	1	90,40619	98,0777
10 µg/mL 25 µg/mL	100,5981	1,40236	71,73507	5,04E-56	0,05	1	96,76237	104,4339
15 µg/mL 20 µg/mL	51,70909	1,40236	36,87301	3,43E-40	0,05	1	47,87334	55,54485
15 µg/mL 25 µg/mL	58,06527	1,40236	41,40551	6,51E-43	0,05	1	54,22952	61,90103
20 µg/mL 25 µg/mL	6,35618	1,40236	4,5325	1,87E-04	0,05	1	2,52042	10,19193
**Incubation time**								

	MeanDiff	SEM	t Value	Prob	Alpha	Sig	LCL	UCL
24 h 48 h	0,27608	1,18655	0,23268	1	0,05	0	-2,65236	3,20452
24 h 72 h	3,00335	1,18655	2,53116	0,04261	0,05	1	0,07491	5,93179
48 h 72 h	2,72727	1,26848	2,15003	0,10766	0,05	0	-0,40337	5,8579

Interactions

NPs	Dose	Incubation time	NPs	Dose	Incubation time	MeanDiff	SEM	t Value	Prob	Alpha	Sig	LCL	UCL
Fe:ZnO	10 µg/mL	--	Fe:ZnO	15 µg/mL	--	1,9596	1,98323	0,98808	1	0,05	0	-4,54653	8,46573
Fe:ZnO	10 µg/mL	--	Fe:ZnO	20 µg/mL	--	82,33599	1,98323	41,51608	2,63E-42	0,05	1	75,82986	88,84212
Fe:ZnO	10 µg/mL	--	Fe:ZnO	25 µg/mL	--	93,82453	1,98323	47,30892	2,14E-45	0,05	1	87,3184	100,3307
Fe:ZnO	10 µg/mL	--	ZnO	10 µg/mL	--	-8,66721	1,98323	-4,37025	0,00152	0,05	1	-15,1733	-2,16108
Fe:ZnO	10 µg/mL	--	ZnO	15 µg/mL	--	74,43888	1,98323	37,53414	6,15E-40	0,05	1	67,93276	80,94501
Fe:ZnO	10 µg/mL	--	ZnO	20 µg/mL	--	97,48068	1,98323	49,15246	2,64E-46	0,05	1	90,97456	103,9868
Fe:ZnO	10 µg/mL	--	ZnO	25 µg/mL	--	98,7045	1,98323	49,76954	1,33E-46	0,05	1	92,19837	105,2106
Fe:ZnO	15 µg/mL	--	Fe:ZnO	20 µg/mL	--	80,37639	1,98323	40,528	9,71E-42	0,05	1	73,87026	86,88252
Fe:ZnO	15 µg/mL	--	Fe:ZnO	25 µg/mL	--	91,86493	1,98323	46,32084	6,77E-45	0,05	1	85,3588	98,37105
Fe:ZnO	15 µg/mL	--	ZnO	10 µg/mL	--	-10,6268	1,98323	-5,35833	4,58E-05	0,05	1	-17,1329	-4,12068
Fe:ZnO	15 µg/mL	--	ZnO	15 µg/mL	--	72,47928	1,98323	36,54606	2,58E-39	0,05	1	65,97315	78,98541
Fe:ZnO	15 µg/mL	--	ZnO	20 µg/mL	--	95,52108	1,98323	48,16437	8,03E-46	0,05	1	89,01496	102,0272
Fe:ZnO	15 µg/mL	--	ZnO	25 µg/mL	--	96,7449	1,98323	48,78146	4,00E-46	0,05	1	90,23877	103,251
Fe:ZnO	20 µg/mL	--	Fe:ZnO	25 µg/mL	--	11,48854	1,98323	5,79284	9,23E-06	0,05	1	4,98241	17,99467
Fe:ZnO	20 µg/mL	--	ZnO	10 µg/mL	--	-91,0032	1,98323	-45,8863	1,13E-44	0,05	1	-97,5093	-84,4971
Fe:ZnO	20 µg/mL	--	ZnO	15 µg/mL	--	-7,89711	1,98323	-3,98194	0,00557	0,05	1	-14,4032	-1,39098
Fe:ZnO	20 µg/mL	--	ZnO	20 µg/mL	--	15,1447	1,98323	7,63638	8,61E-09	0,05	1	8,63857	21,65082
Fe:ZnO	20 µg/mL	--	ZnO	25 µg/mL	--	16,36851	1,98323	8,25346	8,30E-10	0,05	1	9,86238	22,87464
Fe:ZnO	25 µg/mL	--	ZnO	10 µg/mL	--	-102,492	1,98323	-51,6792	1,69E-47	0,05	1	-108,998	-95,9856
Fe:ZnO	25 µg/mL	--	ZnO	15 µg/mL	--	-19,3856	1,98323	-9,77478	2,90E-12	0,05	1	-25,8918	-12,8795
Fe:ZnO	25 µg/mL	--	ZnO	20 µg/mL	--	3,65616	1,98323	1,84354	1	0,05	0	-2,84997	10,16229
Fe:ZnO	25 µg/mL	--	ZnO	25 µg/mL	--	4,87997	1,98323	2,46062	0,47542	0,05	0	-1,62615	11,3861
ZnO	10 µg/mL	--	ZnO	15 µg/mL	--	83,10609	1,98323	41,90439	1,59E-42	0,05	1	76,59997	89,61222
ZnO	10 µg/mL	--	ZnO	20 µg/mL	--	106,1479	1,98323	53,52271	2,47E-48	0,05	1	99,64177	112,654
ZnO	10 µg/mL	--	ZnO	25 µg/mL	--	107,3717	1,98323	54,13979	1,32E-48	0,05	1	100,8656	113,8778
ZnO	15 µg/mL	--	ZnO	20 µg/mL	--	23,0418	1,98323	11,61831	4,23E-15	0,05	1	16,53567	29,54793
ZnO	15 µg/mL	--	ZnO	25 µg/mL	--	24,26562	1,98323	12,2354	5,22E-16	0,05	1	17,75949	30,77175
ZnO	20 µg/mL	--	ZnO	25 µg/mL	--	1,22382	1,98323	0,61708	1	0,05	0	-5,28231	7,72994
Fe:ZnO	--	24 h	Fe:ZnO	--	48 h	0,27982	1,67804	0,16675	1	0,05	0	-4,86563	5,42526
Fe:ZnO	--	24 h	Fe:ZnO	--	72 h	3,54508	1,67804	2,11263	0,58656	0,05	0	-1,60036	8,69052
Fe:ZnO	--	24 h	ZnO	--	24 h	21,32283	1,55356	13,72511	2,19E-18	0,05	1	16,55907	26,08658
Fe:ZnO	--	24 h	ZnO	--	48 h	21,59518	1,67804	12,86929	3,43E-17	0,05	1	16,44973	26,74062
Fe:ZnO	--	24 h	ZnO	--	72 h	23,78445	1,67804	14,17395	5,36E-19	0,05	1	18,63901	28,92989
Fe:ZnO	--	48 h	Fe:ZnO	--	72 h	3,26526	1,7939	1,8202	1	0,05	0	-2,23544	8,76597
Fe:ZnO	--	48 h	ZnO	--	24 h	21,04301	1,67804	12,54023	1,01E-16	0,05	1	15,89757	26,18845

Fe:ZnO	--	48 h	ZnO	--	48 h	21,31536	1,7939	11,88213	9,21E-16	0,05	1	15,81465	26,81607
Fe:ZnO	--	48 h	ZnO	--	72 h	23,50463	1,7939	13,10253	1,60E-17	0,05	1	18,00392	29,00534
Fe:ZnO	--	72 h	ZnO	--	24 h	17,77775	1,67804	10,59435	8,12E-14	0,05	1	12,6323	22,92319
Fe:ZnO	--	72 h	ZnO	--	48 h	18,0501	1,7939	10,06193	5,48E-13	0,05	1	12,54939	23,55081
Fe:ZnO	--	72 h	ZnO	--	72 h	20,23937	1,7939	11,28233	7,23E-15	0,05	1	14,73866	25,74008
ZnO	--	24 h	ZnO	--	48 h	0,27235	1,67804	0,1623	1	0,05	0	-4,87309	5,41779
ZnO	--	24 h	ZnO	--	72 h	2,46162	1,67804	1,46696	1	0,05	0	-2,68382	7,60707
ZnO	--	48 h	ZnO	--	72 h	2,18927	1,7939	1,2204	1	0,05	0	-3,31144	7,68998
--	10 µg/mL	24 h	--	10 µg/mL	48 h	0,85354	2,37311	0,35967	1	0,05	0	-7,60192	9,309
--	10 µg/mL	24 h	--	10 µg/mL	72 h	9,77402	2,37311	4,11866	0,00837	0,05	1	1,31856	18,22948
--	10 µg/mL	24 h	--	15 µg/mL	24 h	43,34478	2,19707	19,72845	4,60E-25	0,05	1	35,51654	51,17301
--	10 µg/mL	24 h	--	15 µg/mL	48 h	45,82817	2,37311	19,31147	1,31E-24	0,05	1	37,37271	54,28364
--	10 µg/mL	24 h	--	15 µg/mL	72 h	49,05314	2,37311	20,67043	4,61E-26	0,05	1	40,59768	57,5086
--	10 µg/mL	24 h	--	20 µg/mL	24 h	99,88108	2,19707	45,46104	4,44E-44	0,05	1	92,05284	107,7093
--	10 µg/mL	24 h	--	20 µg/mL	48 h	97,60284	2,37311	41,12872	1,03E-41	0,05	1	89,14737	106,0583
--	10 µg/mL	24 h	--	20 µg/mL	72 h	95,86947	2,37311	40,3983	2,72E-41	0,05	1	87,41401	104,3249
--	10 µg/mL	24 h	--	25 µg/mL	24 h	103,9446	2,19707	47,31053	5,03E-45	0,05	1	96,11631	111,7728
--	10 µg/mL	24 h	--	25 µg/mL	48 h	103,9902	2,37311	43,82028	3,29E-43	0,05	1	95,53473	112,4457
--	10 µg/mL	24 h	--	25 µg/mL	72 h	104,4872	2,37311	44,0297	2,54E-43	0,05	1	96,03172	112,9426
--	10 µg/mL	48 h	--	10 µg/mL	72 h	8,92048	2,53696	3,51621	0,05779	0,05	0	-0,11879	17,95975
--	10 µg/mL	48 h	--	15 µg/mL	24 h	42,49124	2,37311	17,90532	5,05E-23	0,05	1	34,03578	50,9467
--	10 µg/mL	48 h	--	15 µg/mL	48 h	44,97464	2,53696	17,72778	8,13E-23	0,05	1	35,93537	54,01391
--	10 µg/mL	48 h	--	15 µg/mL	72 h	48,1996	2,53696	18,99898	2,90E-24	0,05	1	39,16034	57,23887
--	10 µg/mL	48 h	--	20 µg/mL	24 h	99,02754	2,37311	41,72907	4,70E-42	0,05	1	90,57208	107,483
--	10 µg/mL	48 h	--	20 µg/mL	48 h	96,7493	2,53696	38,13595	6,14E-40	0,05	1	87,71003	105,7886
--	10 µg/mL	48 h	--	20 µg/mL	72 h	95,01593	2,53696	37,45271	1,63E-39	0,05	1	85,97666	104,0552
--	10 µg/mL	48 h	--	25 µg/mL	24 h	103,091	2,37311	43,44137	5,28E-43	0,05	1	94,63555	111,5465
--	10 µg/mL	48 h	--	25 µg/mL	48 h	103,1367	2,53696	40,65367	1,94E-41	0,05	1	94,09738	112,1759
--	10 µg/mL	48 h	--	25 µg/mL	72 h	103,6336	2,53696	40,84957	1,49E-41	0,05	1	94,59437	112,6729
--	10 µg/mL	72 h	--	15 µg/mL	24 h	33,57076	2,37311	14,14634	2,57E-18	0,05	1	25,1153	42,02622
--	10 µg/mL	72 h	--	15 µg/mL	48 h	36,05416	2,53696	14,21157	2,10E-18	0,05	1	27,01489	45,09343
--	10 µg/mL	72 h	--	15 µg/mL	72 h	39,27912	2,53696	15,48277	4,51E-20	0,05	1	30,23986	48,31839
--	10 µg/mL	72 h	--	20 µg/mL	24 h	90,10706	2,37311	37,97008	7,77E-40	0,05	1	81,6516	98,56252
--	10 µg/mL	72 h	--	20 µg/mL	48 h	87,82882	2,53696	34,61974	1,11E-37	0,05	1	78,78955	96,86809
--	10 µg/mL	72 h	--	20 µg/mL	72 h	86,09545	2,53696	33,93649	3,24E-37	0,05	1	77,05618	95,13472
--	10 µg/mL	72 h	--	25 µg/mL	24 h	94,17053	2,37311	39,68238	7,17E-41	0,05	1	85,71507	102,626
--	10 µg/mL	72 h	--	25 µg/mL	48 h	94,21617	2,53696	37,13746	2,57E-39	0,05	1	85,1769	103,2554
--	10 µg/mL	72 h	--	25 µg/mL	72 h	94,71316	2,53696	37,33336	1,93E-39	0,05	1	85,67389	103,7524
--	15 µg/mL	24 h	--	15 µg/mL	48 h	2,4834	2,37311	1,04647	1	0,05	0	-5,97207	10,93886
--	15 µg/mL	24 h	--	15 µg/mL	72 h	5,70836	2,37311	2,40544	1	0,05	0	-2,7471	14,16382
--	15 µg/mL	24 h	--	20 µg/mL	24 h	56,5363	2,19707	25,73259	6,88E-31	0,05	1	48,70806	64,36453

--	15 µg/mL	24 h	--	20 µg/mL	48 h	54,25806	2,37311	22,86372	2,93E-28	0,05	1	45,8026	62,71352
--	15 µg/mL	24 h	--	20 µg/mL	72 h	52,52469	2,37311	22,1333	1,51E-27	0,05	1	44,06923	60,98015
--	15 µg/mL	24 h	--	25 µg/mL	24 h	60,59977	2,19707	27,58208	1,87E-32	0,05	1	52,77153	68,428
--	15 µg/mL	24 h	--	25 µg/mL	48 h	60,64541	2,37311	25,55528	9,83E-31	0,05	1	52,18995	69,10087
--	15 µg/mL	24 h	--	25 µg/mL	72 h	61,1424	2,37311	25,76471	6,45E-31	0,05	1	52,68694	69,59786
--	15 µg/mL	48 h	--	15 µg/mL	72 h	3,22497	2,53696	1,27119	1	0,05	0	-5,8143	12,26424
--	15 µg/mL	48 h	--	20 µg/mL	24 h	54,0529	2,37311	22,77727	3,54E-28	0,05	1	45,59744	62,50836
--	15 µg/mL	48 h	--	20 µg/mL	48 h	51,77466	2,53696	20,40817	8,67E-26	0,05	1	42,73539	60,81393
--	15 µg/mL	48 h	--	20 µg/mL	72 h	50,04129	2,53696	19,72492	4,64E-25	0,05	1	41,00203	59,08056
--	15 µg/mL	48 h	--	25 µg/mL	24 h	58,11637	2,37311	24,48957	8,81E-30	0,05	1	49,66091	66,57183
--	15 µg/mL	48 h	--	25 µg/mL	48 h	58,16201	2,53696	22,92589	2,55E-28	0,05	1	49,12274	67,20128
--	15 µg/mL	48 h	--	25 µg/mL	72 h	58,65901	2,53696	23,12179	1,66E-28	0,05	1	49,61974	67,69827
--	15 µg/mL	72 h	--	20 µg/mL	24 h	50,82793	2,37311	21,41831	7,84E-27	0,05	1	42,37247	59,2834
--	15 µg/mL	72 h	--	20 µg/mL	48 h	48,54969	2,53696	19,13697	2,04E-24	0,05	1	39,51043	57,58896
--	15 µg/mL	72 h	--	20 µg/mL	72 h	46,81633	2,53696	18,45373	1,19E-23	0,05	1	37,77706	55,8556
--	15 µg/mL	72 h	--	25 µg/mL	24 h	54,8914	2,37311	23,13061	1,62E-28	0,05	1	46,43594	63,34687
--	15 µg/mL	72 h	--	25 µg/mL	48 h	54,93704	2,53696	21,65469	4,52E-27	0,05	1	45,89778	63,97631
--	15 µg/mL	72 h	--	25 µg/mL	72 h	55,43404	2,53696	21,8506	2,88E-27	0,05	1	46,39477	64,47331
--	20 µg/mL	24 h	--	20 µg/mL	48 h	-2,27824	2,37311	-0,96002	1	0,05	0	-10,7337	6,17722
--	20 µg/mL	24 h	--	20 µg/mL	72 h	-4,01161	2,37311	-1,69045	1	0,05	0	-12,4671	4,44385
--	20 µg/mL	24 h	--	25 µg/mL	24 h	4,06347	2,19707	1,8495	1	0,05	0	-3,76477	11,89171
--	20 µg/mL	24 h	--	25 µg/mL	48 h	4,10911	2,37311	1,73153	1	0,05	0	-4,34635	12,56457
--	20 µg/mL	24 h	--	25 µg/mL	72 h	4,6061	2,37311	1,94096	1	0,05	0	-3,84936	13,06157
--	20 µg/mL	48 h	--	20 µg/mL	72 h	-1,73337	2,53696	-0,68325	1	0,05	0	-10,7726	7,3059
--	20 µg/mL	48 h	--	25 µg/mL	24 h	6,34171	2,37311	2,67232	0,64997	0,05	0	-2,11375	14,79717
--	20 µg/mL	48 h	--	25 µg/mL	48 h	6,38735	2,53696	2,51772	0,97012	0,05	0	-2,65192	15,42662
--	20 µg/mL	48 h	--	25 µg/mL	72 h	6,88434	2,53696	2,71362	0,58262	0,05	0	-2,15492	15,92361
--	20 µg/mL	72 h	--	25 µg/mL	24 h	8,07508	2,37311	3,40274	0,08171	0,05	0	-0,38038	16,53054
--	20 µg/mL	72 h	--	25 µg/mL	48 h	8,12072	2,53696	3,20097	0,14903	0,05	0	-0,91855	17,15999
--	20 µg/mL	72 h	--	25 µg/mL	72 h	8,61771	2,53696	3,39687	0,08318	0,05	0	-0,42156	17,65698
--	25 µg/mL	24 h	--	25 µg/mL	48 h	0,04564	2,37311	0,01923	1	0,05	0	-8,40982	8,5011
--	25 µg/mL	24 h	--	25 µg/mL	72 h	0,54263	2,37311	0,22866	1	0,05	0	-7,91283	8,9981
--	25 µg/mL	48 h	--	25 µg/mL	72 h	0,49699	2,53696	0,1959	1	0,05	0	-8,54227	9,53626
Fe:ZnO	10 µg/mL	24 h	Fe:ZnO	10 µg/mL	48 h	-5,64589	3,35608	-1,68229	1	0,05	0	-19,106	7,81426
Fe:ZnO	10 µg/mL	24 h	Fe:ZnO	10 µg/mL	72 h	-0,37587	3,35608	-0,112	1	0,05	0	-13,836	13,08428
Fe:ZnO	10 µg/mL	24 h	Fe:ZnO	15 µg/mL	24 h	-12,137	3,10713	-3,90617	0,07034	0,05	0	-24,5987	0,32471
Fe:ZnO	10 µg/mL	24 h	Fe:ZnO	15 µg/mL	48 h	0,57997	3,35608	0,17281	1	0,05	0	-12,8802	14,04012
Fe:ZnO	10 µg/mL	24 h	Fe:ZnO	15 µg/mL	72 h	11,41404	3,35608	3,401	0,34352	0,05	0	-2,04611	24,87419
Fe:ZnO	10 µg/mL	24 h	Fe:ZnO	20 µg/mL	24 h	84,92562	3,10713	27,33253	1,25E-31	0,05	1	72,46395	97,3873
Fe:ZnO	10 µg/mL	24 h	Fe:ZnO	20 µg/mL	48 h	79,74475	3,35608	23,76128	1,73E-28	0,05	1	66,2846	93,2049
Fe:ZnO	10 µg/mL	24 h	Fe:ZnO	20 µg/mL	72 h	76,31584	3,35608	22,73958	1,61E-27	0,05	1	62,85569	89,77599

Fe:ZnO	10 µg/mL	24 h	Fe:ZnO	25 µg/mL	24 h	92,20259	3,10713	29,67456	1,68E-33	0,05	1	79,74092	104,6643
Fe:ZnO	10 µg/mL	24 h	Fe:ZnO	25 µg/mL	48 h	91,43168	3,35608	27,2436	1,49E-31	0,05	1	77,97153	104,8918
Fe:ZnO	10 µg/mL	24 h	Fe:ZnO	25 µg/mL	72 h	91,81755	3,35608	27,35857	1,19E-31	0,05	1	78,3574	105,2777
Fe:ZnO	10 µg/mL	24 h	ZnO	10 µg/mL	24 h	-19,7668	3,10713	-6,36175	1,08E-05	0,05	1	-32,2284	-7,30507
Fe:ZnO	10 µg/mL	24 h	ZnO	10 µg/mL	48 h	-12,4138	3,35608	-3,6989	0,13664	0,05	0	-25,8739	1,04636
Fe:ZnO	10 µg/mL	24 h	ZnO	10 µg/mL	72 h	0,15715	3,35608	0,04683	1	0,05	0	-13,303	13,6173
Fe:ZnO	10 µg/mL	24 h	ZnO	15 µg/mL	24 h	79,05978	3,10713	25,44466	5,14E-30	0,05	1	66,5981	91,52146
Fe:ZnO	10 µg/mL	24 h	ZnO	15 µg/mL	48 h	71,30962	3,35608	21,24789	4,89E-26	0,05	1	57,84948	84,76977
Fe:ZnO	10 µg/mL	24 h	ZnO	15 µg/mL	72 h	66,92549	3,35608	19,94157	1,14E-24	0,05	1	53,46534	80,38564
Fe:ZnO	10 µg/mL	24 h	ZnO	20 µg/mL	24 h	95,06978	3,10713	30,59734	3,34E-34	0,05	1	82,6081	107,5315
Fe:ZnO	10 µg/mL	24 h	ZnO	20 µg/mL	48 h	95,69417	3,35608	28,51368	1,37E-32	0,05	1	82,23402	109,1543
Fe:ZnO	10 µg/mL	24 h	ZnO	20 µg/mL	72 h	95,65635	3,35608	28,50241	1,40E-32	0,05	1	82,1962	109,1165
Fe:ZnO	10 µg/mL	24 h	ZnO	25 µg/mL	24 h	95,91975	3,10713	30,87089	2,08E-34	0,05	1	83,45807	108,3814
Fe:ZnO	10 µg/mL	24 h	ZnO	25 µg/mL	48 h	96,78195	3,35608	28,8378	7,57E-33	0,05	1	83,3218	110,2421
Fe:ZnO	10 µg/mL	24 h	ZnO	25 µg/mL	72 h	97,39005	3,35608	29,01899	5,45E-33	0,05	1	83,9299	110,8502
Fe:ZnO	10 µg/mL	48 h	Fe:ZnO	10 µg/mL	72 h	5,27001	3,5878	1,46887	1	0,05	0	-9,11949	19,65952
Fe:ZnO	10 µg/mL	48 h	Fe:ZnO	15 µg/mL	24 h	-6,49109	3,35608	-1,93413	1	0,05	0	-19,9512	6,96906
Fe:ZnO	10 µg/mL	48 h	Fe:ZnO	15 µg/mL	48 h	6,22586	3,5878	1,73529	1	0,05	0	-8,16365	20,61536
Fe:ZnO	10 µg/mL	48 h	Fe:ZnO	15 µg/mL	72 h	17,05993	3,5878	4,75498	0,00395	0,05	1	2,67042	31,44943
Fe:ZnO	10 µg/mL	48 h	Fe:ZnO	20 µg/mL	24 h	90,57151	3,35608	26,98729	2,43E-31	0,05	1	77,11136	104,0317
Fe:ZnO	10 µg/mL	48 h	Fe:ZnO	20 µg/mL	48 h	85,39063	3,5878	23,80028	1,59E-28	0,05	1	71,00113	99,78014
Fe:ZnO	10 µg/mL	48 h	Fe:ZnO	20 µg/mL	72 h	81,96172	3,5878	22,84456	1,28E-27	0,05	1	67,57222	96,35123
Fe:ZnO	10 µg/mL	48 h	Fe:ZnO	25 µg/mL	24 h	97,84848	3,35608	29,15559	4,26E-33	0,05	1	84,38833	111,3086
Fe:ZnO	10 µg/mL	48 h	Fe:ZnO	25 µg/mL	48 h	97,07756	3,5878	27,05768	2,12E-31	0,05	1	82,68806	111,4671
Fe:ZnO	10 µg/mL	48 h	Fe:ZnO	25 µg/mL	72 h	97,46344	3,5878	27,16524	1,73E-31	0,05	1	83,07393	111,853
Fe:ZnO	10 µg/mL	48 h	ZnO	10 µg/mL	24 h	-14,1209	3,35608	-4,20755	0,026	0,05	1	-27,581	-0,66072
Fe:ZnO	10 µg/mL	48 h	ZnO	10 µg/mL	48 h	-6,76791	3,5878	-1,88637	1	0,05	0	-21,1574	7,6216
Fe:ZnO	10 µg/mL	48 h	ZnO	10 µg/mL	72 h	5,80304	3,5878	1,61744	1	0,05	0	-8,58647	20,19254
Fe:ZnO	10 µg/mL	48 h	ZnO	15 µg/mL	24 h	84,70566	3,35608	25,23947	7,81E-30	0,05	1	71,24551	98,16581
Fe:ZnO	10 µg/mL	48 h	ZnO	15 µg/mL	48 h	76,95551	3,5878	21,44922	3,05E-26	0,05	1	62,566	91,34502
Fe:ZnO	10 µg/mL	48 h	ZnO	15 µg/mL	72 h	72,57137	3,5878	20,22726	5,63E-25	0,05	1	58,18187	86,96088
Fe:ZnO	10 µg/mL	48 h	ZnO	20 µg/mL	24 h	100,7157	3,35608	30,00991	9,30E-34	0,05	1	87,25551	114,1758
Fe:ZnO	10 µg/mL	48 h	ZnO	20 µg/mL	48 h	101,3401	3,5878	28,24574	2,25E-32	0,05	1	86,95055	115,7296
Fe:ZnO	10 µg/mL	48 h	ZnO	20 µg/mL	72 h	101,3022	3,5878	28,23519	2,29E-32	0,05	1	86,91273	115,6917
Fe:ZnO	10 µg/mL	48 h	ZnO	25 µg/mL	24 h	101,5656	3,35608	30,26318	5,96E-34	0,05	1	88,10548	115,0258
Fe:ZnO	10 µg/mL	48 h	ZnO	25 µg/mL	48 h	102,4278	3,5878	28,54892	1,28E-32	0,05	1	88,03833	116,8173
Fe:ZnO	10 µg/mL	48 h	ZnO	25 µg/mL	72 h	103,0359	3,5878	28,71842	9,42E-33	0,05	1	88,64643	117,4254
Fe:ZnO	10 µg/mL	72 h	Fe:ZnO	15 µg/mL	24 h	-11,7611	3,35608	-3,50442	0,25058	0,05	0	-25,2213	1,69905
Fe:ZnO	10 µg/mL	72 h	Fe:ZnO	15 µg/mL	48 h	0,95584	3,5878	0,26642	1	0,05	0	-13,4337	15,34535
Fe:ZnO	10 µg/mL	72 h	Fe:ZnO	15 µg/mL	72 h	11,78991	3,5878	3,28611	0,48481	0,05	0	-2,59959	26,17942
Fe:ZnO	10 µg/mL	72 h	Fe:ZnO	20 µg/mL	24 h	85,30149	3,35608	25,41701	5,44E-30	0,05	1	71,84134	98,76164

Fe:ZnO	10 µg/mL	72 h	Fe:ZnO	20 µg/mL	48 h	80,12062	3,5878	22,33141	4,03E-27	0,05	1	65,73111	94,51012
Fe:ZnO	10 µg/mL	72 h	Fe:ZnO	20 µg/mL	72 h	76,69171	3,5878	21,37569	3,62E-26	0,05	1	62,30221	91,08122
Fe:ZnO	10 µg/mL	72 h	Fe:ZnO	25 µg/mL	24 h	92,57846	3,35608	27,5853	7,75E-32	0,05	1	79,11832	106,0386
Fe:ZnO	10 µg/mL	72 h	Fe:ZnO	25 µg/mL	48 h	91,80755	3,5878	25,58881	3,84E-30	0,05	1	77,41804	106,1971
Fe:ZnO	10 µg/mL	72 h	Fe:ZnO	25 µg/mL	72 h	92,19343	3,5878	25,69637	3,09E-30	0,05	1	77,80392	106,5829
Fe:ZnO	10 µg/mL	72 h	ZnO	10 µg/mL	24 h	-19,3909	3,35608	-5,77784	9,62E-05	0,05	1	-32,851	-5,93073
Fe:ZnO	10 µg/mL	72 h	ZnO	10 µg/mL	48 h	-12,0379	3,5878	-3,35524	0,39435	0,05	0	-26,4274	2,35159
Fe:ZnO	10 µg/mL	72 h	ZnO	10 µg/mL	72 h	0,53303	3,5878	0,14857	1	0,05	0	-13,8565	14,92253
Fe:ZnO	10 µg/mL	72 h	ZnO	15 µg/mL	24 h	79,43565	3,35608	23,66918	2,10E-28	0,05	1	65,9755	92,8958
Fe:ZnO	10 µg/mL	72 h	ZnO	15 µg/mL	48 h	71,6855	3,5878	19,98035	1,03E-24	0,05	1	57,29599	86,075
Fe:ZnO	10 µg/mL	72 h	ZnO	15 µg/mL	72 h	67,30136	3,5878	18,75839	2,25E-23	0,05	1	52,91185	81,69087
Fe:ZnO	10 µg/mL	72 h	ZnO	20 µg/mL	24 h	95,44565	3,35608	28,43963	1,57E-32	0,05	1	81,9855	108,9058
Fe:ZnO	10 µg/mL	72 h	ZnO	20 µg/mL	48 h	96,07005	3,5878	26,77687	3,65E-31	0,05	1	81,68054	110,4596
Fe:ZnO	10 µg/mL	72 h	ZnO	20 µg/mL	72 h	96,03222	3,5878	26,76632	3,73E-31	0,05	1	81,64271	110,4217
Fe:ZnO	10 µg/mL	72 h	ZnO	25 µg/mL	24 h	96,29562	3,35608	28,69289	9,87E-33	0,05	1	82,83547	109,7558
Fe:ZnO	10 µg/mL	72 h	ZnO	25 µg/mL	48 h	97,15782	3,5878	27,08005	2,03E-31	0,05	1	82,76831	111,5473
Fe:ZnO	10 µg/mL	72 h	ZnO	25 µg/mL	72 h	97,76592	3,5878	27,24955	1,47E-31	0,05	1	83,37642	112,1554
Fe:ZnO	15 µg/mL	24 h	Fe:ZnO	15 µg/mL	48 h	12,71694	3,35608	3,78923	0,10253	0,05	0	-0,74321	26,17709
Fe:ZnO	15 µg/mL	24 h	Fe:ZnO	15 µg/mL	72 h	23,55101	3,35608	7,01742	8,94E-07	0,05	1	10,09086	37,01116
Fe:ZnO	15 µg/mL	24 h	Fe:ZnO	20 µg/mL	24 h	97,06259	3,10713	31,23871	1,11E-34	0,05	1	84,60092	109,5243
Fe:ZnO	15 µg/mL	24 h	Fe:ZnO	20 µg/mL	48 h	91,88172	3,35608	27,37769	1,15E-31	0,05	1	78,42157	105,3419
Fe:ZnO	15 µg/mL	24 h	Fe:ZnO	20 µg/mL	72 h	88,45281	3,35608	26,35599	8,32E-31	0,05	1	74,99266	101,913
Fe:ZnO	15 µg/mL	24 h	Fe:ZnO	25 µg/mL	24 h	104,3396	3,10713	33,58073	2,38E-36	0,05	1	91,87789	116,8012
Fe:ZnO	15 µg/mL	24 h	Fe:ZnO	25 µg/mL	48 h	103,5687	3,35608	30,86001	2,12E-34	0,05	1	90,1085	117,0288
Fe:ZnO	15 µg/mL	24 h	Fe:ZnO	25 µg/mL	72 h	103,9545	3,35608	30,97499	1,74E-34	0,05	1	90,49438	117,4147
Fe:ZnO	15 µg/mL	24 h	ZnO	10 µg/mL	24 h	-7,62978	3,10713	-2,45557	1	0,05	0	-20,0915	4,8319
Fe:ZnO	15 µg/mL	24 h	ZnO	10 µg/mL	48 h	-0,27682	3,35608	-0,08248	1	0,05	0	-13,737	13,18333
Fe:ZnO	15 µg/mL	24 h	ZnO	10 µg/mL	72 h	12,29413	3,35608	3,66324	0,1529	0,05	0	-1,16602	25,75428
Fe:ZnO	15 µg/mL	24 h	ZnO	15 µg/mL	24 h	91,19675	3,10713	29,35084	3,00E-33	0,05	1	78,73507	103,6584
Fe:ZnO	15 µg/mL	24 h	ZnO	15 µg/mL	48 h	83,4466	3,35608	24,86431	1,69E-29	0,05	1	69,98645	96,90675
Fe:ZnO	15 µg/mL	24 h	ZnO	15 µg/mL	72 h	79,06246	3,35608	23,55798	2,68E-28	0,05	1	65,60231	92,52261
Fe:ZnO	15 µg/mL	24 h	ZnO	20 µg/mL	24 h	107,2068	3,10713	34,50351	5,58E-37	0,05	1	94,74507	119,6684
Fe:ZnO	15 µg/mL	24 h	ZnO	20 µg/mL	48 h	107,8311	3,35608	32,13009	2,50E-35	0,05	1	94,371	121,2913
Fe:ZnO	15 µg/mL	24 h	ZnO	20 µg/mL	72 h	107,7933	3,35608	32,11882	2,55E-35	0,05	1	94,33317	121,2535
Fe:ZnO	15 µg/mL	24 h	ZnO	25 µg/mL	24 h	108,0567	3,10713	34,77706	3,65E-37	0,05	1	95,59504	120,5184
Fe:ZnO	15 µg/mL	24 h	ZnO	25 µg/mL	48 h	108,9189	3,35608	32,45421	1,46E-35	0,05	1	95,45877	122,3791
Fe:ZnO	15 µg/mL	24 h	ZnO	25 µg/mL	72 h	109,527	3,35608	32,63541	1,09E-35	0,05	1	96,06687	122,9872
Fe:ZnO	15 µg/mL	48 h	Fe:ZnO	15 µg/mL	72 h	10,83407	3,5878	3,0197	1	0,05	0	-3,55543	25,22358
Fe:ZnO	15 µg/mL	48 h	Fe:ZnO	20 µg/mL	24 h	84,34565	3,35608	25,1322	9,73E-30	0,05	1	70,8855	97,8058
Fe:ZnO	15 µg/mL	48 h	Fe:ZnO	20 µg/mL	48 h	79,16477	3,5878	22,06499	7,37E-27	0,05	1	64,77527	93,55428
Fe:ZnO	15 µg/mL	48 h	Fe:ZnO	20 µg/mL	72 h	75,73587	3,5878	21,10928	6,77E-26	0,05	1	61,34636	90,12537

Fe:ZnO	15 µg/mL	48 h	Fe:ZnO	25 µg/mL	24 h	91,62262	3,35608	27,30049	1,33E-31	0,05	1	78,16247	105,0828
Fe:ZnO	15 µg/mL	48 h	Fe:ZnO	25 µg/mL	48 h	90,8517	3,5878	25,3224	6,59E-30	0,05	1	76,4622	105,2412
Fe:ZnO	15 µg/mL	48 h	Fe:ZnO	25 µg/mL	72 h	91,23758	3,5878	25,42995	5,30E-30	0,05	1	76,84808	105,6271
Fe:ZnO	15 µg/mL	48 h	ZnO	10 µg/mL	24 h	-20,3467	3,35608	-6,06265	3,32E-05	0,05	1	-33,8069	-6,88657
Fe:ZnO	15 µg/mL	48 h	ZnO	10 µg/mL	48 h	-12,9938	3,5878	-3,62165	0,1742	0,05	0	-27,3833	1,39574
Fe:ZnO	15 µg/mL	48 h	ZnO	10 µg/mL	72 h	-0,42282	3,5878	-0,11785	1	0,05	0	-14,8123	13,96669
Fe:ZnO	15 µg/mL	48 h	ZnO	15 µg/mL	24 h	78,47981	3,35608	23,38437	3,90E-28	0,05	1	65,01966	91,93996
Fe:ZnO	15 µg/mL	48 h	ZnO	15 µg/mL	48 h	70,72965	3,5878	19,71393	2,00E-24	0,05	1	56,34015	85,11916
Fe:ZnO	15 µg/mL	48 h	ZnO	15 µg/mL	72 h	66,34552	3,5878	18,49198	4,49E-23	0,05	1	51,95601	80,73502
Fe:ZnO	15 µg/mL	48 h	ZnO	20 µg/mL	24 h	94,4898	3,35608	28,15482	2,66E-32	0,05	1	81,02965	107,95
Fe:ZnO	15 µg/mL	48 h	ZnO	20 µg/mL	48 h	95,1142	3,5878	26,51045	6,14E-31	0,05	1	80,7247	109,5037
Fe:ZnO	15 µg/mL	48 h	ZnO	20 µg/mL	72 h	95,07637	3,5878	26,49991	6,27E-31	0,05	1	80,68687	109,4659
Fe:ZnO	15 µg/mL	48 h	ZnO	25 µg/mL	24 h	95,33977	3,35608	28,40808	1,67E-32	0,05	1	81,87962	108,7999
Fe:ZnO	15 µg/mL	48 h	ZnO	25 µg/mL	48 h	96,20197	3,5878	26,81364	3,40E-31	0,05	1	81,81247	110,5915
Fe:ZnO	15 µg/mL	48 h	ZnO	25 µg/mL	72 h	96,81008	3,5878	26,98313	2,45E-31	0,05	1	82,42058	111,1996
Fe:ZnO	15 µg/mL	72 h	Fe:ZnO	20 µg/mL	24 h	73,51158	3,35608	21,904	1,07E-26	0,05	1	60,05143	86,97173
Fe:ZnO	15 µg/mL	72 h	Fe:ZnO	20 µg/mL	48 h	68,3307	3,5878	19,04529	1,08E-23	0,05	1	53,9412	82,72021
Fe:ZnO	15 µg/mL	72 h	Fe:ZnO	20 µg/mL	72 h	64,9018	3,5878	18,08958	1,29E-22	0,05	1	50,51229	79,2913
Fe:ZnO	15 µg/mL	72 h	Fe:ZnO	25 µg/mL	24 h	80,78855	3,35608	24,0723	8,88E-29	0,05	1	67,3284	94,2487
Fe:ZnO	15 µg/mL	72 h	Fe:ZnO	25 µg/mL	48 h	80,01763	3,5878	22,3027	4,30E-27	0,05	1	65,62813	94,40714
Fe:ZnO	15 µg/mL	72 h	Fe:ZnO	25 µg/mL	72 h	80,40351	3,5878	22,41025	3,37E-27	0,05	1	66,01401	94,79302
Fe:ZnO	15 µg/mL	72 h	ZnO	10 µg/mL	24 h	-31,1808	3,35608	-9,29084	1,69E-10	0,05	1	-44,6409	-17,7206
Fe:ZnO	15 µg/mL	72 h	ZnO	10 µg/mL	48 h	-23,8278	3,5878	-6,64135	3,73E-06	0,05	1	-38,2173	-9,43833
Fe:ZnO	15 µg/mL	72 h	ZnO	10 µg/mL	72 h	-11,2569	3,5878	-3,13755	0,7496	0,05	0	-25,6464	3,13262
Fe:ZnO	15 µg/mL	72 h	ZnO	15 µg/mL	24 h	67,64574	3,35608	20,15618	6,70E-25	0,05	1	54,18559	81,10589
Fe:ZnO	15 µg/mL	72 h	ZnO	15 µg/mL	48 h	59,89558	3,5878	16,69424	5,81E-21	0,05	1	45,50608	74,28509
Fe:ZnO	15 µg/mL	72 h	ZnO	15 µg/mL	72 h	55,51145	3,5878	15,47228	1,94E-19	0,05	1	41,12194	69,90095
Fe:ZnO	15 µg/mL	72 h	ZnO	20 µg/mL	24 h	83,65573	3,35608	24,92662	1,48E-29	0,05	1	70,19558	97,11588
Fe:ZnO	15 µg/mL	72 h	ZnO	20 µg/mL	48 h	84,28013	3,5878	23,49075	3,10E-28	0,05	1	69,89063	98,66964
Fe:ZnO	15 µg/mL	72 h	ZnO	20 µg/mL	72 h	84,2423	3,5878	23,48021	3,17E-28	0,05	1	69,8528	98,63181
Fe:ZnO	15 µg/mL	72 h	ZnO	25 µg/mL	24 h	84,5057	3,35608	25,17989	8,82E-30	0,05	1	71,04555	97,96585
Fe:ZnO	15 µg/mL	72 h	ZnO	25 µg/mL	48 h	85,3679	3,5878	23,79394	1,61E-28	0,05	1	70,9784	99,75741
Fe:ZnO	15 µg/mL	72 h	ZnO	25 µg/mL	72 h	85,97601	3,5878	23,96343	1,12E-28	0,05	1	71,5865	100,3655
Fe:ZnO	20 µg/mL	24 h	Fe:ZnO	20 µg/mL	48 h	-5,18088	3,35608	-1,54373	1	0,05	0	-18,641	8,27927
Fe:ZnO	20 µg/mL	24 h	Fe:ZnO	20 µg/mL	72 h	-8,60978	3,35608	-2,56543	1	0,05	0	-22,0699	4,85037
Fe:ZnO	20 µg/mL	24 h	Fe:ZnO	25 µg/mL	24 h	7,27697	3,10713	2,34203	1	0,05	0	-5,18471	19,73865
Fe:ZnO	20 µg/mL	24 h	Fe:ZnO	25 µg/mL	48 h	6,50605	3,35608	1,93859	1	0,05	0	-6,9541	19,9662
Fe:ZnO	20 µg/mL	24 h	Fe:ZnO	25 µg/mL	72 h	6,89193	3,35608	2,05357	1	0,05	0	-6,56822	20,35208
Fe:ZnO	20 µg/mL	24 h	ZnO	10 µg/mL	24 h	-104,692	3,10713	-33,6943	1,98E-36	0,05	1	-117,154	-92,2307
Fe:ZnO	20 µg/mL	24 h	ZnO	10 µg/mL	48 h	-97,3394	3,35608	-29,0039	5,60E-33	0,05	1	-110,8	-83,8793
Fe:ZnO	20 µg/mL	24 h	ZnO	10 µg/mL	72 h	-84,7685	3,35608	-25,2582	7,52E-30	0,05	1	-98,2286	-71,3083

Fe:ZnO	20 µg/mL	24 h	ZnO	15 µg/mL	24 h	-5,86584	3,10713	-1,88787	1	0,05	0	-18,3275	6,59583
Fe:ZnO	20 µg/mL	24 h	ZnO	15 µg/mL	48 h	-13,616	3,35608	-4,05711	0,04291	0,05	1	-27,0762	-0,15585
Fe:ZnO	20 µg/mL	24 h	ZnO	15 µg/mL	72 h	-18,0001	3,35608	-5,36344	4,44E-04	0,05	1	-31,4603	-4,53998
Fe:ZnO	20 µg/mL	24 h	ZnO	20 µg/mL	24 h	10,14415	3,10713	3,2648	0,51644	0,05	0	-2,31752	22,60583
Fe:ZnO	20 µg/mL	24 h	ZnO	20 µg/mL	48 h	10,76855	3,35608	3,20867	0,6093	0,05	0	-2,6916	24,2287
Fe:ZnO	20 µg/mL	24 h	ZnO	20 µg/mL	72 h	10,73072	3,35608	3,1974	0,62975	0,05	0	-2,72943	24,19087
Fe:ZnO	20 µg/mL	24 h	ZnO	25 µg/mL	24 h	10,99412	3,10713	3,53836	0,22569	0,05	0	-1,46755	23,4558
Fe:ZnO	20 µg/mL	24 h	ZnO	25 µg/mL	48 h	11,85632	3,35608	3,53279	0,2296	0,05	0	-1,60383	25,31647
Fe:ZnO	20 µg/mL	24 h	ZnO	25 µg/mL	72 h	12,46443	3,35608	3,71399	0,13027	0,05	0	-0,99572	25,92458
Fe:ZnO	20 µg/mL	48 h	Fe:ZnO	20 µg/mL	72 h	-3,42891	3,5878	-0,95571	1	0,05	0	-17,8184	10,9606
Fe:ZnO	20 µg/mL	48 h	Fe:ZnO	25 µg/mL	24 h	12,45785	3,35608	3,71202	0,13108	0,05	0	-1,0023	25,918
Fe:ZnO	20 µg/mL	48 h	Fe:ZnO	25 µg/mL	48 h	11,68693	3,5878	3,25741	0,52786	0,05	0	-2,70258	26,07643
Fe:ZnO	20 µg/mL	48 h	Fe:ZnO	25 µg/mL	72 h	12,07281	3,5878	3,36496	0,38299	0,05	0	-2,3167	26,46231
Fe:ZnO	20 µg/mL	48 h	ZnO	10 µg/mL	24 h	-99,5115	3,35608	-29,6511	1,75E-33	0,05	1	-112,972	-86,0514
Fe:ZnO	20 µg/mL	48 h	ZnO	10 µg/mL	48 h	-92,1585	3,5878	-25,6866	3,15E-30	0,05	1	-106,548	-77,769
Fe:ZnO	20 µg/mL	48 h	ZnO	10 µg/mL	72 h	-79,5876	3,5878	-22,1828	5,64E-27	0,05	1	-93,9771	-65,1981
Fe:ZnO	20 µg/mL	48 h	ZnO	15 µg/mL	24 h	-0,68497	3,35608	-0,2041	1	0,05	0	-14,1451	12,77518
Fe:ZnO	20 µg/mL	48 h	ZnO	15 µg/mL	48 h	-8,43512	3,5878	-2,35106	1	0,05	0	-22,8246	5,95438
Fe:ZnO	20 µg/mL	48 h	ZnO	15 µg/mL	72 h	-12,8193	3,5878	-3,57301	0,20271	0,05	0	-27,2088	1,57025
Fe:ZnO	20 µg/mL	48 h	ZnO	20 µg/mL	24 h	15,32503	3,35608	4,56635	0,00764	0,05	1	1,86488	28,78518
Fe:ZnO	20 µg/mL	48 h	ZnO	20 µg/mL	48 h	15,94943	3,5878	4,44546	0,01159	0,05	1	1,55992	30,33893
Fe:ZnO	20 µg/mL	48 h	ZnO	20 µg/mL	72 h	15,9116	3,5878	4,43492	0,01202	0,05	1	1,5221	30,30111
Fe:ZnO	20 µg/mL	48 h	ZnO	25 µg/mL	24 h	16,175	3,35608	4,81961	0,00314	0,05	1	2,71485	29,63515
Fe:ZnO	20 µg/mL	48 h	ZnO	25 µg/mL	48 h	17,0372	3,5878	4,74865	0,00404	0,05	1	2,64769	31,42671
Fe:ZnO	20 µg/mL	48 h	ZnO	25 µg/mL	72 h	17,64531	3,5878	4,91814	0,00221	0,05	1	3,2558	32,03481
Fe:ZnO	20 µg/mL	72 h	Fe:ZnO	25 µg/mL	24 h	15,88675	3,35608	4,73372	0,00425	0,05	1	2,4266	29,3469
Fe:ZnO	20 µg/mL	72 h	Fe:ZnO	25 µg/mL	48 h	15,11584	3,5878	4,21312	0,02552	0,05	1	0,72633	29,50534
Fe:ZnO	20 µg/mL	72 h	Fe:ZnO	25 µg/mL	72 h	15,50172	3,5878	4,32067	0,01775	0,05	1	1,11221	29,89122
Fe:ZnO	20 µg/mL	72 h	ZnO	10 µg/mL	24 h	-96,0826	3,35608	-28,6294	1,11E-32	0,05	1	-109,543	-82,6224
Fe:ZnO	20 µg/mL	72 h	ZnO	10 µg/mL	48 h	-88,7296	3,5878	-24,7309	2,23E-29	0,05	1	-103,119	-74,3401
Fe:ZnO	20 µg/mL	72 h	ZnO	10 µg/mL	72 h	-76,1587	3,5878	-21,2271	5,13E-26	0,05	1	-90,5482	-61,7692
Fe:ZnO	20 µg/mL	72 h	ZnO	15 µg/mL	24 h	2,74394	3,35608	0,8176	1	0,05	0	-10,7162	16,20409
Fe:ZnO	20 µg/mL	72 h	ZnO	15 µg/mL	48 h	-5,00621	3,5878	-1,39534	1	0,05	0	-19,3957	9,38329
Fe:ZnO	20 µg/mL	72 h	ZnO	15 µg/mL	72 h	-9,39035	3,5878	-2,6173	1	0,05	0	-23,7799	4,99915
Fe:ZnO	20 µg/mL	72 h	ZnO	20 µg/mL	24 h	18,75394	3,35608	5,58805	1,94E-04	0,05	1	5,29379	32,21409
Fe:ZnO	20 µg/mL	72 h	ZnO	20 µg/mL	48 h	19,37833	3,5878	5,40117	3,86E-04	0,05	1	4,98883	33,76784
Fe:ZnO	20 µg/mL	72 h	ZnO	20 µg/mL	72 h	19,34051	3,5878	5,39063	4,02E-04	0,05	1	4,951	33,73001
Fe:ZnO	20 µg/mL	72 h	ZnO	25 µg/mL	24 h	19,60391	3,35608	5,84131	7,59E-05	0,05	1	6,14376	33,06406
Fe:ZnO	20 µg/mL	72 h	ZnO	25 µg/mL	48 h	20,46611	3,5878	5,70436	1,26E-04	0,05	1	6,0766	34,85561
Fe:ZnO	20 µg/mL	72 h	ZnO	25 µg/mL	72 h	21,07421	3,5878	5,87385	6,72E-05	0,05	1	6,68471	35,46372
Fe:ZnO	25 µg/mL	24 h	Fe:ZnO	25 µg/mL	48 h	-0,77092	3,35608	-0,22971	1	0,05	0	-14,2311	12,68923

Fe:ZnO	25 µg/mL	24 h	Fe:ZnO	25 µg/mL	72 h	-0,38504	3,35608	-0,11473	1	0,05	0	-13,8452	13,07511
Fe:ZnO	25 µg/mL	24 h	ZnO	10 µg/mL	24 h	-111,969	3,10713	-36,0363	5,42E-38	0,05	1	-124,431	-99,5077
Fe:ZnO	25 µg/mL	24 h	ZnO	10 µg/mL	48 h	-104,616	3,35608	-31,1722	1,25E-34	0,05	1	-118,077	-91,1562
Fe:ZnO	25 µg/mL	24 h	ZnO	10 µg/mL	72 h	-92,0454	3,35608	-27,4265	1,05E-31	0,05	1	-105,506	-78,5853
Fe:ZnO	25 µg/mL	24 h	ZnO	15 µg/mL	24 h	-13,1428	3,10713	-4,22989	0,02412	0,05	1	-25,6045	-0,68114
Fe:ZnO	25 µg/mL	24 h	ZnO	15 µg/mL	48 h	-20,893	3,35608	-6,22541	1,80E-05	0,05	1	-34,3531	-7,43282
Fe:ZnO	25 µg/mL	24 h	ZnO	15 µg/mL	72 h	-25,2771	3,35608	-7,53174	1,26E-07	0,05	1	-38,7373	-11,817
Fe:ZnO	25 µg/mL	24 h	ZnO	20 µg/mL	24 h	2,86718	3,10713	0,92278	1	0,05	0	-9,59449	15,32886
Fe:ZnO	25 µg/mL	24 h	ZnO	20 µg/mL	48 h	3,49158	3,35608	1,04037	1	0,05	0	-9,96857	16,95173
Fe:ZnO	25 µg/mL	24 h	ZnO	20 µg/mL	72 h	3,45375	3,35608	1,0291	1	0,05	0	-10,0064	16,9139
Fe:ZnO	25 µg/mL	24 h	ZnO	25 µg/mL	24 h	3,71715	3,10713	1,19633	1	0,05	0	-8,74452	16,17883
Fe:ZnO	25 µg/mL	24 h	ZnO	25 µg/mL	48 h	4,57935	3,35608	1,36449	1	0,05	0	-8,8808	18,0395
Fe:ZnO	25 µg/mL	24 h	ZnO	25 µg/mL	72 h	5,18746	3,35608	1,54569	1	0,05	0	-8,27269	18,64761
Fe:ZnO	25 µg/mL	48 h	Fe:ZnO	25 µg/mL	72 h	0,38588	3,5878	0,10755	1	0,05	0	-14,0036	14,77538
Fe:ZnO	25 µg/mL	48 h	ZnO	10 µg/mL	24 h	-111,198	3,35608	-33,1334	4,86E-36	0,05	1	-124,659	-97,7383
Fe:ZnO	25 µg/mL	48 h	ZnO	10 µg/mL	48 h	-103,845	3,5878	-28,9441	6,24E-33	0,05	1	-118,235	-89,456
Fe:ZnO	25 µg/mL	48 h	ZnO	10 µg/mL	72 h	-91,2745	3,5878	-25,4403	5,19E-30	0,05	1	-105,664	-76,885
Fe:ZnO	25 µg/mL	48 h	ZnO	15 µg/mL	24 h	-12,3719	3,35608	-3,68641	0,14214	0,05	0	-25,8321	1,08825
Fe:ZnO	25 µg/mL	48 h	ZnO	15 µg/mL	48 h	-20,1221	3,5878	-5,60846	1,80E-04	0,05	1	-34,5116	-5,73255
Fe:ZnO	25 µg/mL	48 h	ZnO	15 µg/mL	72 h	-24,5062	3,5878	-6,83042	1,82E-06	0,05	1	-38,8957	-10,1167
Fe:ZnO	25 µg/mL	48 h	ZnO	20 µg/mL	24 h	3,6381	3,35608	1,08403	1	0,05	0	-9,82205	17,09825
Fe:ZnO	25 µg/mL	48 h	ZnO	20 µg/mL	48 h	4,2625	3,5878	1,18805	1	0,05	0	-10,127	18,652
Fe:ZnO	25 µg/mL	48 h	ZnO	20 µg/mL	72 h	4,22467	3,5878	1,17751	1	0,05	0	-10,1648	18,61418
Fe:ZnO	25 µg/mL	48 h	ZnO	25 µg/mL	24 h	4,48807	3,35608	1,3373	1	0,05	0	-8,97208	17,94822
Fe:ZnO	25 µg/mL	48 h	ZnO	25 µg/mL	48 h	5,35027	3,5878	1,49124	1	0,05	0	-9,03923	19,73978
Fe:ZnO	25 µg/mL	48 h	ZnO	25 µg/mL	72 h	5,95838	3,5878	1,66073	1	0,05	0	-8,43113	20,34788
Fe:ZnO	25 µg/mL	72 h	ZnO	10 µg/mL	24 h	-111,584	3,35608	-33,2484	4,04E-36	0,05	1	-125,044	-98,1242
Fe:ZnO	25 µg/mL	72 h	ZnO	10 µg/mL	48 h	-104,231	3,5878	-29,0516	5,14E-33	0,05	1	-118,621	-89,8418
Fe:ZnO	25 µg/mL	72 h	ZnO	10 µg/mL	72 h	-91,6604	3,5878	-25,5478	4,17E-30	0,05	1	-106,05	-77,2709
Fe:ZnO	25 µg/mL	72 h	ZnO	15 µg/mL	24 h	-12,7578	3,35608	-3,80139	0,09861	0,05	0	-26,2179	0,70237
Fe:ZnO	25 µg/mL	72 h	ZnO	15 µg/mL	48 h	-20,5079	3,5878	-5,71602	1,21E-04	0,05	1	-34,8974	-6,11842
Fe:ZnO	25 µg/mL	72 h	ZnO	15 µg/mL	72 h	-24,8921	3,5878	-6,93797	1,21E-06	0,05	1	-39,2816	-10,5026
Fe:ZnO	25 µg/mL	72 h	ZnO	20 µg/mL	24 h	3,25222	3,35608	0,96905	1	0,05	0	-10,2079	16,71237
Fe:ZnO	25 µg/mL	72 h	ZnO	20 µg/mL	48 h	3,87662	3,5878	1,0805	1	0,05	0	-10,5129	18,26612
Fe:ZnO	25 µg/mL	72 h	ZnO	20 µg/mL	72 h	3,83879	3,5878	1,06996	1	0,05	0	-10,5507	18,2283
Fe:ZnO	25 µg/mL	72 h	ZnO	25 µg/mL	24 h	4,10219	3,35608	1,22232	1	0,05	0	-9,35796	17,56234
Fe:ZnO	25 µg/mL	72 h	ZnO	25 µg/mL	48 h	4,96439	3,5878	1,38369	1	0,05	0	-9,42511	19,3539
Fe:ZnO	25 µg/mL	72 h	ZnO	25 µg/mL	72 h	5,5725	3,5878	1,55318	1	0,05	0	-8,81701	19,962
ZnO	10 µg/mL	24 h	ZnO	10 µg/mL	48 h	7,35296	3,35608	2,19094	1	0,05	0	-6,10719	20,81311
ZnO	10 µg/mL	24 h	ZnO	10 µg/mL	72 h	19,92391	3,35608	5,93666	5,32E-05	0,05	1	6,46376	33,38406
ZnO	10 µg/mL	24 h	ZnO	15 µg/mL	24 h	98,82653	3,10713	31,80641	4,28E-35	0,05	1	86,36485	111,2882

ZnO	10 µg/mL	24 h	ZnO	15 µg/mL	48 h	91,07638	3,35608	27,13773	1,82E-31	0,05	1	77,61623	104,5365
ZnO	10 µg/mL	24 h	ZnO	15 µg/mL	72 h	86,69224	3,35608	25,8314	2,36E-30	0,05	1	73,23209	100,1524
ZnO	10 µg/mL	24 h	ZnO	20 µg/mL	24 h	114,8365	3,10713	36,95908	1,39E-38	0,05	1	102,3749	127,2982
ZnO	10 µg/mL	24 h	ZnO	20 µg/mL	48 h	115,4609	3,35608	34,40351	6,51E-37	0,05	1	102,0008	128,9211
ZnO	10 µg/mL	24 h	ZnO	20 µg/mL	72 h	115,4231	3,35608	34,39224	6,63E-37	0,05	1	101,963	128,8833
ZnO	10 µg/mL	24 h	ZnO	25 µg/mL	24 h	115,6865	3,10713	37,23264	9,35E-39	0,05	1	103,2248	128,1482
ZnO	10 µg/mL	24 h	ZnO	25 µg/mL	48 h	116,5487	3,35608	34,72763	3,94E-37	0,05	1	103,0886	130,0089
ZnO	10 µg/mL	24 h	ZnO	25 µg/mL	72 h	117,1568	3,35608	34,90883	2,98E-37	0,05	1	103,6967	130,617
ZnO	10 µg/mL	48 h	ZnO	10 µg/mL	72 h	12,57095	3,5878	3,5038	0,25105	0,05	0	-1,81856	26,96045
ZnO	10 µg/mL	48 h	ZnO	15 µg/mL	24 h	91,47357	3,35608	27,25608	1,45E-31	0,05	1	78,01342	104,9337
ZnO	10 µg/mL	48 h	ZnO	15 µg/mL	48 h	83,72342	3,5878	23,33559	4,34E-28	0,05	1	69,33391	98,11292
ZnO	10 µg/mL	48 h	ZnO	15 µg/mL	72 h	79,33928	3,5878	22,11363	6,60E-27	0,05	1	64,94978	93,72879
ZnO	10 µg/mL	48 h	ZnO	20 µg/mL	24 h	107,4836	3,35608	32,02653	2,97E-35	0,05	1	94,02342	120,9437
ZnO	10 µg/mL	48 h	ZnO	20 µg/mL	48 h	108,108	3,5878	30,1321	7,50E-34	0,05	1	93,71846	122,4975
ZnO	10 µg/mL	48 h	ZnO	20 µg/mL	72 h	108,0701	3,5878	30,12156	7,64E-34	0,05	1	93,68063	122,4596
ZnO	10 µg/mL	48 h	ZnO	25 µg/mL	24 h	108,3335	3,35608	32,27979	1,95E-35	0,05	1	94,87339	121,7937
ZnO	10 µg/mL	48 h	ZnO	25 µg/mL	48 h	109,1957	3,5878	30,43529	4,42E-34	0,05	1	94,80623	123,5852
ZnO	10 µg/mL	48 h	ZnO	25 µg/mL	72 h	109,8038	3,5878	30,60478	3,30E-34	0,05	1	95,41434	124,1934
ZnO	10 µg/mL	72 h	ZnO	15 µg/mL	24 h	78,90262	3,35608	23,51036	2,97E-28	0,05	1	65,44247	92,36277
ZnO	10 µg/mL	72 h	ZnO	15 µg/mL	48 h	71,15247	3,5878	19,83178	1,49E-24	0,05	1	56,76297	85,54198
ZnO	10 µg/mL	72 h	ZnO	15 µg/mL	72 h	66,76833	3,5878	18,60983	3,31E-23	0,05	1	52,37883	81,15784
ZnO	10 µg/mL	72 h	ZnO	20 µg/mL	24 h	94,91262	3,35608	28,2808	2,11E-32	0,05	1	81,45247	108,3728
ZnO	10 µg/mL	72 h	ZnO	20 µg/mL	48 h	95,53702	3,5878	26,6283	4,88E-31	0,05	1	81,14751	109,9265
ZnO	10 µg/mL	72 h	ZnO	20 µg/mL	72 h	95,49919	3,5878	26,61776	4,98E-31	0,05	1	81,10969	109,8887
ZnO	10 µg/mL	72 h	ZnO	25 µg/mL	24 h	95,76259	3,35608	28,53406	1,32E-32	0,05	1	82,30244	109,2227
ZnO	10 µg/mL	72 h	ZnO	25 µg/mL	48 h	96,62479	3,5878	26,93149	2,71E-31	0,05	1	82,23529	111,0143
ZnO	10 µg/mL	72 h	ZnO	25 µg/mL	72 h	97,2329	3,5878	27,10098	1,95E-31	0,05	1	82,84339	111,6224
ZnO	15 µg/mL	24 h	ZnO	15 µg/mL	48 h	-7,75015	3,35608	-2,30929	1	0,05	0	-21,2103	5,71
ZnO	15 µg/mL	24 h	ZnO	15 µg/mL	72 h	-12,1343	3,35608	-3,61561	0,17752	0,05	0	-25,5944	1,32586
ZnO	15 µg/mL	24 h	ZnO	20 µg/mL	24 h	16,01	3,10713	5,15267	9,54E-04	0,05	1	3,54832	28,47168
ZnO	15 µg/mL	24 h	ZnO	20 µg/mL	48 h	16,6344	3,35608	4,9565	0,00193	0,05	1	3,17425	30,09454
ZnO	15 µg/mL	24 h	ZnO	20 µg/mL	72 h	16,59657	3,35608	4,94522	0,00201	0,05	1	3,13642	30,05672
ZnO	15 µg/mL	24 h	ZnO	25 µg/mL	24 h	16,85997	3,10713	5,42623	3,52E-04	0,05	1	4,39829	29,32165
ZnO	15 µg/mL	24 h	ZnO	25 µg/mL	48 h	17,72217	3,35608	5,28062	6,00E-04	0,05	1	4,26202	31,18232
ZnO	15 µg/mL	24 h	ZnO	25 µg/mL	72 h	18,33027	3,35608	5,46181	3,09E-04	0,05	1	4,87012	31,79042
ZnO	15 µg/mL	48 h	ZnO	15 µg/mL	72 h	-4,38414	3,5878	-1,22196	1	0,05	0	-18,7736	10,00537
ZnO	15 µg/mL	48 h	ZnO	20 µg/mL	24 h	23,76015	3,35608	7,07973	7,05E-07	0,05	1	10,3	37,2203
ZnO	15 µg/mL	48 h	ZnO	20 µg/mL	48 h	24,38455	3,5878	6,79652	2,07E-06	0,05	1	9,99504	38,77405
ZnO	15 µg/mL	48 h	ZnO	20 µg/mL	72 h	24,34672	3,5878	6,78598	2,16E-06	0,05	1	9,95722	38,73623
ZnO	15 µg/mL	48 h	ZnO	25 µg/mL	24 h	24,61012	3,35608	7,333	2,69E-07	0,05	1	11,14997	38,07027
ZnO	15 µg/mL	48 h	ZnO	25 µg/mL	48 h	25,47232	3,5878	7,0997	6,54E-07	0,05	1	11,08282	39,86183

ZnO	15 µg/mL	48 h	ZnO	25 µg/mL	72 h	26,08043	3,5878	7,2692	3,43E-07	0,05	1	11,69092	40,46993
ZnO	15 µg/mL	72 h	ZnO	20 µg/mL	24 h	28,14429	3,35608	8,38606	4,96E-09	0,05	1	14,68414	41,60444
ZnO	15 µg/mL	72 h	ZnO	20 µg/mL	48 h	28,76869	3,5878	8,01848	1,99E-08	0,05	1	14,37918	43,15819
ZnO	15 µg/mL	72 h	ZnO	20 µg/mL	72 h	28,73086	3,5878	8,00793	2,07E-08	0,05	1	14,34135	43,12036
ZnO	15 µg/mL	72 h	ZnO	25 µg/mL	24 h	28,99426	3,35608	8,63932	1,92E-09	0,05	1	15,53411	42,45441
ZnO	15 µg/mL	72 h	ZnO	25 µg/mL	48 h	29,85646	3,5878	8,32166	6,33E-09	0,05	1	15,46695	44,24596
ZnO	15 µg/mL	72 h	ZnO	25 µg/mL	72 h	30,46456	3,5878	8,49115	3,34E-09	0,05	1	16,07506	44,85407
ZnO	20 µg/mL	24 h	ZnO	20 µg/mL	48 h	0,6244	3,35608	0,18605	1	0,05	0	-12,8358	14,08455
ZnO	20 µg/mL	24 h	ZnO	20 µg/mL	72 h	0,58657	3,35608	0,17478	1	0,05	0	-12,8736	14,04672
ZnO	20 µg/mL	24 h	ZnO	25 µg/mL	24 h	0,84997	3,10713	0,27356	1	0,05	0	-11,6117	13,31165
ZnO	20 µg/mL	24 h	ZnO	25 µg/mL	48 h	1,71217	3,35608	0,51017	1	0,05	0	-11,748	15,17232
ZnO	20 µg/mL	24 h	ZnO	25 µg/mL	72 h	2,32028	3,35608	0,69137	1	0,05	0	-11,1399	15,78043
ZnO	20 µg/mL	48 h	ZnO	20 µg/mL	72 h	-0,03783	3,5878	-0,01054	1	0,05	0	-14,4273	14,35168
ZnO	20 µg/mL	48 h	ZnO	25 µg/mL	24 h	0,22557	3,35608	0,06721	1	0,05	0	-13,2346	13,68572
ZnO	20 µg/mL	48 h	ZnO	25 µg/mL	48 h	1,08777	3,5878	0,30319	1	0,05	0	-13,3017	15,47728
ZnO	20 µg/mL	48 h	ZnO	25 µg/mL	72 h	1,69588	3,5878	0,47268	1	0,05	0	-12,6936	16,08538
ZnO	20 µg/mL	72 h	ZnO	25 µg/mL	24 h	0,2634	3,35608	0,07848	1	0,05	0	-13,1968	13,72355
ZnO	20 µg/mL	72 h	ZnO	25 µg/mL	48 h	1,1256	3,5878	0,31373	1	0,05	0	-13,2639	15,5151
ZnO	20 µg/mL	72 h	ZnO	25 µg/mL	72 h	1,73371	3,5878	0,48322	1	0,05	0	-12,6558	16,12321
ZnO	25 µg/mL	24 h	ZnO	25 µg/mL	48 h	0,8622	3,35608	0,25691	1	0,05	0	-12,598	14,32235
ZnO	25 µg/mL	24 h	ZnO	25 µg/mL	72 h	1,47031	3,35608	0,4381	1	0,05	0	-11,9898	14,93046
ZnO	25 µg/mL	48 h	ZnO	25 µg/mL	72 h	0,60811	3,5878	0,16949	1	0,05	0	-13,7814	14,99761

Statistical analysis for ZnO and Fe:ZnO cytotoxicity on HPDE cell line. Bonferroni t-test (Three Ways ANOVA)

**NPs**								
	MeanDiff	SEM	t Value	Prob	Alpha	Sig	LCL	UCL
Fe:ZnO ZnO	11,82373	1,96413	6,01983	2,34E-07	0,05	1	7,87458	15,77288
**Dose**								
	MeanDiff	SEM	t Value	Prob	Alpha	Sig	LCL	UCL
10 µg/mL 15 µg/mL	-19,56309	2,7777	-7,04291	3,79E-08	0,05	1	-27,20738	-11,91879
10 µg/mL 20 µg/mL	33,4463	2,7777	12,041	2,48E-15	0,05	1	25,802	41,0906
10 µg/mL 25 µg/mL	84,43646	2,7777	30,39797	3,11E-32	0,05	1	76,79216	92,08075
15 µg/mL 20 µg/mL	53,00939	2,7777	19,08391	2,82E-23	0,05	1	45,36509	60,65369
15 µg/mL 25 µg/mL	103,99954	2,7777	37,44087	2,10E-36	0,05	1	96,35525	111,64384
20 µg/mL 25 µg/mL	50,99015	2,7777	18,35697	1,46E-22	0,05	1	43,34586	58,63445
**Incubation time**								
	MeanDiff	SEM	t Value	Prob	Alpha	Sig	LCL	UCL
24 h 48 h	5,53297	2,40556	2,30008	0,0775	0,05	0	-0,4347	11,50065
24 h 72 h	12,02317	2,40556	4,99808	2,434E-05	0,05	1	6,0555	17,99085

48 h 72 h	6,4902	2,40556	2,698	0,02879	0,05	1	0,52253	12,45787

Interactions

NPs	Dose	Incubation time	NPs	Dose	Incubation time	MeanDiff	SEM	t Value	Prob	Alpha	Sig	LCL	UCL
Fe:ZnO	10 µg/mL	--	Fe:ZnO	15 µg/mL	--	-19,49321	3,92826	-4,9623	0,0002566	0,05	1	-32,48824	-6,49818
Fe:ZnO	10 µg/mL	--	Fe:ZnO	20 µg/mL	--	-3,24692	3,92826	-0,82655	1,00E+00	0,05	0	-16,24195	9,74811
Fe:ZnO	10 µg/mL	--	Fe:ZnO	25 µg/mL	--	64,14398	3,92826	16,32885	8,77E-20	0,05	1	51,14895	77,13902
Fe:ZnO	10 µg/mL	--	ZnO	10 µg/mL	--	-16,63418	3,92826	-4,23449	0,00288	0,05	1	-29,62921	-3,63915
Fe:ZnO	10 µg/mL	--	ZnO	15 µg/mL	--	-36,26714	3,92826	-9,23236	9,06E-11	0,05	1	-49,26217	-23,27211
Fe:ZnO	10 µg/mL	--	ZnO	20 µg/mL	--	53,50535	3,92826	13,62062	1,16E-16	0,05	1	40,51032	66,50038
Fe:ZnO	10 µg/mL	--	ZnO	25 µg/mL	--	88,09475	3,92826	22,42589	1,23E-25	0,05	1	75,09972	101,08978
Fe:ZnO	15 µg/mL	--	Fe:ZnO	20 µg/mL	--	16,24629	3,92826	4,13574	3,96E-03	0,05	1	3,25125	29,24132
Fe:ZnO	15 µg/mL	--	Fe:ZnO	25 µg/mL	--	83,63719	3,92826	21,29114	1,19E-24	0,05	1	70,64216	96,63222
Fe:ZnO	15 µg/mL	--	ZnO	10 µg/mL	--	2,85903	3,92826	0,72781	1,00E+00	0,05	0	-10,136	15,85406
Fe:ZnO	15 µg/mL	--	ZnO	15 µg/mL	--	-16,77393	3,92826	-4,27006	2,57E-03	0,05	1	-29,76896	-3,7789
Fe:ZnO	15 µg/mL	--	ZnO	20 µg/mL	--	72,99856	3,92826	18,58292	4,06E-22	0,05	1	60,00353	85,99359
Fe:ZnO	15 µg/mL	--	ZnO	25 µg/mL	--	107,58796	3,92826	27,38818	1,66E-29	0,05	1	94,59293	120,58299
Fe:ZnO	20 µg/mL	--	Fe:ZnO	25 µg/mL	--	67,39091	3,92826	17,1554	1,15E-20	0,05	1	54,39587	80,38594
Fe:ZnO	20 µg/mL	--	ZnO	10 µg/mL	--	-13,38725	3,92826	-3,40793	3,73E-02	0,05	1	-26,38229	-0,39222
Fe:ZnO	20 µg/mL	--	ZnO	15 µg/mL	--	-33,02022	3,92826	-8,40581	1,513E-09	0,05	1	-46,01525	-20,02519
Fe:ZnO	20 µg/mL	--	ZnO	20 µg/mL	--	56,75227	3,92826	14,44717	1,18E-17	0,05	1	43,75724	69,7473
Fe:ZnO	20 µg/mL	--	ZnO	25 µg/mL	--	91,34167	3,92826	23,25244	2,49E-26	0,05	1	78,34664	104,3367
Fe:ZnO	25 µg/mL	--	ZnO	10 µg/mL	--	-80,77816	3,92826	-20,56333	5,36E-24	0,05	1	-93,77319	-67,78313
Fe:ZnO	25 µg/mL	--	ZnO	15 µg/mL	--	-100,41112	3,92826	-25,56121	3,70E-28	0,05	1	-113,40616	-87,41609
Fe:ZnO	25 µg/mL	--	ZnO	20 µg/mL	--	-10,63863	3,92826	-2,70823	0,26168	0,05	0	-23,63367	2,3564
Fe:ZnO	25 µg/mL	--	ZnO	25 µg/mL	--	23,95077	3,92826	6,09704	4,99E-06	0,05	1	10,95574	36,9458
ZnO	10 µg/mL	--	ZnO	15 µg/mL	--	-19,63296	3,92826	-4,99788	2,27E-04	0,05	1	-32,628	-6,63793
ZnO	10 µg/mL	--	ZnO	20 µg/mL	--	70,13953	3,92826	17,8551	2,18E-21	0,05	1	57,14449	83,13456
ZnO	10 µg/mL	--	ZnO	25 µg/mL	--	104,72893	3,92826	26,66037	5,58E-29	0,05	1	91,7339	117,72396
ZnO	15 µg/mL	--	ZnO	20 µg/mL	--	89,77249	3,92826	22,85298	5,35E-26	0,05	1	76,77746	102,76752
ZnO	15 µg/mL	--	ZnO	25 µg/mL	--	124,36189	3,92826	31,65825	2,26E-32	0,05	1	111,36686	137,35692
ZnO	20 µg/mL	--	ZnO	25 µg/mL	--	34,5894	3,92826	8,80527	3,848E-10	0,05	1	21,59437	47,58443
Fe:ZnO	--	24 h	Fe:ZnO	--	48 h	5,50816	3,40197	1,61911	1	0,05	0	-5,00134	16,01765
Fe:ZnO	--	24 h	Fe:ZnO	--	72 h	10,55467	3,40197	3,10251	0,04817	0,05	1	0,04517	21,06416
Fe:ZnO	--	24 h	ZnO	--	24 h	10,82818	3,40197	3,18291	3,84E-02	0,05	1	0,31869	21,33768
Fe:ZnO	--	24 h	ZnO	--	48 h	16,38597	3,40197	4,81661	2,25E-04	0,05	1	5,87648	26,89547
Fe:ZnO	--	24 h	ZnO	--	72 h	24,31986	3,40197	7,14875	6,53E-08	0,05	1	13,81037	34,82936
Fe:ZnO	--	48 h	Fe:ZnO	--	72 h	5,04651	3,40197	1,48341	1	0,05	0	-5,46299	15,55601
Fe:ZnO	--	48 h	ZnO	--	24 h	5,32003	3,40197	1,56381	1,00E+00	0,05	0	-5,18947	15,82952
Fe:ZnO	--	48 h	ZnO	--	48 h	10,87782	3,40197	3,1975	3,68E-02	0,05	1	0,36832	21,38732
Fe:ZnO	--	48 h	ZnO	--	72 h	18,81171	3,40197	5,52964	1,95E-05	0,05	1	8,30221	29,3212
Fe:ZnO	--	72 h	ZnO	--	24 h	0,27352	3,40197	0,0804	1,00E+00	0,05	0	-10,23598	10,78301

Fe:ZnO	--	72 h	ZnO	--	48 h	5,83131	3,40197	1,7141	1,00E+00	0,05	0	-4,67819	16,3408
Fe:ZnO	--	72 h	ZnO	--	72 h	13,7652	3,40197	4,04624	2,82E-03	0,05	1	3,2557	24,27469
ZnO	--	24 h	ZnO	--	48 h	5,55779	3,40197	1,6337	1	0,05	0	-4,9517	16,06729
ZnO	--	24 h	ZnO	--	72 h	13,49168	3,40197	3,96584	0,00364	0,05	1	2,98218	24,00118
ZnO	--	48 h	ZnO	--	72 h	7,93389	3,40197	2,33214	0,35903	0,05	0	-2,57561	18,44338
--	10 µg/mL	24 h	--	10 µg/mL	48 h	-3,78414	4,81112	-0,78654	1	0,05	0	-21,09224	13,52395
--	10 µg/mL	24 h	--	10 µg/mL	72 h	-5,27383	4,81112	-1,09618	1	0,05	0	-22,58193	12,03427
--	10 µg/mL	24 h	--	15 µg/mL	24 h	-25,85232	4,81112	-5,37345	1,48E-04	0,05	1	-43,16041	-8,54422
--	10 µg/mL	24 h	--	15 µg/mL	48 h	-30,6458	4,81112	-6,36979	4,50E-06	0,05	1	-47,9539	-13,3377
--	10 µg/mL	24 h	--	15 µg/mL	72 h	-11,24912	4,81112	-2,33815	1,00E+00	0,05	0	-28,55722	6,05898
--	10 µg/mL	24 h	--	20 µg/mL	24 h	15,55901	4,81112	3,23397	1,46E-01	0,05	0	-1,74909	32,86711
--	10 µg/mL	24 h	--	20 µg/mL	48 h	32,3461	4,81112	6,7232	1,29E-06	0,05	1	15,038	49,6542
--	10 µg/mL	24 h	--	20 µg/mL	72 h	43,37582	4,81112	9,01575	4,44E-10	0,05	1	26,06772	60,68392
--	10 µg/mL	24 h	--	25 µg/mL	24 h	73,12748	4,81112	15,19968	3,73E-18	0,05	1	55,81938	90,43558
--	10 µg/mL	24 h	--	25 µg/mL	48 h	87,04991	4,81112	18,09349	2,95E-21	0,05	1	69,74182	104,35801
--	10 µg/mL	24 h	--	25 µg/mL	72 h	84,074	4,81112	17,47494	1,26E-20	0,05	1	66,7659	101,38209
--	10 µg/mL	48 h	--	10 µg/mL	72 h	-1,48969	4,81112	-0,30963	1	0,05	0	-18,79779	15,81841
--	10 µg/mL	48 h	--	15 µg/mL	24 h	-22,06817	4,81112	-4,58691	2,14E-03	0,05	1	-39,37627	-4,76008
--	10 µg/mL	48 h	--	15 µg/mL	48 h	-26,86166	4,81112	-5,58325	7,12E-05	0,05	1	-44,16975	-9,55356
--	10 µg/mL	48 h	--	15 µg/mL	72 h	-7,46498	4,81112	-1,55161	1,00E+00	0,05	0	-24,77307	9,84312
--	10 µg/mL	48 h	--	20 µg/mL	24 h	19,34315	4,81112	4,02051	1,35E-02	0,05	1	2,03506	36,65125
--	10 µg/mL	48 h	--	20 µg/mL	48 h	36,13024	4,81112	7,50974	8,06E-08	0,05	1	18,82214	53,43834
--	10 µg/mL	48 h	--	20 µg/mL	72 h	47,15996	4,81112	9,80229	3,20E-11	0,05	1	29,85187	64,46806
--	10 µg/mL	48 h	--	25 µg/mL	24 h	76,91162	4,81112	15,98622	4,90E-19	0,05	1	59,60353	94,21972
--	10 µg/mL	48 h	--	25 µg/mL	48 h	90,83406	4,81112	18,88003	4,90E-22	0,05	1	73,52596	108,14215
--	10 µg/mL	48 h	--	25 µg/mL	72 h	87,85814	4,81112	18,26148	2,00E-21	0,05	1	70,55004	105,16624
--	10 µg/mL	72 h	--	15 µg/mL	24 h	-20,57849	4,81112	-4,27728	5,92E-03	0,05	1	-37,88658	-3,27039
--	10 µg/mL	72 h	--	15 µg/mL	48 h	-25,37197	4,81112	-5,27361	2,08E-04	0,05	1	-42,68006	-8,06387
--	10 µg/mL	72 h	--	15 µg/mL	72 h	-5,97529	4,81112	-1,24197	1,00E+00	0,05	0	-23,28338	11,33281
--	10 µg/mL	72 h	--	20 µg/mL	24 h	20,83284	4,81112	4,33015	4,98E-03	0,05	1	3,52474	38,14094
--	10 µg/mL	72 h	--	20 µg/mL	48 h	37,61993	4,81112	7,81937	2,73E-08	0,05	1	20,31183	54,92803
--	10 µg/mL	72 h	--	20 µg/mL	72 h	48,64965	4,81112	10,11192	1,16E-11	0,05	1	31,34156	65,95775
--	10 µg/mL	72 h	--	25 µg/mL	24 h	78,40131	4,81112	16,29586	2,25E-19	0,05	1	61,09322	95,70941
--	10 µg/mL	72 h	--	25 µg/mL	48 h	92,32374	4,81112	19,18966	2,45E-22	0,05	1	75,01565	109,63184
--	10 µg/mL	72 h	--	25 µg/mL	72 h	89,34783	4,81112	18,57111	9,84E-22	0,05	1	72,03973	106,65593
--	15 µg/mL	24 h	--	15 µg/mL	48 h	-4,79348	4,81112	-0,99633	1	0,05	0	-22,10158	12,51462
--	15 µg/mL	24 h	--	15 µg/mL	72 h	14,6032	4,81112	3,0353	0,2557	0,05	0	-2,7049	31,9113
--	15 µg/mL	24 h	--	20 µg/mL	24 h	41,41133	4,81112	8,60742	1,78E-09	0,05	1	24,10323	58,71943
--	15 µg/mL	24 h	--	20 µg/mL	48 h	58,19842	4,81112	12,09665	2,30E-14	0,05	1	40,89032	75,50651
--	15 µg/mL	24 h	--	20 µg/mL	72 h	69,22814	4,81112	14,3892	3,26E-17	0,05	1	51,92004	86,53624
--	15 µg/mL	24 h	--	25 µg/mL	24 h	98,9798	4,81112	20,57314	1,24E-23	0,05	1	81,6717	116,2879

--	15 µg/mL	24 h	--	25 µg/mL	48 h	112,90223	4,81112	23,46694	3,92E-26	0,05	1	95,59413	130,21033
--	15 µg/mL	24 h	--	25 µg/mL	72 h	109,92631	4,81112	22,84839	1,27E-25	0,05	1	92,61822	127,23441
--	15 µg/mL	48 h	--	15 µg/mL	72 h	19,39668	4,81112	4,03164	0,01302	0,05	1	2,08858	36,70478
--	15 µg/mL	48 h	--	20 µg/mL	24 h	46,20481	4,81112	9,60376	6,17E-11	0,05	1	28,89671	63,51291
--	15 µg/mL	48 h	--	20 µg/mL	48 h	62,9919	4,81112	13,09298	1,23E-15	0,05	1	45,6838	80,3
--	15 µg/mL	48 h	--	20 µg/mL	72 h	74,02162	4,81112	15,38553	2,30E-18	0,05	1	56,71352	91,32972
--	15 µg/mL	48 h	--	25 µg/mL	24 h	103,77328	4,81112	21,56947	1,59E-24	0,05	1	86,46518	121,08138
--	15 µg/mL	48 h	--	25 µg/mL	48 h	117,69571	4,81112	24,46327	6,19E-27	0,05	1	100,38761	135,00381
--	15 µg/mL	48 h	--	25 µg/mL	72 h	114,7198	4,81112	23,84472	1,93E-26	0,05	1	97,4117	132,02789
--	15 µg/mL	72 h	--	20 µg/mL	24 h	26,80813	4,81112	5,57212	7,40E-05	0,05	1	9,50003	44,11623
--	15 µg/mL	72 h	--	20 µg/mL	48 h	43,59522	4,81112	9,06135	3,80E-10	0,05	1	26,28712	60,90331
--	15 µg/mL	72 h	--	20 µg/mL	72 h	54,62494	4,81112	11,3539	2,23E-13	0,05	1	37,31684	71,93304
--	15 µg/mL	72 h	--	25 µg/mL	24 h	84,3766	4,81112	17,53783	1,09E-20	0,05	1	67,0685	101,6847
--	15 µg/mL	72 h	--	25 µg/mL	48 h	98,29903	4,81112	20,43164	1,67E-23	0,05	1	80,99093	115,60713
--	15 µg/mL	72 h	--	25 µg/mL	72 h	95,32312	4,81112	19,81309	6,26E-23	0,05	1	78,01502	112,63121
--	20 µg/mL	24 h	--	20 µg/mL	48 h	16,78709	4,81112	3,48923	0,06919	0,05	0	-0,52101	34,09519
--	20 µg/mL	24 h	--	20 µg/mL	72 h	27,81681	4,81112	5,78178	3,559E-05	0,05	1	10,50871	45,12491
--	20 µg/mL	24 h	--	25 µg/mL	24 h	57,56847	4,81112	11,96571	3,418E-14	0,05	1	40,26037	74,87657
--	20 µg/mL	24 h	--	25 µg/mL	48 h	71,4909	4,81112	14,85952	9,178E-18	0,05	1	54,18281	88,799
--	20 µg/mL	24 h	--	25 µg/mL	72 h	68,51499	4,81112	14,24097	4,88E-17	0,05	1	51,20689	85,82308
--	20 µg/mL	48 h	--	20 µg/mL	72 h	11,02972	4,81112	2,29255	1	0,05	0	-6,27838	28,33782
--	20 µg/mL	48 h	--	25 µg/mL	24 h	40,78138	4,81112	8,47649	2,796E-09	0,05	1	23,47328	58,08948
--	20 µg/mL	48 h	--	25 µg/mL	48 h	54,70381	4,81112	11,37029	2,117E-13	0,05	1	37,39572	72,01191
--	20 µg/mL	48 h	--	25 µg/mL	72 h	51,7279	4,81112	10,75174	1,477E-12	0,05	1	34,4198	69,036
--	20 µg/mL	72 h	--	25 µg/mL	24 h	29,75166	4,81112	6,18394	8,66E-06	0,05	1	12,44356	47,05976
--	20 µg/mL	72 h	--	25 µg/mL	48 h	43,67409	4,81112	9,07774	3,598E-10	0,05	1	26,36599	60,98219
--	20 µg/mL	72 h	--	25 µg/mL	72 h	40,69818	4,81112	8,45919	2,967E-09	0,05	1	23,39008	58,00627
--	25 µg/mL	24 h	--	25 µg/mL	48 h	13,92243	4,81112	2,8938	0,3768	0,05	0	-3,38567	31,23053
--	25 µg/mL	24 h	--	25 µg/mL	72 h	10,94652	4,81112	2,27525	1	0,05	0	-6,36158	28,25461
--	25 µg/mL	48 h	--	25 µg/mL	72 h	-2,97592	4,81112	-0,61855	1	0,05	0	-20,28401	14,33218
Fe:ZnO	10 µg/mL	24 h	Fe:ZnO	10 µg/mL	48 h	-1,08295	6,80395	-0,15917	1	0,05	0	-28,69583	26,52992
Fe:ZnO	10 µg/mL	24 h	Fe:ZnO	10 µg/mL	72 h	-11,06448	6,80395	-1,62619	1	0,05	0	-38,67736	16,54839
Fe:ZnO	10 µg/mL	24 h	Fe:ZnO	15 µg/mL	24 h	-21,35949	6,80395	-3,13928	0,79919	0,05	0	-48,97236	6,25339
Fe:ZnO	10 µg/mL	24 h	Fe:ZnO	15 µg/mL	48 h	-30,3031	6,80395	-4,45375	0,01389	0,05	1	-57,91597	-2,69022
Fe:ZnO	10 µg/mL	24 h	Fe:ZnO	15 µg/mL	72 h	-18,96448	6,80395	-2,78728	1	0,05	0	-46,57736	8,64839
Fe:ZnO	10 µg/mL	24 h	Fe:ZnO	20 µg/mL	24 h	-23,44223	6,80395	-3,44539	3,30E-01	0,05	0	-51,05511	4,17064
Fe:ZnO	10 µg/mL	24 h	Fe:ZnO	20 µg/mL	48 h	-11,59082	6,80395	-1,70354	1,00E+00	0,05	0	-39,20369	16,02206
Fe:ZnO	10 µg/mL	24 h	Fe:ZnO	20 µg/mL	72 h	13,14484	6,80395	1,93194	1,00E+00	0,05	0	-14,46803	40,75772
Fe:ZnO	10 µg/mL	24 h	Fe:ZnO	25 µg/mL	24 h	48,59189	6,80395	7,14172	1,23E-06	0,05	1	20,97901	76,20477
Fe:ZnO	10 µg/mL	24 h	Fe:ZnO	25 µg/mL	48 h	68,79966	6,80395	10,11172	4,85E-11	0,05	1	41,18679	96,41254
Fe:ZnO	10 µg/mL	24 h	Fe:ZnO	25 µg/mL	72 h	62,89296	6,80395	9,2436	8,60E-10	0,05	1	35,28009	90,50584

Fe:ZnO	10 µg/mL	24 h	ZnO	10 µg/mL	24 h	-18,69382	6,80395	-2,7475	1,00E+00	0,05	0	-46,3067	8,91906
Fe:ZnO	10 µg/mL	24 h	ZnO	10 µg/mL	48 h	-25,17915	6,80395	-3,70067	0,1528	0,05	0	-52,79203	2,43372
Fe:ZnO	10 µg/mL	24 h	ZnO	10 µg/mL	72 h	-18,177	6,80395	-2,67154	1	0,05	0	-45,78987	9,43588
Fe:ZnO	10 µg/mL	24 h	ZnO	15 µg/mL	24 h	-49,03897	6,80395	-7,20743	9,77E-07	0,05	1	-76,65184	-21,42609
Fe:ZnO	10 µg/mL	24 h	ZnO	15 µg/mL	48 h	-49,68232	6,80395	-7,30198	7,00E-07	0,05	1	-77,2952	-22,06944
Fe:ZnO	10 µg/mL	24 h	ZnO	15 µg/mL	72 h	-22,22757	6,80395	-3,26686	5,55E-01	0,05	0	-49,84045	5,3853
Fe:ZnO	10 µg/mL	24 h	ZnO	20 µg/mL	24 h	35,86643	6,80395	5,27141	8,78E-04	0,05	1	8,25356	63,47931
Fe:ZnO	10 µg/mL	24 h	ZnO	20 µg/mL	48 h	57,5892	6,80395	8,46408	1,22E-08	0,05	1	29,97632	85,20207
Fe:ZnO	10 µg/mL	24 h	ZnO	20 µg/mL	72 h	54,91298	6,80395	8,07075	4,75E-08	0,05	1	27,3001	82,52585
Fe:ZnO	10 µg/mL	24 h	ZnO	25 µg/mL	24 h	78,96926	6,80395	11,60639	4,27E-13	0,05	1	51,35638	106,58213
Fe:ZnO	10 µg/mL	24 h	ZnO	25 µg/mL	48 h	86,60635	6,80395	12,72883	1,48E-14	0,05	1	58,99347	114,21922
Fe:ZnO	10 µg/mL	24 h	ZnO	25 µg/mL	72 h	86,56121	6,80395	12,7222	1,51E-14	0,05	1	58,94834	114,17409
Fe:ZnO	10 µg/mL	48 h	Fe:ZnO	10 µg/mL	72 h	-9,98153	6,80395	-1,46702	1	0,05	0	-37,59441	17,63134
Fe:ZnO	10 µg/mL	48 h	Fe:ZnO	15 µg/mL	24 h	-20,27653	6,80395	-2,98011	1	0,05	0	-47,88941	7,33634
Fe:ZnO	10 µg/mL	48 h	Fe:ZnO	15 µg/mL	48 h	-29,22014	6,80395	-4,29459	0,02338	0,05	1	-56,83302	-1,60727
Fe:ZnO	10 µg/mL	48 h	Fe:ZnO	15 µg/mL	72 h	-17,88153	6,80395	-2,62811	1	0,05	0	-45,49441	9,73135
Fe:ZnO	10 µg/mL	48 h	Fe:ZnO	20 µg/mL	24 h	-22,35928	6,80395	-3,28622	5,25E-01	0,05	0	-49,97216	5,2536
Fe:ZnO	10 µg/mL	48 h	Fe:ZnO	20 µg/mL	48 h	-10,50786	6,80395	-1,54438	1,00E+00	0,05	0	-38,12074	17,10501
Fe:ZnO	10 µg/mL	48 h	Fe:ZnO	20 µg/mL	72 h	14,2278	6,80395	2,09111	1,00E+00	0,05	0	-13,38508	41,84067
Fe:ZnO	10 µg/mL	48 h	Fe:ZnO	25 µg/mL	24 h	49,67484	6,80395	7,30088	7,03E-07	0,05	1	22,06197	77,28772
Fe:ZnO	10 µg/mL	48 h	Fe:ZnO	25 µg/mL	48 h	69,88262	6,80395	10,27089	2,89E-11	0,05	1	42,26974	97,49549
Fe:ZnO	10 µg/mL	48 h	Fe:ZnO	25 µg/mL	72 h	63,97591	6,80395	9,40276	5,05E-10	0,05	1	36,36304	91,58879
Fe:ZnO	10 µg/mL	48 h	ZnO	10 µg/mL	24 h	-17,61087	6,80395	-2,58833	1	0,05	0	-45,22374	10,00201
Fe:ZnO	10 µg/mL	48 h	ZnO	10 µg/mL	48 h	-24,0962	6,80395	-3,5415	0,2475	0,05	0	-51,70907	3,51668
Fe:ZnO	10 µg/mL	48 h	ZnO	10 µg/mL	72 h	-17,09404	6,80395	-2,51237	1	0,05	0	-44,70692	10,51883
Fe:ZnO	10 µg/mL	48 h	ZnO	15 µg/mL	24 h	-47,95602	6,80395	-7,04826	1,71E-06	0,05	1	-75,56889	-20,34314
Fe:ZnO	10 µg/mL	48 h	ZnO	15 µg/mL	48 h	-48,59937	6,80395	-7,14282	1,23E-06	0,05	1	-76,21224	-20,98649
Fe:ZnO	10 µg/mL	48 h	ZnO	15 µg/mL	72 h	-21,14462	6,80395	-3,1077	8,74E-01	0,05	0	-48,7575	6,46826
Fe:ZnO	10 µg/mL	48 h	ZnO	20 µg/mL	24 h	36,94939	6,80395	5,43058	5,06E-04	0,05	1	9,33651	64,56226
Fe:ZnO	10 µg/mL	48 h	ZnO	20 µg/mL	48 h	58,67215	6,80395	8,62325	7,06E-09	0,05	1	31,05927	86,28503
Fe:ZnO	10 µg/mL	48 h	ZnO	20 µg/mL	72 h	55,99593	6,80395	8,22992	2,74E-08	0,05	1	28,38306	83,60881
Fe:ZnO	10 µg/mL	48 h	ZnO	25 µg/mL	24 h	80,05221	6,80395	11,76555	2,63E-13	0,05	1	52,43933	107,66508
Fe:ZnO	10 µg/mL	48 h	ZnO	25 µg/mL	48 h	87,6893	6,80395	12,888	9,28E-15	0,05	1	60,07642	115,30217
Fe:ZnO	10 µg/mL	48 h	ZnO	25 µg/mL	72 h	87,64417	6,80395	12,88137	9,46E-15	0,05	1	60,03129	115,25704
Fe:ZnO	10 µg/mL	72 h	Fe:ZnO	15 µg/mL	24 h	-10,295	6,80395	-1,51309	1	0,05	0	-37,90788	17,31788
Fe:ZnO	10 µg/mL	72 h	Fe:ZnO	15 µg/mL	48 h	-19,23861	6,80395	-2,82757	1	0,05	0	-46,85149	8,37427
Fe:ZnO	10 µg/mL	72 h	Fe:ZnO	15 µg/mL	72 h	-7,9	6,80395	-1,16109	1	0,05	0	-35,51287	19,71288
Fe:ZnO	10 µg/mL	72 h	Fe:ZnO	20 µg/mL	24 h	-12,37775	6,80395	-1,8192	1,00E+00	0,05	0	-39,99062	15,23513
Fe:ZnO	10 µg/mL	72 h	Fe:ZnO	20 µg/mL	48 h	-0,52633	6,80395	-0,07736	1,00E+00	0,05	0	-28,13921	27,08654
Fe:ZnO	10 µg/mL	72 h	Fe:ZnO	20 µg/mL	72 h	24,20933	6,80395	3,55813	2,35E-01	0,05	0	-3,40355	51,82221
Fe:ZnO	10 µg/mL	72 h	Fe:ZnO	25 µg/mL	24 h	59,65637	6,80395	8,7679	4,31E-09	0,05	1	32,0435	87,26925

Fe:ZnO	10 µg/mL	72 h	Fe:ZnO	25 µg/mL	48 h	79,86415	6,80395	11,73791	2,86E-13	0,05	1	52,25127	107,47702
Fe:ZnO	10 µg/mL	72 h	Fe:ZnO	25 µg/mL	72 h	73,95745	6,80395	10,86978	4,25E-12	0,05	1	46,34457	101,57032
Fe:ZnO	10 µg/mL	72 h	ZnO	10 µg/mL	24 h	-7,62933	6,80395	-1,12131	1,00E+00	0,05	0	-35,24221	19,98354
Fe:ZnO	10 µg/mL	72 h	ZnO	10 µg/mL	48 h	-14,11467	6,80395	-2,07448	1	0,05	0	-41,72754	13,49821
Fe:ZnO	10 µg/mL	72 h	ZnO	10 µg/mL	72 h	-7,11251	6,80395	-1,04535	1	0,05	0	-34,72539	20,50036
Fe:ZnO	10 µg/mL	72 h	ZnO	15 µg/mL	24 h	-37,97448	6,80395	-5,58124	3,00E-04	0,05	1	-65,58736	-10,36161
Fe:ZnO	10 µg/mL	72 h	ZnO	15 µg/mL	48 h	-38,61784	6,80395	-5,6758	2,16E-04	0,05	1	-66,23071	-11,00496
Fe:ZnO	10 µg/mL	72 h	ZnO	15 µg/mL	72 h	-11,16309	6,80395	-1,64068	1,00E+00	0,05	0	-38,77596	16,44979
Fe:ZnO	10 µg/mL	72 h	ZnO	20 µg/mL	24 h	46,93092	6,80395	6,8976	2,91E-06	0,05	1	19,31804	74,5438
Fe:ZnO	10 µg/mL	72 h	ZnO	20 µg/mL	48 h	68,65368	6,80395	10,09027	5,20E-11	0,05	1	41,04081	96,26656
Fe:ZnO	10 µg/mL	72 h	ZnO	20 µg/mL	72 h	65,97746	6,80395	9,69694	1,90E-10	0,05	1	38,36459	93,59034
Fe:ZnO	10 µg/mL	72 h	ZnO	25 µg/mL	24 h	90,03374	6,80395	13,23257	3,44E-15	0,05	1	62,42086	117,64662
Fe:ZnO	10 µg/mL	72 h	ZnO	25 µg/mL	48 h	97,67083	6,80395	14,35502	1,49E-16	0,05	1	70,05795	125,28371
Fe:ZnO	10 µg/mL	72 h	ZnO	25 µg/mL	72 h	97,6257	6,80395	14,34839	1,52E-16	0,05	1	70,01282	125,23857
Fe:ZnO	15 µg/mL	24 h	Fe:ZnO	15 µg/mL	48 h	-8,94361	6,80395	-1,31447	1	0,05	0	-36,55649	18,66927
Fe:ZnO	15 µg/mL	24 h	Fe:ZnO	15 µg/mL	72 h	2,395	6,80395	0,352	1,00E+00	0,05	0	-25,21787	30,00788
Fe:ZnO	15 µg/mL	24 h	Fe:ZnO	20 µg/mL	24 h	-2,08275	6,80395	-0,30611	1,00E+00	0,05	0	-29,69562	25,53013
Fe:ZnO	15 µg/mL	24 h	Fe:ZnO	20 µg/mL	48 h	9,76867	6,80395	1,43573	1,00E+00	0,05	0	-17,84421	37,38154
Fe:ZnO	15 µg/mL	24 h	Fe:ZnO	20 µg/mL	72 h	34,50433	6,80395	5,07122	1,75E-03	0,05	1	6,89145	62,11721
Fe:ZnO	15 µg/mL	24 h	Fe:ZnO	25 µg/mL	24 h	69,95137	6,80395	10,281	2,80E-11	0,05	1	42,3385	97,56425
Fe:ZnO	15 µg/mL	24 h	Fe:ZnO	25 µg/mL	48 h	90,15915	6,80395	13,251	3,26E-15	0,05	1	62,54627	117,77202
Fe:ZnO	15 µg/mL	24 h	Fe:ZnO	25 µg/mL	72 h	84,25245	6,80395	12,38287	4,09E-14	0,05	1	56,63957	111,86532
Fe:ZnO	15 µg/mL	24 h	ZnO	10 µg/mL	24 h	2,66567	6,80395	0,39178	1	0,05	0	-24,94721	30,27854
Fe:ZnO	15 µg/mL	24 h	ZnO	10 µg/mL	48 h	-3,81967	6,80395	-0,56139	1	0,05	0	-31,43254	23,79321
Fe:ZnO	15 µg/mL	24 h	ZnO	10 µg/mL	72 h	3,18249	6,80395	0,46774	1	0,05	0	-24,43039	30,79536
Fe:ZnO	15 µg/mL	24 h	ZnO	15 µg/mL	24 h	-27,67948	6,80395	-4,06815	4,85E-02	0,05	1	-55,29236	-0,06661
Fe:ZnO	15 µg/mL	24 h	ZnO	15 µg/mL	48 h	-28,32283	6,80395	-4,16271	3,58E-02	0,05	1	-55,93571	-0,70996
Fe:ZnO	15 µg/mL	24 h	ZnO	15 µg/mL	72 h	-0,86809	6,80395	-0,12759	1,00E+00	0,05	0	-28,48096	26,74479
Fe:ZnO	15 µg/mL	24 h	ZnO	20 µg/mL	24 h	57,22592	6,80395	8,41069	1,47E-08	0,05	1	29,61304	84,8388
Fe:ZnO	15 µg/mL	24 h	ZnO	20 µg/mL	48 h	78,94868	6,80395	11,60336	4,31E-13	0,05	1	51,33581	106,56156
Fe:ZnO	15 µg/mL	24 h	ZnO	20 µg/mL	72 h	76,27246	6,80395	11,21003	1,46E-12	0,05	1	48,65959	103,88534
Fe:ZnO	15 µg/mL	24 h	ZnO	25 µg/mL	24 h	100,32874	6,80395	14,74566	5,20E-17	0,05	1	72,71586	127,94162
Fe:ZnO	15 µg/mL	24 h	ZnO	25 µg/mL	48 h	107,96583	6,80395	15,86811	2,77E-18	0,05	1	80,35295	135,57871
Fe:ZnO	15 µg/mL	24 h	ZnO	25 µg/mL	72 h	107,9207	6,80395	15,86148	2,82E-18	0,05	1	80,30782	135,53358
Fe:ZnO	15 µg/mL	48 h	Fe:ZnO	15 µg/mL	72 h	11,33861	6,80395	1,66648	1	0,05	0	-16,27426	38,95149
Fe:ZnO	15 µg/mL	48 h	Fe:ZnO	20 µg/mL	24 h	6,86086	6,80395	1,00836	1,00E+00	0,05	0	-20,75201	34,47374
Fe:ZnO	15 µg/mL	48 h	Fe:ZnO	20 µg/mL	48 h	18,71228	6,80395	2,75021	1,00E+00	0,05	0	-8,9006	46,32515
Fe:ZnO	15 µg/mL	48 h	Fe:ZnO	20 µg/mL	72 h	43,44794	6,80395	6,38569	1,78E-05	0,05	1	15,83506	71,06082
Fe:ZnO	15 µg/mL	48 h	Fe:ZnO	25 µg/mL	24 h	78,89498	6,80395	11,59547	4,42E-13	0,05	1	51,28211	106,50786
Fe:ZnO	15 µg/mL	48 h	Fe:ZnO	25 µg/mL	48 h	99,10276	6,80395	14,56548	8,45E-17	0,05	1	71,48988	126,71563
Fe:ZnO	15 µg/mL	48 h	Fe:ZnO	25 µg/mL	72 h	93,19606	6,80395	13,69735	9,21E-16	0,05	1	65,58318	120,80893

Fe:ZnO	15 µg/mL	48 h	ZnO	10 µg/mL	24 h	11,60928	6,80395	1,70626	1,00E+00	0,05	0	-16,0036	39,22215
Fe:ZnO	15 µg/mL	48 h	ZnO	10 µg/mL	48 h	5,12394	6,80395	0,75308	1	0,05	0	-22,48893	32,73682
Fe:ZnO	15 µg/mL	48 h	ZnO	10 µg/mL	72 h	12,1261	6,80395	1,78221	1	0,05	0	-15,48678	39,73898
Fe:ZnO	15 µg/mL	48 h	ZnO	15 µg/mL	24 h	-18,73587	6,80395	-2,75368	1,00E+00	0,05	0	-46,34875	8,877
Fe:ZnO	15 µg/mL	48 h	ZnO	15 µg/mL	48 h	-19,37922	6,80395	-2,84823	1,00E+00	0,05	0	-46,9921	8,23365
Fe:ZnO	15 µg/mL	48 h	ZnO	15 µg/mL	72 h	8,07552	6,80395	1,18689	1,00E+00	0,05	0	-19,53735	35,6884
Fe:ZnO	15 µg/mL	48 h	ZnO	20 µg/mL	24 h	66,16953	6,80395	9,72517	1,73E-10	0,05	1	38,55665	93,78241
Fe:ZnO	15 µg/mL	48 h	ZnO	20 µg/mL	48 h	87,89229	6,80395	12,91783	8,51E-15	0,05	1	60,27942	115,50517
Fe:ZnO	15 µg/mL	48 h	ZnO	20 µg/mL	72 h	85,21607	6,80395	12,5245	2,69E-14	0,05	1	57,6032	112,82895
Fe:ZnO	15 µg/mL	48 h	ZnO	25 µg/mL	24 h	109,27235	6,80395	16,06014	1,70E-18	0,05	1	81,65948	136,88523
Fe:ZnO	15 µg/mL	48 h	ZnO	25 µg/mL	48 h	116,90944	6,80395	17,18259	1,06E-19	0,05	1	89,29656	144,52232
Fe:ZnO	15 µg/mL	48 h	ZnO	25 µg/mL	72 h	116,86431	6,80395	17,17595	1,08E-19	0,05	1	89,25143	144,47719
Fe:ZnO	15 µg/mL	72 h	Fe:ZnO	20 µg/mL	24 h	-4,47775	6,80395	-0,65811	1,00E+00	0,05	0	-32,09063	23,13513
Fe:ZnO	15 µg/mL	72 h	Fe:ZnO	20 µg/mL	48 h	7,37367	6,80395	1,08373	1,00E+00	0,05	0	-20,23921	34,98654
Fe:ZnO	15 µg/mL	72 h	Fe:ZnO	20 µg/mL	72 h	32,10933	6,80395	4,71922	5,75E-03	0,05	1	4,49645	59,7222
Fe:ZnO	15 µg/mL	72 h	Fe:ZnO	25 µg/mL	24 h	67,55637	6,80395	9,92899	8,82E-11	0,05	1	39,9435	95,16925
Fe:ZnO	15 µg/mL	72 h	Fe:ZnO	25 µg/mL	48 h	87,76415	6,80395	12,899	8,99E-15	0,05	1	60,15127	115,37702
Fe:ZnO	15 µg/mL	72 h	Fe:ZnO	25 µg/mL	72 h	81,85744	6,80395	12,03087	1,17E-13	0,05	1	54,24457	109,47032
Fe:ZnO	15 µg/mL	72 h	ZnO	10 µg/mL	24 h	0,27066	6,80395	0,03978	1,00E+00	0,05	0	-27,34221	27,88354
Fe:ZnO	15 µg/mL	72 h	ZnO	10 µg/mL	48 h	-6,21467	6,80395	-0,91339	1,00E+00	0,05	0	-33,82755	21,39821
Fe:ZnO	15 µg/mL	72 h	ZnO	10 µg/mL	72 h	0,78749	6,80395	0,11574	1	0,05	0	-26,82539	28,40036
Fe:ZnO	15 µg/mL	72 h	ZnO	15 µg/mL	24 h	-30,07449	6,80395	-4,42015	1,55E-02	0,05	1	-57,68736	-2,46161
Fe:ZnO	15 µg/mL	72 h	ZnO	15 µg/mL	48 h	-30,71784	6,80395	-4,51471	1,14E-02	0,05	1	-58,33071	-3,10496
Fe:ZnO	15 µg/mL	72 h	ZnO	15 µg/mL	72 h	-3,26309	6,80395	-0,47959	1,00E+00	0,05	0	-30,87597	24,34979
Fe:ZnO	15 µg/mL	72 h	ZnO	20 µg/mL	24 h	54,83092	6,80395	8,05869	4,96E-08	0,05	1	27,21804	82,44379
Fe:ZnO	15 µg/mL	72 h	ZnO	20 µg/mL	48 h	76,55368	6,80395	11,25136	1,28E-12	0,05	1	48,9408	104,16656
Fe:ZnO	15 µg/mL	72 h	ZnO	20 µg/mL	72 h	73,87746	6,80395	10,85803	4,41E-12	0,05	1	46,26459	101,49034
Fe:ZnO	15 µg/mL	72 h	ZnO	25 µg/mL	24 h	97,93374	6,80395	14,39366	1,35E-16	0,05	1	70,32086	125,54661
Fe:ZnO	15 µg/mL	72 h	ZnO	25 µg/mL	48 h	105,57083	6,80395	15,51611	6,84E-18	0,05	1	77,95795	133,1837
Fe:ZnO	15 µg/mL	72 h	ZnO	25 µg/mL	72 h	105,5257	6,80395	15,50948	6,96E-18	0,05	1	77,91282	133,13857
Fe:ZnO	20 µg/mL	24 h	Fe:ZnO	20 µg/mL	48 h	11,85141	6,80395	1,74184	1	0,05	0	-15,76146	39,46429
Fe:ZnO	20 µg/mL	24 h	Fe:ZnO	20 µg/mL	72 h	36,58708	6,80395	5,37733	0,0006087	0,05	1	8,9742	64,19995
Fe:ZnO	20 µg/mL	24 h	Fe:ZnO	25 µg/mL	24 h	72,03412	6,80395	10,5871	1,045E-11	0,05	1	44,42125	99,647
Fe:ZnO	20 µg/mL	24 h	Fe:ZnO	25 µg/mL	48 h	92,2419	6,80395	13,55711	1,366E-15	0,05	1	64,62902	119,85477
Fe:ZnO	20 µg/mL	24 h	Fe:ZnO	25 µg/mL	72 h	86,33519	6,80395	12,68898	1,659E-14	0,05	1	58,72232	113,94807
Fe:ZnO	20 µg/mL	24 h	ZnO	10 µg/mL	24 h	4,74841	6,80395	0,69789	1,00E+00	0,05	0	-22,86446	32,36129
Fe:ZnO	20 µg/mL	24 h	ZnO	10 µg/mL	48 h	-1,73692	6,80395	-0,25528	1,00E+00	0,05	0	-29,3498	25,87596
Fe:ZnO	20 µg/mL	24 h	ZnO	10 µg/mL	72 h	5,26524	6,80395	0,77385	1,00E+00	0,05	0	-22,34764	32,87811
Fe:ZnO	20 µg/mL	24 h	ZnO	15 µg/mL	24 h	-25,59674	6,80395	-3,76204	0,12654	0,05	0	-53,20961	2,01614
Fe:ZnO	20 µg/mL	24 h	ZnO	15 µg/mL	48 h	-26,24009	6,80395	-3,8566	0,0944	0,05	0	-53,85296	1,37279
Fe:ZnO	20 µg/mL	24 h	ZnO	15 µg/mL	72 h	1,21466	6,80395	0,17852	1,00E+00	0,05	0	-26,39822	28,82754

Fe:ZnO	20 µg/mL	24 h	ZnO	20 µg/mL	24 h	59,30867	6,80395	8,7168	5,13E-09	0,05	1	31,69579	86,92154
Fe:ZnO	20 µg/mL	24 h	ZnO	20 µg/mL	48 h	81,03143	6,80395	11,90947	1,695E-13	0,05	1	53,41855	108,64431
Fe:ZnO	20 µg/mL	24 h	ZnO	20 µg/mL	72 h	78,35521	6,80395	11,51614	5,64E-13	0,05	1	50,74233	105,96809
Fe:ZnO	20 µg/mL	24 h	ZnO	25 µg/mL	24 h	102,41149	6,80395	15,05177	2,304E-17	0,05	1	74,79861	130,02436
Fe:ZnO	20 µg/mL	24 h	ZnO	25 µg/mL	48 h	110,04858	6,80395	16,17422	1,275E-18	0,05	1	82,4357	137,66145
Fe:ZnO	20 µg/mL	24 h	ZnO	25 µg/mL	72 h	110,00345	6,80395	16,16759	1,296E-18	0,05	1	82,39057	137,61632
Fe:ZnO	20 µg/mL	48 h	Fe:ZnO	20 µg/mL	72 h	24,73566	6,80395	3,63549	0,18638	0,05	0	-2,87721	52,34854
Fe:ZnO	20 µg/mL	48 h	Fe:ZnO	25 µg/mL	24 h	60,18271	6,80395	8,84526	3,31E-09	0,05	1	32,56983	87,79558
Fe:ZnO	20 µg/mL	48 h	Fe:ZnO	25 µg/mL	48 h	80,39048	6,80395	11,81527	2,256E-13	0,05	1	52,7776	108,00336
Fe:ZnO	20 µg/mL	48 h	Fe:ZnO	25 µg/mL	72 h	74,48378	6,80395	10,94714	3,327E-12	0,05	1	46,8709	102,09666
Fe:ZnO	20 µg/mL	48 h	ZnO	10 µg/mL	24 h	-7,103	6,80395	-1,04395	1,00E+00	0,05	0	-34,71588	20,50987
Fe:ZnO	20 µg/mL	48 h	ZnO	10 µg/mL	48 h	-13,58833	6,80395	-1,99712	1,00E+00	0,05	0	-41,20121	14,02454
Fe:ZnO	20 µg/mL	48 h	ZnO	10 µg/mL	72 h	-6,58618	6,80395	-0,96799	1,00E+00	0,05	0	-34,19906	21,0267
Fe:ZnO	20 µg/mL	48 h	ZnO	15 µg/mL	24 h	-37,44815	6,80395	-5,50388	0,0003924	0,05	1	-65,06103	-9,83527
Fe:ZnO	20 µg/mL	48 h	ZnO	15 µg/mL	48 h	-38,0915	6,80395	-5,59844	0,0002824	0,05	1	-65,70438	-10,47863
Fe:ZnO	20 µg/mL	48 h	ZnO	15 µg/mL	72 h	-10,63676	6,80395	-1,56332	1	0,05	0	-38,24963	16,97612
Fe:ZnO	20 µg/mL	48 h	ZnO	20 µg/mL	24 h	47,45725	6,80395	6,97496	2,217E-06	0,05	1	19,84438	75,07013
Fe:ZnO	20 µg/mL	48 h	ZnO	20 µg/mL	48 h	69,18001	6,80395	10,16763	4,045E-11	0,05	1	41,56714	96,79289
Fe:ZnO	20 µg/mL	48 h	ZnO	20 µg/mL	72 h	66,5038	6,80395	9,77429	1,468E-10	0,05	1	38,89092	94,11667
Fe:ZnO	20 µg/mL	48 h	ZnO	25 µg/mL	24 h	90,56007	6,80395	13,30993	2,754E-15	0,05	1	62,9472	118,17295
Fe:ZnO	20 µg/mL	48 h	ZnO	25 µg/mL	48 h	98,19716	6,80395	14,43238	1,211E-16	0,05	1	70,58429	125,81004
Fe:ZnO	20 µg/mL	48 h	ZnO	25 µg/mL	72 h	98,15203	6,80395	14,42574	1,233E-16	0,05	1	70,53915	125,76491
Fe:ZnO	20 µg/mL	72 h	Fe:ZnO	25 µg/mL	24 h	35,44704	6,80395	5,20977	0,00109	0,05	1	7,83417	63,05992
Fe:ZnO	20 µg/mL	72 h	Fe:ZnO	25 µg/mL	48 h	55,65482	6,80395	8,17978	3,256E-08	0,05	1	28,04194	83,26769
Fe:ZnO	20 µg/mL	72 h	Fe:ZnO	25 µg/mL	72 h	49,74812	6,80395	7,31165	6,765E-07	0,05	1	22,13524	77,36099
Fe:ZnO	20 µg/mL	72 h	ZnO	10 µg/mL	24 h	-31,83866	6,80395	-4,67944	6,57E-03	0,05	1	-59,45154	-4,22579
Fe:ZnO	20 µg/mL	72 h	ZnO	10 µg/mL	48 h	-38,324	6,80395	-5,63261	2,51E-04	0,05	1	-65,93687	-10,71112
Fe:ZnO	20 µg/mL	72 h	ZnO	10 µg/mL	72 h	-31,32184	6,80395	-4,60348	8,46E-03	0,05	1	-58,93472	-3,70897
Fe:ZnO	20 µg/mL	72 h	ZnO	15 µg/mL	24 h	-62,18381	6,80395	-9,13937	1,222E-09	0,05	1	-89,79669	-34,57094
Fe:ZnO	20 µg/mL	72 h	ZnO	15 µg/mL	48 h	-62,82716	6,80395	-9,23393	8,887E-10	0,05	1	-90,44004	-35,21429
Fe:ZnO	20 µg/mL	72 h	ZnO	15 µg/mL	72 h	-35,37242	6,80395	-5,19881	0,00113	0,05	1	-62,98529	-7,75954
Fe:ZnO	20 µg/mL	72 h	ZnO	20 µg/mL	24 h	22,72159	6,80395	3,33947	4,50E-01	0,05	0	-4,89129	50,33447
Fe:ZnO	20 µg/mL	72 h	ZnO	20 µg/mL	48 h	44,44435	6,80395	6,53214	1,06E-05	0,05	1	16,83148	72,05723
Fe:ZnO	20 µg/mL	72 h	ZnO	20 µg/mL	72 h	41,76813	6,80395	6,13881	4,25E-05	0,05	1	14,15526	69,38101
Fe:ZnO	20 µg/mL	72 h	ZnO	25 µg/mL	24 h	65,82441	6,80395	9,67444	2,04E-10	0,05	1	38,21153	93,43729
Fe:ZnO	20 µg/mL	72 h	ZnO	25 µg/mL	48 h	73,4615	6,80395	10,79689	5,35E-12	0,05	1	45,84862	101,07438
Fe:ZnO	20 µg/mL	72 h	ZnO	25 µg/mL	72 h	73,41637	6,80395	10,79026	5,47E-12	0,05	1	45,80349	101,02925
Fe:ZnO	25 µg/mL	24 h	Fe:ZnO	25 µg/mL	48 h	20,20777	6,80395	2,97001	1	0,05	0	-7,4051	47,82065
Fe:ZnO	25 µg/mL	24 h	Fe:ZnO	25 µg/mL	72 h	14,30107	6,80395	2,10188	1	0,05	0	-13,3118	41,91395
Fe:ZnO	25 µg/mL	24 h	ZnO	10 µg/mL	24 h	-67,28571	6,80395	-9,88921	1,01E-10	0,05	1	-94,89858	-39,67283
Fe:ZnO	25 µg/mL	24 h	ZnO	10 µg/mL	48 h	-73,77104	6,80395	-10,84239	4,63E-12	0,05	1	-101,38392	-46,15816

Fe:ZnO	25 µg/mL	24 h	ZnO	10 µg/mL	72 h	-66,76889	6,80395	-9,81325	1,29E-10	0,05	1	-94,38176	-39,15601
Fe:ZnO	25 µg/mL	24 h	ZnO	15 µg/mL	24 h	-97,63086	6,80395	-14,34915	1,519E-16	0,05	1	-125,24373	-70,01798
Fe:ZnO	25 µg/mL	24 h	ZnO	15 µg/mL	48 h	-98,27421	6,80395	-14,4437	1,17E-16	0,05	1	-125,88709	-70,66133
Fe:ZnO	25 µg/mL	24 h	ZnO	15 µg/mL	72 h	-70,81946	6,80395	-10,40858	1,85E-11	0,05	1	-98,43234	-43,20659
Fe:ZnO	25 µg/mL	24 h	ZnO	20 µg/mL	24 h	-12,72545	6,80395	-1,8703	1	0,05	0	-40,33833	14,88742
Fe:ZnO	25 µg/mL	24 h	ZnO	20 µg/mL	48 h	8,99731	6,80395	1,32237	1	0,05	0	-18,61557	36,61018
Fe:ZnO	25 µg/mL	24 h	ZnO	20 µg/mL	72 h	6,32109	6,80395	0,92903	1	0,05	0	-21,29179	33,93397
Fe:ZnO	25 µg/mL	24 h	ZnO	25 µg/mL	24 h	30,37737	6,80395	4,46467	0,0134	0,05	1	2,76449	57,99024
Fe:ZnO	25 µg/mL	24 h	ZnO	25 µg/mL	48 h	38,01446	6,80395	5,58712	0,0002938	0,05	1	10,40158	65,62733
Fe:ZnO	25 µg/mL	24 h	ZnO	25 µg/mL	72 h	37,96932	6,80395	5,58048	0,0003006	0,05	1	10,35645	65,5822
Fe:ZnO	25 µg/mL	48 h	Fe:ZnO	25 µg/mL	72 h	-5,9067	6,80395	-0,86813	1	0,05	0	-33,51958	21,70618
Fe:ZnO	25 µg/mL	48 h	ZnO	10 µg/mL	24 h	-87,49348	6,80395	-12,85922	1,01E-14	0,05	1	-115,10636	-59,88061
Fe:ZnO	25 µg/mL	48 h	ZnO	10 µg/mL	48 h	-93,97881	6,80395	-13,81239	6,67E-16	0,05	1	-121,59169	-66,36594
Fe:ZnO	25 µg/mL	48 h	ZnO	10 µg/mL	72 h	-86,97666	6,80395	-12,78326	1,26E-14	0,05	1	-114,58954	-59,36378
Fe:ZnO	25 µg/mL	48 h	ZnO	15 µg/mL	24 h	-117,83863	6,80395	-17,31915	7,658E-20	0,05	1	-145,45151	-90,22576
Fe:ZnO	25 µg/mL	48 h	ZnO	15 µg/mL	48 h	-118,48198	6,80395	-17,41371	6,11E-20	0,05	1	-146,09486	-90,86911
Fe:ZnO	25 µg/mL	48 h	ZnO	15 µg/mL	72 h	-91,02724	6,80395	-13,37859	2,26E-15	0,05	1	-118,64011	-63,41436
Fe:ZnO	25 µg/mL	48 h	ZnO	20 µg/mL	24 h	-32,93323	6,80395	-4,84031	0,00382	0,05	1	-60,5461	-5,32035
Fe:ZnO	25 µg/mL	48 h	ZnO	20 µg/mL	48 h	-11,21047	6,80395	-1,64764	1	0,05	0	-38,82334	16,40241
Fe:ZnO	25 µg/mL	48 h	ZnO	20 µg/mL	72 h	-13,88668	6,80395	-2,04097	1	0,05	0	-41,49956	13,72619
Fe:ZnO	25 µg/mL	48 h	ZnO	25 µg/mL	24 h	10,16959	6,80395	1,49466	1	0,05	0	-17,44328	37,78247
Fe:ZnO	25 µg/mL	48 h	ZnO	25 µg/mL	48 h	17,80668	6,80395	2,61711	1	0,05	0	-9,80619	45,41956
Fe:ZnO	25 µg/mL	48 h	ZnO	25 µg/mL	72 h	17,76155	6,80395	2,61048	1	0,05	0	-9,85133	45,37443
Fe:ZnO	25 µg/mL	72 h	ZnO	10 µg/mL	24 h	-81,58678	6,80395	-11,99109	1,32E-13	0,05	1	-109,19966	-53,9739
Fe:ZnO	25 µg/mL	72 h	ZnO	10 µg/mL	48 h	-88,07211	6,80395	-12,94426	7,88E-15	0,05	1	-115,68499	-60,45924
Fe:ZnO	25 µg/mL	72 h	ZnO	10 µg/mL	72 h	-81,06996	6,80395	-11,91513	1,67E-13	0,05	1	-108,68283	-53,45708
Fe:ZnO	25 µg/mL	72 h	ZnO	15 µg/mL	24 h	-111,93193	6,80395	-16,45102	6,377E-19	0,05	1	-139,54481	-84,31905
Fe:ZnO	25 µg/mL	72 h	ZnO	15 µg/mL	48 h	-112,57528	6,80395	-16,54558	5,04E-19	0,05	1	-140,18816	-84,96241
Fe:ZnO	25 µg/mL	72 h	ZnO	15 µg/mL	72 h	-85,12053	6,80395	-12,51046	2,81E-14	0,05	1	-112,73341	-57,50766
Fe:ZnO	25 µg/mL	72 h	ZnO	20 µg/mL	24 h	-27,02653	6,80395	-3,97218	0,0657	0,05	0	-54,6394	0,58635
Fe:ZnO	25 µg/mL	72 h	ZnO	20 µg/mL	48 h	-5,30376	6,80395	-0,77951	1	0,05	0	-32,91664	22,30911
Fe:ZnO	25 µg/mL	72 h	ZnO	20 µg/mL	72 h	-7,97998	6,80395	-1,17285	1	0,05	0	-35,59286	19,63289
Fe:ZnO	25 µg/mL	72 h	ZnO	25 µg/mL	24 h	16,07629	6,80395	2,36279	1	0,05	0	-11,53658	43,68917
Fe:ZnO	25 µg/mL	72 h	ZnO	25 µg/mL	48 h	23,71338	6,80395	3,48524	0,2928	0,05	0	-3,89949	51,32626
Fe:ZnO	25 µg/mL	72 h	ZnO	25 µg/mL	72 h	23,66825	6,80395	3,4786	0,29864	0,05	0	-3,94462	51,28113
ZnO	10 µg/mL	24 h	ZnO	10 µg/mL	48 h	-6,48533	6,80395	-0,95317	1	0,05	0	-34,09821	21,12754
ZnO	10 µg/mL	24 h	ZnO	10 µg/mL	72 h	0,51682	6,80395	0,07596	1,00E+00	0,05	0	-27,09605	28,1297
ZnO	10 µg/mL	24 h	ZnO	15 µg/mL	24 h	-30,34515	6,80395	-4,45993	1,36E-02	0,05	1	-57,95803	-2,73227
ZnO	10 µg/mL	24 h	ZnO	15 µg/mL	48 h	-30,9885	6,80395	-4,55449	9,96E-03	0,05	1	-58,60138	-3,37562
ZnO	10 µg/mL	24 h	ZnO	15 µg/mL	72 h	-3,53375	6,80395	-0,51937	1,00E+00	0,05	0	-31,14663	24,07912
ZnO	10 µg/mL	24 h	ZnO	20 µg/mL	24 h	54,56025	6,80395	8,01891	5,69E-08	0,05	1	26,94738	82,17313

ZnO	10 µg/mL	24 h	ZnO	20 µg/mL	48 h	76,28302	6,80395	11,21158	1,45E-12	0,05	1	48,67014	103,89589
ZnO	10 µg/mL	24 h	ZnO	20 µg/mL	72 h	73,6068	6,80395	10,81825	5,00E-12	0,05	1	45,99392	101,21967
ZnO	10 µg/mL	24 h	ZnO	25 µg/mL	24 h	97,66307	6,80395	14,35388	1,50E-16	0,05	1	70,0502	125,27595
ZnO	10 µg/mL	24 h	ZnO	25 µg/mL	48 h	105,30016	6,80395	15,47633	7,58E-18	0,05	1	77,68729	132,91304
ZnO	10 µg/mL	24 h	ZnO	25 µg/mL	72 h	105,25503	6,80395	15,4697	7,71E-18	0,05	1	77,64216	132,86791
ZnO	10 µg/mL	48 h	ZnO	10 µg/mL	72 h	7,00215	6,80395	1,02913	1	0,05	0	-20,61072	34,61503
ZnO	10 µg/mL	48 h	ZnO	15 µg/mL	24 h	-23,85982	6,80395	-3,50676	2,75E-01	0,05	0	-51,47269	3,75306
ZnO	10 µg/mL	48 h	ZnO	15 µg/mL	48 h	-24,50317	6,80395	-3,60132	2,07E-01	0,05	0	-52,11604	3,10971
ZnO	10 µg/mL	48 h	ZnO	15 µg/mL	72 h	2,95158	6,80395	0,4338	1,00E+00	0,05	0	-24,6613	30,56446
ZnO	10 µg/mL	48 h	ZnO	20 µg/mL	24 h	61,04559	6,80395	8,97208	2,15E-09	0,05	1	33,43271	88,65846
ZnO	10 µg/mL	48 h	ZnO	20 µg/mL	48 h	82,76835	6,80395	12,16475	7,85E-14	0,05	1	55,15547	110,38122
ZnO	10 µg/mL	48 h	ZnO	20 µg/mL	72 h	80,09213	6,80395	11,77142	2,58E-13	0,05	1	52,47925	107,70501
ZnO	10 µg/mL	48 h	ZnO	25 µg/mL	24 h	104,14841	6,80395	15,30705	1,18E-17	0,05	1	76,53553	131,76128
ZnO	10 µg/mL	48 h	ZnO	25 µg/mL	48 h	111,7855	6,80395	16,4295	6,73E-19	0,05	1	84,17262	139,39837
ZnO	10 µg/mL	48 h	ZnO	25 µg/mL	72 h	111,74037	6,80395	16,42287	6,84E-19	0,05	1	84,12749	139,35324
ZnO	10 µg/mL	72 h	ZnO	15 µg/mL	24 h	-30,86197	6,80395	-4,53589	1,06E-02	0,05	1	-58,47485	-3,2491
ZnO	10 µg/mL	72 h	ZnO	15 µg/mL	48 h	-31,50532	6,80395	-4,63045	7,73E-03	0,05	1	-59,1182	-3,89245
ZnO	10 µg/mL	72 h	ZnO	15 µg/mL	72 h	-4,05058	6,80395	-0,59533	1,00E+00	0,05	0	-31,66345	23,5623
ZnO	10 µg/mL	72 h	ZnO	20 µg/mL	24 h	54,04343	6,80395	7,94295	7,41E-08	0,05	1	26,43056	81,65631
ZnO	10 µg/mL	72 h	ZnO	20 µg/mL	48 h	75,76619	6,80395	11,13562	1,84E-12	0,05	1	48,15332	103,37907
ZnO	10 µg/mL	72 h	ZnO	20 µg/mL	72 h	73,08998	6,80395	10,74229	6,37E-12	0,05	1	45,4771	100,70285
ZnO	10 µg/mL	72 h	ZnO	25 µg/mL	24 h	97,14625	6,80395	14,27792	1,84E-16	0,05	1	69,53338	124,75913
ZnO	10 µg/mL	72 h	ZnO	25 µg/mL	48 h	104,78334	6,80395	15,40037	9,24E-18	0,05	1	77,17047	132,39622
ZnO	10 µg/mL	72 h	ZnO	25 µg/mL	72 h	104,73821	6,80395	15,39374	9,40E-18	0,05	1	77,12533	132,35109
ZnO	15 µg/mL	24 h	ZnO	15 µg/mL	48 h	-0,64335	6,80395	-0,09456	1	0,05	0	-28,25623	26,96952
ZnO	15 µg/mL	24 h	ZnO	15 µg/mL	72 h	26,8114	6,80395	3,94056	0,07258	0,05	0	-0,80148	54,42427
ZnO	15 µg/mL	24 h	ZnO	20 µg/mL	24 h	84,9054	6,80395	12,47884	3,08E-14	0,05	1	57,29253	112,51828
ZnO	15 µg/mL	24 h	ZnO	20 µg/mL	48 h	106,62817	6,80395	15,67151	4,58E-18	0,05	1	79,01529	134,24104
ZnO	15 µg/mL	24 h	ZnO	20 µg/mL	72 h	103,95195	6,80395	15,27818	1,271E-17	0,05	1	76,33907	131,56482
ZnO	15 µg/mL	24 h	ZnO	25 µg/mL	24 h	128,00822	6,80395	18,81381	2,38E-21	0,05	1	100,39535	155,6211
ZnO	15 µg/mL	24 h	ZnO	25 µg/mL	48 h	135,64531	6,80395	19,93626	2,01E-22	0,05	1	108,03244	163,25819
ZnO	15 µg/mL	24 h	ZnO	25 µg/mL	72 h	135,60018	6,80395	19,92963	2,03E-22	0,05	1	107,98731	163,21306
ZnO	15 µg/mL	48 h	ZnO	15 µg/mL	72 h	27,45475	6,80395	4,03512	0,05383	0,05	0	-0,15813	55,06762
ZnO	15 µg/mL	48 h	ZnO	20 µg/mL	24 h	85,54875	6,80395	12,5734	2,33E-14	0,05	1	57,93588	113,16163
ZnO	15 µg/mL	48 h	ZnO	20 µg/mL	48 h	107,27152	6,80395	15,76607	3,59E-18	0,05	1	79,65864	134,88439
ZnO	15 µg/mL	48 h	ZnO	20 µg/mL	72 h	104,5953	6,80395	15,37273	9,93E-18	0,05	1	76,98242	132,20817
ZnO	15 µg/mL	48 h	ZnO	25 µg/mL	24 h	128,65158	6,80395	18,90837	1,92E-21	0,05	1	101,0387	156,26445
ZnO	15 µg/mL	48 h	ZnO	25 µg/mL	48 h	136,28867	6,80395	20,03082	1,64E-22	0,05	1	108,67579	163,90154
ZnO	15 µg/mL	48 h	ZnO	25 µg/mL	72 h	136,24353	6,80395	20,02418	1,66E-22	0,05	1	108,63066	163,85641
ZnO	15 µg/mL	72 h	ZnO	20 µg/mL	24 h	58,09401	6,80395	8,53828	9,45E-09	0,05	1	30,48113	85,70688
ZnO	15 µg/mL	72 h	ZnO	20 µg/mL	48 h	79,81677	6,80395	11,73095	2,92E-13	0,05	1	52,20389	107,42965

ZnO	15 µg/mL	72 h	ZnO	20 µg/mL	72 h	77,14055	6,80395	11,33761	9,80E-13	0,05	1	49,52768	104,7534 3
ZnO	15 µg/mL	72 h	ZnO	25 µg/mL	24 h	101,19683	6,80395	14,87325	3,70E-17	0,05	1	73,58395	128,8097
ZnO	15 µg/mL	72 h	ZnO	25 µg/mL	48 h	108,83392	6,80395	15,9957	2,00E-18	0,05	1	81,22104	136,4467 9
ZnO	15 µg/mL	72 h	ZnO	25 µg/mL	72 h	108,78879	6,80395	15,98906	2,03E-18	0,05	1	81,17591	136,4016 6
ZnO	20 µg/mL	24 h	ZnO	20 µg/mL	48 h	21,72276	6,80395	3,19267	0,68685	0,05	0	-5,89011	49,33564
ZnO	20 µg/mL	24 h	ZnO	20 µg/mL	72 h	19,04654	6,80395	2,79934	1	0,05	0	-8,56633	46,65942
ZnO	20 µg/mL	24 h	ZnO	25 µg/mL	24 h	43,10282	6,80395	6,33497	2,126E-05	0,05	1	15,48994	70,7157
ZnO	20 µg/mL	24 h	ZnO	25 µg/mL	48 h	50,73991	6,80395	7,45742	4,052E-07	0,05	1	23,12703	78,35279
ZnO	20 µg/mL	24 h	ZnO	25 µg/mL	72 h	50,69478	6,80395	7,45079	4,148E-07	0,05	1	23,0819	78,30766
ZnO	20 µg/mL	48 h	ZnO	20 µg/mL	72 h	-2,67622	6,80395	-0,39333	1	0,05	0	-30,28909	24,93666
ZnO	20 µg/mL	48 h	ZnO	25 µg/mL	24 h	21,38006	6,80395	3,1423	0,7924	0,05	0	-6,23282	48,99293
ZnO	20 µg/mL	48 h	ZnO	25 µg/mL	48 h	29,01715	6,80395	4,26475	0,02576	0,05	1	1,40427	56,63002
ZnO	20 µg/mL	48 h	ZnO	25 µg/mL	72 h	28,97202	6,80395	4,25812	0,02632	0,05	1	1,35914	56,58489
ZnO	20 µg/mL	72 h	ZnO	25 µg/mL	24 h	24,05628	6,80395	3,53563	0,25189	0,05	0	-3,5566	51,66915
ZnO	20 µg/mL	72 h	ZnO	25 µg/mL	48 h	31,69337	6,80395	4,65808	0,00705	0,05	1	4,08049	59,30624
ZnO	20 µg/mL	72 h	ZnO	25 µg/mL	72 h	31,64823	6,80395	4,65145	0,00721	0,05	1	4,03536	59,26111
ZnO	25 µg/mL	24 h	ZnO	25 µg/mL	48 h	7,63709	6,80395	1,12245	1	0,05	0	-19,97579	35,24997
ZnO	25 µg/mL	24 h	ZnO	25 µg/mL	72 h	7,59196	6,80395	1,11582	1	0,05	0	-20,02092	35,20483
ZnO	25 µg/mL	48 h	ZnO	25 µg/mL	72 h	-0,04513	6,80395	-0,00663	1	0,05	0	-27,65801	27,56774

Statistical analysis for ZnO and Fe:ZnO uptake on BxPC-3 cell line. Bonferroni t-test (Three Ways ANOVA)

NPs and Dose

	MeanDiff	SEM	t Value	Prob	Alpha	Sig	LCL	UCL
Fe:ZnO 15 ug/mL Fe:ZnO 10 ug/mL	34,78833	3,93795	8,83413	4,03E-06	0,05	1	23,84292	45,73375
ZnO 10 ug/mL Fe:ZnO 10 ug/mL	-48,9917	3,93795	-12,4409	9,70E-08	0,05	1	-59,9371	-38,0463
ZnO 10 ug/mL Fe:ZnO 15 ug/mL	-83,78	3,93795	-21,2751	2,03E-10	0,05	1	-94,7254	-72,8346

Incubation time

	MeanDiff	SEM	t Value	Prob	Alpha	Sig	LCL	UCL
24 h 5 h	15,63222	3,21532	4,86179	3,90E-04	0,05	1	8,62664	22,6378

Interactions

NPs and Dose	Incubation time	NPs and Dose	Incubation time	MeanDiff	SEM	t Value	Prob	Alpha	Sig	LCL	UCL
Fe:ZnO 10 ug/mL	24 h	Fe:ZnO 10 ug/mL	5 h	25,71667	5,5691	4,61774	0,00889	0,05	1	5,39565	46,03768
Fe:ZnO 15 ug/mL	5 h	Fe:ZnO 10 ug/mL	5 h	41,50667	5,5691	7,45303	1,16E-04	0,05	1	21,18565	61,82768
Fe:ZnO 15 ug/mL	5 h	Fe:ZnO 10 ug/mL	24 h	15,79	5,5691	2,83529	0,22541	0,05	0	-4,53102	36,11102
Fe:ZnO 15 ug/mL	24 h	Fe:ZnO 10 ug/mL	5 h	53,78667	5,5691	9,65806	7,81E-06	0,05	1	33,46565	74,10768
Fe:ZnO 15 ug/mL	24 h	Fe:ZnO 10 ug/mL	24 h	28,07	5,5691	5,04031	0,00434	0,05	1	7,74898	48,39102
Fe:ZnO 15 ug/mL	24 h	Fe:ZnO 15 ug/mL	5 h	12,28	5,5691	2,20503	0,71557	0,05	0	-8,04102	32,60102
ZnO 10 ug/mL	5 h	Fe:ZnO 10 ug/mL	5 h	-40,5833	5,5691	-7,28724	1,45E-04	0,05	1	-60,9044	-20,2623
ZnO 10 ug/mL	5 h	Fe:ZnO 10 ug/mL	24 h	-66,3	5,5691	-11,905	7,93E-07	0,05	1	-86,621	-45,979

ZnO 10 ug/mL	5 h	Fe:ZnO 15 ug/mL	5 h	-82,09	5,5691	-14,7403	7,12E-08	0,05	1	-102,411	-61,769
ZnO 10 ug/mL	5 h	Fe:ZnO 15 ug/mL	24 h	-94,37	5,5691	-16,9453	1,43E-08	0,05	1	-114,691	-74,049
ZnO 10 ug/mL	24 h	Fe:ZnO 10 ug/mL	5 h	-31,6833	5,5691	-5,68913	0,00151	0,05	1	-52,0044	-11,3623
ZnO 10 ug/mL	24 h	Fe:ZnO 10 ug/mL	24 h	-57,4	5,5691	-10,3069	3,87E-06	0,05	1	-77,721	-37,079
ZnO 10 ug/mL	24 h	Fe:ZnO 15 ug/mL	5 h	-73,19	5,5691	-13,1422	2,62E-07	0,05	1	-93,511	-52,869
ZnO 10 ug/mL	24 h	Fe:ZnO 15 ug/mL	24 h	-85,47	5,5691	-15,3472	4,49E-08	0,05	1	-105,791	-65,149
ZnO 10 ug/mL	24 h	ZnO 10 ug/mL	5 h	8,9	5,5691	1,5981	1	0,05	0	-11,421	29,22102

Statistical analysis for Fe:ZnO sonodynamic on BxPC-3 cell line. Bonferroni t-test (Three Ways ANOVA)

Sample

	MeanDiff	SEM	t Value	Prob	Alpha	Sig	LCL	UCL
Fe:ZnO Int+Ext CT	-0,12841	3,24031	-0,03963	1	0,05	0	-8,55758	8,30076
Fe:ZnO Int CT	-9,00453	3,57064	-2,52182	0,05946	0,05	0	-18,293	0,28396
Fe:ZnO Int Fe:ZnO Int+Ext	-8,87612	3,57064	-2,48586	0,06427	0,05	0	-18,1646	0,41237

SW

	MeanDiff	SEM	t Value	Prob	Alpha	Sig	LCL	UCL
SW NO	-21,0953	2,79073	-7,55906	2,02E-07	0,05	1	-26,8989	-15,2916

Interactions

Sample	US	Sample	SW	MeanDiff	SEM	t Value	Prob	Alpha	Sig	LCL	UCL
CT	SW	CT	NO	-15,5167	4,9999	-3,1034	0,08071	0,05	0	-32,065	1,03154
Fe:ZnO Int+Ext	NO	CT	NO	-2,6837	4,9999	-0,53675	1	0,05	0	-19,232	13,86454
Fe:ZnO Int+Ext	NO	CT	SW	12,833	3,87291	3,31353	0,04956	0,05	1	0,01478	25,65122
Fe:ZnO Int+Ext	SW	CT	NO	-30,3717	5,91596	-5,13386	6,56E-04	0,05	1	-49,9518	-10,7915
Fe:ZnO Int+Ext	SW	CT	SW	-14,855	4,9999	-2,97106	0,10932	0,05	0	-31,4032	1,69326
Fe:ZnO Int+Ext	SW	Fe:ZnO Int+Ext	NO	-27,688	4,9999	-5,53771	2,56E-04	0,05	1	-44,2362	-11,1397
Fe:ZnO Int	NO	CT	NO	-6,10723	5,53388	-1,10361	1	0,05	0	-24,4228	12,20832
Fe:ZnO Int	NO	CT	SW	9,40947	4,54139	2,07194	0,76165	0,05	0	-5,62122	24,44016
Fe:ZnO Int	NO	Fe:ZnO Int+Ext	NO	-3,42353	4,54139	-0,75385	1	0,05	0	-18,4542	11,60716
Fe:ZnO Int	NO	Fe:ZnO Int+Ext	SW	24,26446	5,53388	4,38471	0,00388	0,05	1	5,9489	42,58001
Fe:ZnO Int	SW	CT	NO	-38,2115	5,91596	-6,45906	3,18E-05	0,05	1	-57,7917	-18,6314
Fe:ZnO Int	SW	CT	SW	-22,6948	4,9999	-4,53906	0,00269	0,05	1	-39,2431	-6,14659
Fe:ZnO Int	SW	Fe:ZnO Int+Ext	NO	-35,5278	4,9999	-7,10571	7,82E-06	0,05	1	-52,0761	-18,9796
Fe:ZnO Int	SW	Fe:ZnO Int+Ext	SW	-7,83985	5,91596	-1,3252	1	0,05	0	-27,42	11,7403
Fe:ZnO Int	SW	Fe:ZnO Int	NO	-32,1043	5,53388	-5,80141	1,39E-04	0,05	1	-50,4199	-13,7888
